# Novel Xanomeline-Containing Bitopic Ligands of Muscarinic Acetylcholine Receptors: Design, Synthesis and FRET Investigation

**DOI:** 10.3390/molecules28052407

**Published:** 2023-03-06

**Authors:** Carlo Matera, Michael Kauk, Davide Cirillo, Marco Maspero, Claudio Papotto, Daniela Volpato, Ulrike Holzgrabe, Marco De Amici, Carsten Hoffmann, Clelia Dallanoce

**Affiliations:** 1 Department of Pharmaceutical Sciences, Medicinal Chemistry Section “Pietro Pratesi”, University of Milan, Via L. Mangiagalli 25, 20133 Milan, Italy; 2 Institute for Molecular Cell Biology, Center for Molecular Biomedicine, University Hospital Jena, Friedrich Schiller University Jena, Hans Knoell Str. 2, 07745 Jena, Germany; 3 Pharmaceutical and Medicinal Chemistry, Institute of Pharmacy and Food Chemistry, University of Würzburg, Am Hubland, 97074 Würzburg, Germany

**Keywords:** muscarinic acetylcholine receptors, Xanomeline, 77-LH-28-1, bitopic hybrid ligands, synthesis, fluorescence resonance energy transfer

## Abstract

In the last few years, fluorescence resonance energy transfer (FRET) receptor sensors have contributed to the understanding of GPCR ligand binding and functional activation. FRET sensors based on muscarinic acetylcholine receptors (mAChRs) have been employed to study dual-steric ligands, allowing for the detection of different kinetics and distinguishing between partial, full, and super agonism. Herein, we report the synthesis of the two series of bitopic ligands, **12-Cn** and **13-Cn,** and their pharmacological investigation at the M_1_, M_2_, M_4_, and M_5_ FRET-based receptor sensors. The hybrids were prepared by merging the pharmacophoric moieties of the M_1_/M_4_-preferring orthosteric agonist Xanomeline **10** and the M_1_-selective positive allosteric modulator 77-LH-28-1 (1-[3-(4-butyl-1-piperidinyl)propyl]-3,4-dihydro-2(1H)-quinolinone) **11**. The two pharmacophores were connected through alkylene chains of different lengths (C3, C5, C7, and C9). Analyzing the FRET responses, the tertiary amine compounds **12-C5**, **12-C7**, and **12-C9** evidenced a selective activation of M_1_ mAChRs, while the methyl tetrahydropyridinium salts **13-C5**, **13-C7**, and **13-C9** showed a degree of selectivity for M_1_ and M_4_ mAChRs. Moreover, whereas hybrids **12-Cn** showed an almost linear response at the M_1_ subtype, hybrids **13-Cn** evidenced a bell-shaped activation response. This different activation pattern suggests that the positive charge anchoring the compound **13-Cn** to the orthosteric site ensues a degree of receptor activation depending on the linker length, which induces a graded conformational interference with the binding pocket closure. These bitopic derivatives represent novel pharmacological tools for a better understanding of ligand-receptor interactions at a molecular level.

## 1. Introduction

In the last few years, different receptor sensors based on the fluorescence resonance energy transfer (FRET) were generated for various G protein-coupled receptors (GPCRs) and represented a valuable tool for investigating real-time receptor activation as well as ligand-receptor interactions [1,2,3,4]. The use of GPCR-based biosensors allows for the study of GPCRs in their native membrane and the exploration of receptor dynamics [5,6]. Indeed, GPCRs behave as a highly dynamic system, transitioning among distinct conformational states, which dictate their signaling pathways through the cell membrane [7]. Analysis of GPCR structural dynamics is therefore essential for understanding their physiology and is quite informative in light of the rational design of GPCR-targeted drugs. 

FRET receptor sensors made a decisive contribution to the overall understanding of GPCR dynamics in the context of ligand binding and functional activation [5,8]. Moreover, these sensors have been used not only to detect ligand agonistic properties but also to investigate the conformational changes produced by receptor allosteric modulators [8]. The allosteric binding regions on GPCRs are outside the orthosteric binding domain and were found to be less conserved among receptor subtypes [9]. GPCR allosteric modulators have a great potential for selective ligand development. Additionally, in the attempt to achieve subtype selectivity, allosteric building blocks may be fused with orthosteric receptor activators to give dual-steric (i.e., orthosteric/allosteric) GPCR ligands [10], which couple the high affinity receptor activation from the orthosteric site with the allosteric control of subtype and signaling pathway selectivity [11].

**Figure 1:**
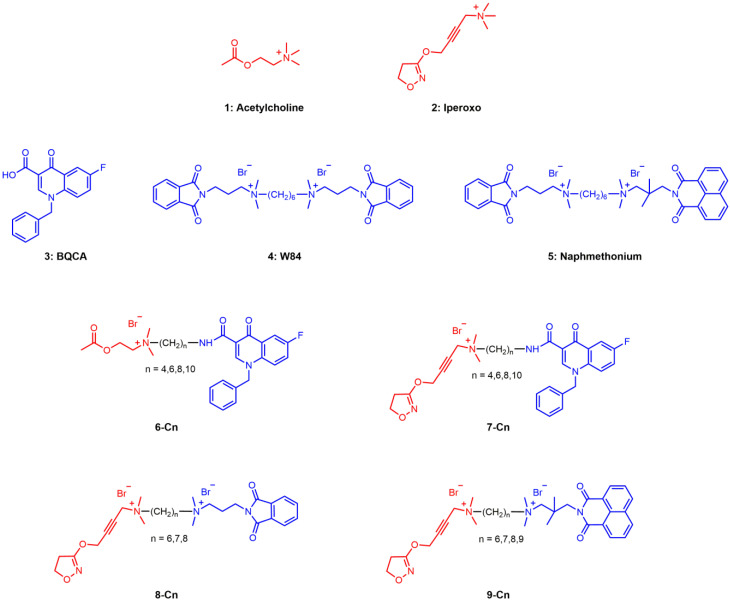
Structures of muscarinic orthosteric ligands (**1**,**2**), allosteric modulators (**3**,**4**,**5**), and dual-steric hybrid compounds ACh/BQCA (**6-Cn**), Iperoxo/BQCA (**7-Cn**), Iperoxo/W84 (**8-Cn**), and Iperoxo/Naphmethonium (**9-Cn**) investigated by using muscarinic FRET receptor sensors.

FRET sensors based on muscarinic acetylcholine receptors (mAChRs) have been used to study dual-steric ligands, leading to the detection of different kinetics and distinguishing between partial, full, and super agonism [12,13,14,15]. In particular, this analysis was performed on the two series of bitopic ligands, **6-Cn** and **7-Cn** (Figure 1), designed for a selective interaction with M_1_ mAChRs by using an M_1_ receptor FRET sensor [12]. In these hybrids, the orthosteric moieties of the endogenous ligand acetylcholine (ACh) **1** and of the super agonist Iperoxo **2** [16,17,18,19] were respectively connected with the highly selective M_1_ positive allosteric modulator benzyl quinolone carboxylic acid (BQCA) **3** [20] by means of alkyl chains of different length. An optimal linker length of six methylene groups was found to engender a maximal response at M_1_ mAChRs, whereas an unprecedented conformational change for GPCRs was evidenced when these bitopic ligands were endowed with a longer spacer. Moreover, FRET biosensors were employed to investigate the conformational changes induced at M_1_ and M_2_ mAChRs by the dual-steric compounds **8-Cn** and **9-Cn** (Figure 1), which are composed of Iperoxo **2** and the fragments, respectively, of the two negative allosteric modulators W84 **4** [21] and Naphmethonium **5** [22] covalently connected by a polymethylene chain of varying length. Previously, compounds **8-Cn** and **9-Cn** were used as chemical probes to investigate the conformational transitions in the allosteric vestibule of the M_2_ mAChR by using an M_2_ FRET sensor. A graded receptor activation from the orthosteric binding site was evidenced, with a simultaneous restriction of spatial flexibility of the allosteric vestibule, which was found to affect the extent of receptor movement and the activation of differential signaling pathways [13]. Recently, both series of bitopic ligands, **8-Cn** and **9-Cn** (Figure 1), were employed to investigate also the M_1_ mAChR activation. Using a biosensor for the M_1_ mAChR, the intracellular conformational changes in the receptor at the G protein-coupling interface were governed by the linker length of the bitopic ligands and allowed selective G protein signaling. Indeed, the decrease in length of the spacer gradually hampered the extracellular binding pocket closure and induced different coupling in distinct G protein families [14].

**Figure 2:**
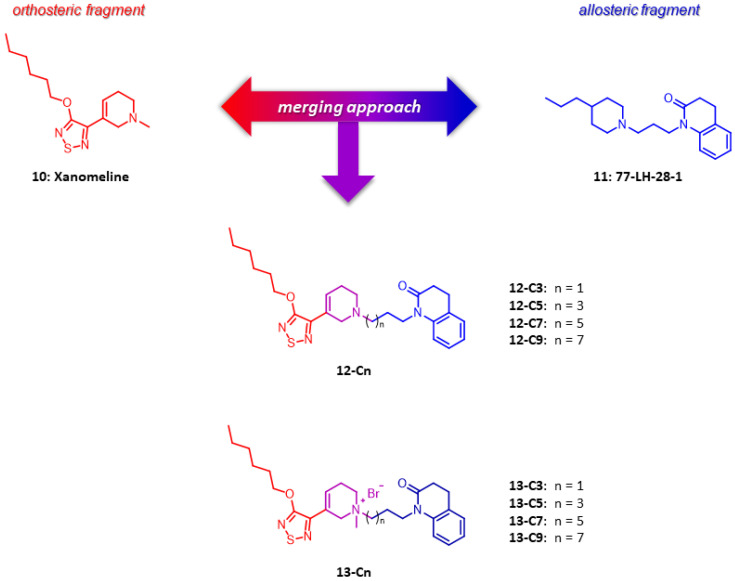
Design of the target Xanomeline/77-LH-28-1 hybrid compounds **12-Cn** (**12-C3**, **12-C5**, **12-C7**, **and 12-C9**) and **13-Cn** (**13-C3**, **13-C5**, **13-C7**, **and 13-C9**) derived from merging the orthosteric ligand Xanomeline (**10**) with the allosteric modulator 77-LH-28-1 (**11**).

As a continuation of our efforts towards the development of innovative bitopic ligands of mAChR subtypes [23,24,25,26,27,28] and to further deepen ligand-receptor interactions at a molecular level, in this study, we designed and synthesized a new set of derivatives, **12-Cn** and **13-Cn** (Figure 2), that integrate with the same molecular skeleton as the pharmacophoric moieties of Xanomeline and 77-LH-28-1 (1-[3-(4-butyl-1-piperidinyl)propyl]-3,4-dihydro-2(1*H*)-quinolinone). Xanomeline is a well-known M_1_/M_4_-preferring orthosteric agonist that ameliorated cognitive impairments in Alzheimer’s disease patients and showed activity in various models of schizophrenia, with a potential benefit for the treatment of positive, negative, and cognitive symptoms [29]. On the other hand, 77-LH-28-1 was characterized as an M_1_-selective, positive allosteric modulator, thus representing an interesting pharmacological tool with cognition-enhancing properties [30]. As illustrated in Figure 2, we designed the novel bipharmacophoric derivatives as merged structures, with the tetrahydropyridine nucleus of Xanomeline as the central core. The target hybrids were designed as tertiary amines, **12-Cn,** and methyl tetrahydropyridinium salts, **13-Cn,** and the distance between the tetrahydropyridine ring and the dihydroquinoline moiety was modulated by varying the length of the chain, from three to nine methylene units, that connects the nitrogen atoms of the two fragments. Herein, we report the synthesis of the new bitopic ligands **12-Cn** and **13-Cn** and their pharmacological characterization by means of FRET-based mAChR sensors.

## 2. Results and Discussion

### 2.1. Synthesis of Hybrid Ligands

**Scheme 1:**
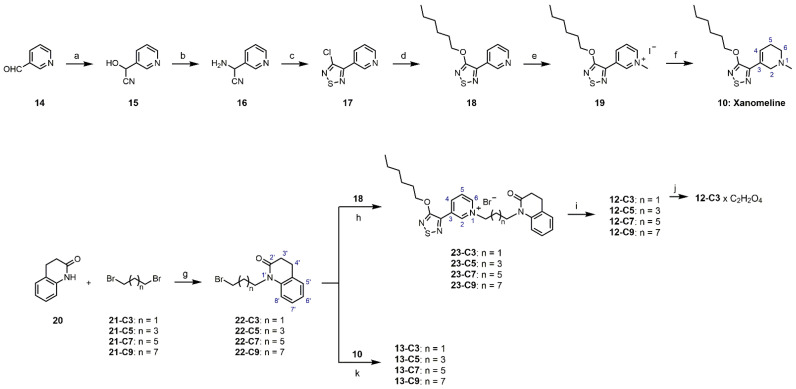
Synthesis of target hybrid compounds **12-Cn** and **13-Cn**. Reagents and conditions*:* (**a**) TMSCN, AcOH, rt, 23 h; (**b**) NH_3_/NH_4_Cl, rt, 22 h; (**c**) S_2_Cl_2_, DMF, 0 °C, 30 min; (**d**) 1-hexanol, NaH, THF, reflux, 3 h; (**e**) CH_3_I, acetone, rt, 22 h; (**f**) NaBH_4_, MeOH, rt, 24 h; (**g**) NaH, DMF, 50 °C, 3 h; (**h**) CH_3_CN, reflux, 7 d; (**k**) CH_3_CN, reflux, 24 h; (**i**) NaBH_4_, EtOH, rt, 24 h; and (**j**) oxalic acid, MeOH, 40 °C, 48 h.

The synthetic approach for the preparation of target hybrids, such as tertiary amines **12-C3**, **12-C5**, **12-C7**, **and 12-C9** and ammonium salts **13-C3**, **13-C5**, **13-C7**, **and 13-C9** are illustrated in Scheme 1. We initially prepared the orthosteric pharmacophoric moiety Xanomeline **10** according to a known procedure [25,31]. In the presence of acetic acid, 3-pyridinecarboxaldehyde **14** underwent a Strecker-type reaction with trimethylsilyl cyanide to afford cyanohydrin **15**. The reaction of **15** with ammonium chloride in ammonium hydroxide yielded the corresponding aminonitrile **16**, which was then cyclized by treatment with disulfur dichloride in DMF and afforded the 3-chloro-4-(pyridine-3-yl)-1,2,5-thiadiazole intermediate **17**. The latter was functionalized by nucleophilic substitution with 1-hexanol using sodium hydride in THF to provide intermediate **18**. Next, Xanomeline **10** was obtained after the conversion of **18** to the corresponding quaternary pyridinium iodide **19** by reaction with methyl iodide in acetone, followed by the reduction in the aromatic positively charged heterocyclic ring to the corresponding 1,2,5,6-tetrahydropyridine by using sodium borohydride in methanol. The monobromo-3,4-dihydroquinolinone allosteric fragments **22-Cn** were obtained through the reaction of the commercially available 3,4-dihydroquinolinone **20** with alkyl dibromides **21-Cn** in the presence of sodium hydride, followed by heating at 50 °C in DMF.

The synthesis of the desired hybrids, **12-C3**, **12-C5**, **12-C7**, and **12-C9,** was achieved through the Menshutkin reaction between intermediate **18** and the bromo derivatives **22-Cn**. The reaction was performed in refluxing acetonitrile and led to the corresponding pyridinium bromides **23-Cn**, which were subsequently reduced with NaBH_4_ in ethanol at room temperature to produce the final compound, **12-Cn,** as tertiary amines. The free base **12-C3** was then reacted with oxalic acid in methanol to provide the related crystalline **12-C3** oxalate; the same treatment of homologs **12-C5**, **12-C7**, and **12-C9** did not afford the corresponding oxalates. The desired positively charged analogs **13-C3**, **13-C5**, **13-C7**, and **13-C9** were obtained through the Menshutkin reaction between Xanomeline **10** and the bromo intermediates **22-Cn** in refluxing acetonitrile. 

### 2.2. Muscarinic Receptor FRET Sensors and Ligand Characterization

In this work, we aimed to investigate the effects of our novel compounds on the muscarinic receptor subtypes that are more abundantly expressed in the brain (M_1_, M_2_, M_4_, and M_5_). For this purpose, we made use of M_1_, M_2_, M_4_, and M_5_ mAChR FRET sensors, which were not truncated but were modified by fusing a cyan fluorescent protein (CFP) to the C-terminus of the receptor and, additionally, by insertion of the fluoresceine arsenical hairpin (FlAsH) binding motif, consisting of the six amino acids CCGPCC sequence, into the third intracellular loop (IL3) region. For FRET experiments, the mAChR sensors were stably expressed in HEK 293 cells. Due to ligand binding, a structural rearrangement of the transmembrane vestibule occurs, with a change in the relative distance and orientation of the fluorophores towards each other that was detected with a millisecond resolution. For ligand application, a pressure-based perfusion system was used. By superfusing the cells with a physiological buffer, a straight baseline occurred. Upon ligand addition (indicated by black bars), a sharp peak was detected with a concentration-dependent intensity. After the signal reached a constant level, superfusion was switched back to buffer, and the signal returned to the baseline. The signal direction is sensor-specific and probably due to the fluorophore orientation within the receptor structure. A slight reduction in the FRET signal over time was detectable and may be attributed to photobleaching. To prevent artificial underestimation of ligand efficacy, reference and ligand compounds were measured alternately.

**Figure 3:**
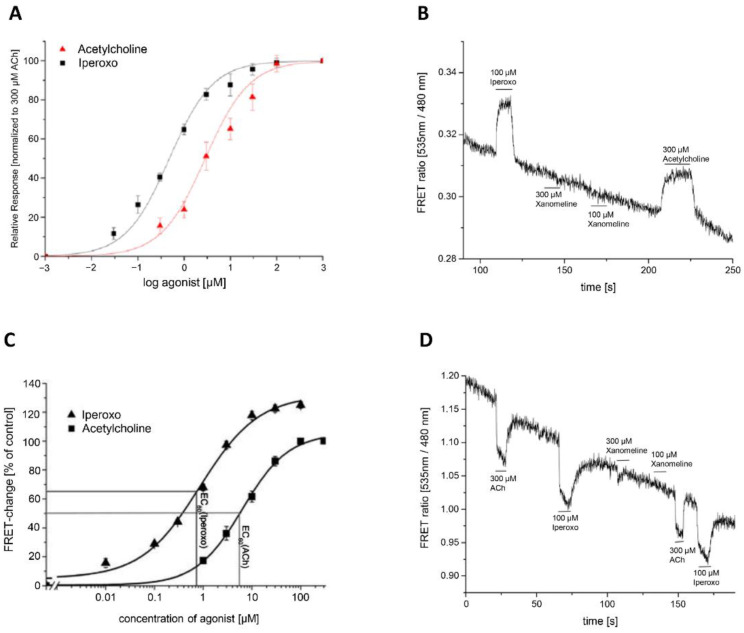
Ligand characterization at the M_1_ and M_2_ muscarinic receptor sensors. (**A**) Concentration response curves of ACh (**1**) and Iperoxo (**2**) as calculated from FRET experiments using the M_1_ receptor sensor. Each ligand concentration is represented by an average value of at least 10 cells; (**B**) a FRET recording of the M_1_ receptor sensor stably expressed in HEK293 cells is illustrated. Additionally, 100 micromolar Iperoxo (**2**), 300 micromolar ACh (**1**), and 100 and 300 micromolar Xanomeline (**10**), were applied as indicated at the appropriate time points by black bars above or below the recorded signal. (**C**) Concentration response curves of ACh (**1**) and Iperoxo (**2**) as calculated from FRET experiments using the M_2_ receptor sensor. Each ligand concentration is represented by the average value of at least 10 cells; (**D**) a FRET recording of the M_2_ receptor sensor stably expressed in HEK293 cells is illustrated. Additionally, 300 micromolar ACh (**1**), 100 micromolar Iperoxo (**2**), and 100 and 300 micromolar Xanomeline (**10**), were applied as indicated at the appropriate time points by black bars below or above the recorded signal.

Initially, we characterized the functional response of the M_1_ and M_2_ receptor FRET sensors by applying the three muscarinic orthosteric ligands, namely, the endogenous neurotransmitter ACh **1** (Figure 1), Iperoxo **2** (Figure 1), and Xanomeline **10** (Figure 2), which represents the orthosteric moiety of the new bitopic ligands. At the M_1_ receptor sensor, both ACh and Iperoxo showed 100% receptor activation, thus behaving as full agonists. We generated concentration-dependent response curves (Figure 3A), and Iperoxo showed a lower EC_50_ value (EC_50_ = 0.5 μM) compared to that of Ach (EC_50_ = 2.8 μM). Conversely, Xanomeline did not show any response. The concentration of Xanomeline was increased up to 300 μM with no effect (Figure 3B), and higher concentrations led to artifacts in the experimental setup. Compared to **1** and **2***,* this lack of response could be due to an alternative binding site for Xanomeline on the M_1_ receptor, which could also suggest an alternative pattern of receptor activation. It was indeed evidenced that Xanomeline shows a noncompetitive binding profile when investigated together with atropine [32,33]. As we explored the mAChR dynamics by introducing a fluorophore below the fifth transmembrane region (TM5) and at the C-terminus, we detected a conformational change mainly in the third intracellular loop. It would be hard to detect ligands that bind but do not activate the receptor, and they are even harder to characterize because strongly biased ligands affect receptor conformations to a higher extent at TM6 than at TM5.

Reference compounds **1** and **2** also behaved as full agonists at the M_2_ FRET sensor. The known super agonism of Iperoxo appeared as a larger conformational change compared to that induced by Ach, and again, the EC_50_ value shown by Iperoxo (EC_50_ = 0.8 μM) was lower than that of ACh (EC_50_ = 6.3 μM) (Figure 3C). Xanomeline did not show any effects at the M_2_ sensor (Figure 3D), as in the case of the M_1_ FRET receptor.

### 2.3. FRET Measurements of Hybrid Ligands

The newly synthesized Xanomeline/77-LH-28-1 hybrid compounds, **12-Cn** and **13-Cn,** were investigated for their ability to induce a conformational change at the mAChRs by using M_1_, M_2_, M_4_, and M_5_ muscarinic FRET sensors. Due to the ligand binding, a structural rearrangement of the receptor transmembrane vestibule occurs, the relative distance of the fluorophores to each other changes, and the dynamic conformational receptor changes are studied in real time with a millisecond resolution. Due to the lack of a receptor response with Xanomeline, the signal from a saturating concentration of Iperoxo was chosen as the reference signal in all the following measurements. 

**Figure 4:**
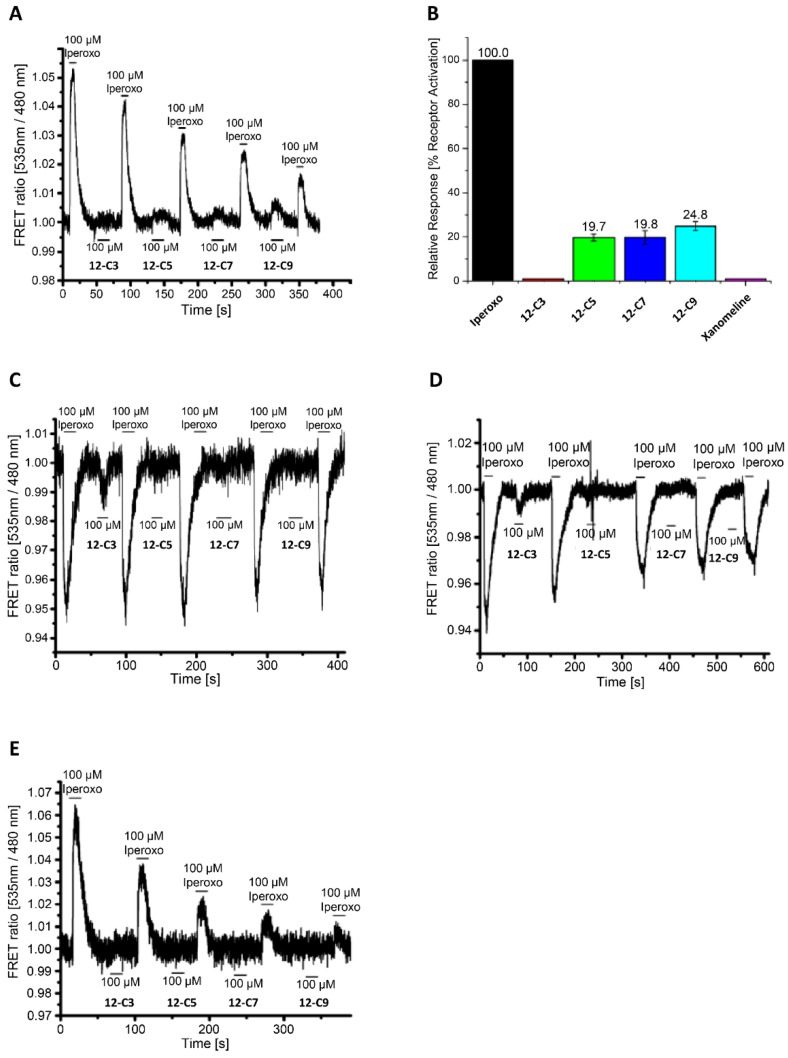
Evaluation of the Xanomeline/77-LH-28-1 hybrid ligands **12-Cn** at the M_1_ (**A**); M_2_ (**C**); M_4_ (**D**); and M_5_ (**E**) muscarinic FRET receptor sensors stably expressed in HEK293 cells. One hundred micromolar Iperoxos (**2**) were used as a reference ligand throughout the recording, as indicated by black bars above the recorded signals. One hundred micromolar concentrations of the hybrid compounds **12-C3**, **12-C5**, **12-C7**, and **12-C9** were applied as indicated at the appropriate time points by black bars below the recorded signal; (**B**) quantified maximal ligand-induced FRET response of compounds **12-Cn** as a percent of control (100 μM Iperoxo) at the M_1_ receptor sensor. Each bar represents the average of at least 10 independent measurements.

The results of the FRET investigation at mAChR sensors for the tertiary amine hybrid derivatives **12-Cn** (Figure 2) are illustrated in Figure 4. Figure 4A,B summarize the response of the M_1_ FRET sensor. The FRET trace in Figure 4A shows that compound **12-C3**, characterized by the shortest linker of three methylene groups, did not induce any conformational change at the receptor sensor. Hybrids **12-C5**, **12-C7**, and **12-C9** were instead able to induce a conformational change with a slightly increasing tendency with longer linker length. Thus, for the M_1_ receptor, we found that a 9-carbon polymethylene spacer is the best linker for the compounds obtained via hybridization of **10** and **11**. This can also be observed in Figure 4B, which shows the maximal ligand-induced changes in comparison with Iperoxo. Conversely, when tested at the M_2_ receptor sensor (Figure 4C), the hybrids **12-C5**, **12-C7,** and **12-C9** did not induce any conformational change, therefore, displaying a first indication of conformational receptor subtype selectivity. Surprisingly, we observed a small conformational variation upon testing **12-C3** at the M_2_ receptor subtype. The same hybrid was also the only ligand of this series that induced a detectable FRET signal at the M_4_ sensor (Figure 4D), at variance with the related homologs **12-C5**, **12-C7,** and **12-C9**. Moreover, the four compounds of the **12-Cn** group were unable to induce a significant FRET signal at the M_5_ receptor subtype (Figure 4E).

**Figure 5:**
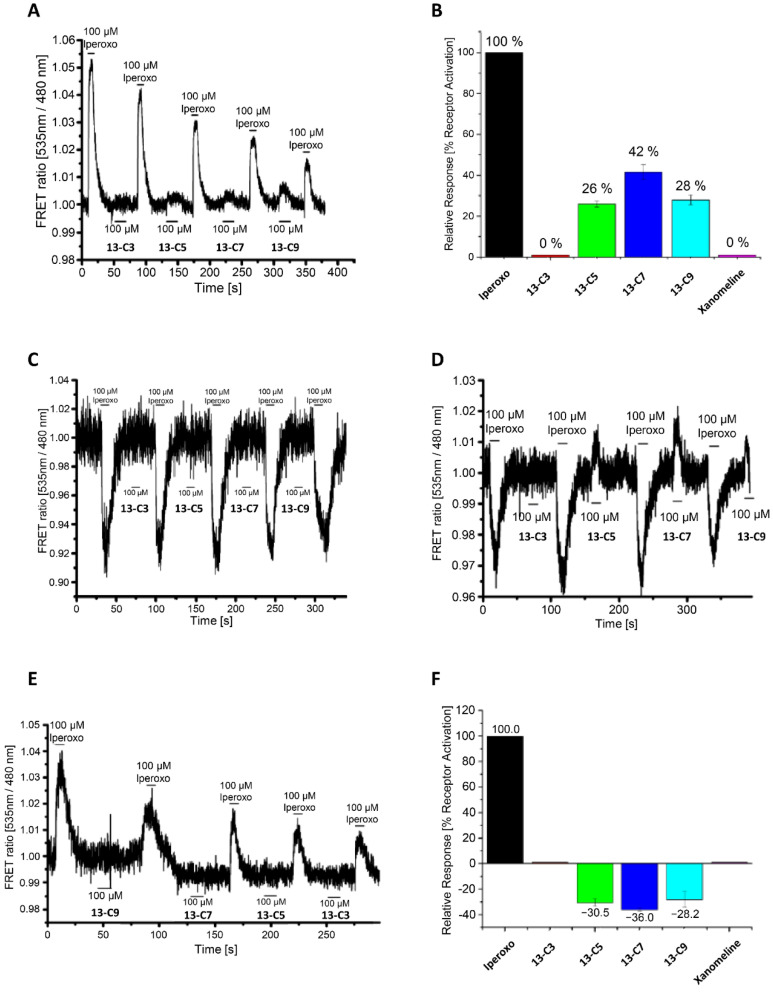
Evaluation of the Xanomeline/77-LH-28-1 hybrid ligands **13-Cn** at the M_1_ (**A**); M_2_ (**C**); M_4_ (**D**); and M_5_ (**E**) muscarinic FRET receptor sensors stably expressed in HEK293 cells. One hundred micromolar Iperoxos (**2**) were used as a reference ligand throughout the recording, as indicated by black bars above the recorded signals. One hundred micromolar concentrations of the hybrid compounds **13-C3**, **13-C5**, **13-C7**, and **13-C9** were applied as indicated at the appropriate time points by black bars below the recorded signal. The quantified maximal ligand-induced FRET response of compounds **13-Cn** as a percent of control (100 μM Iperoxo) at the M_1_ (**B**) and M_4_ (**F**) receptor sensors is shown. Each bar represents the average of at least 10 independent measurements.

The same experiments were performed with the corresponding set of quaternary tetrahydropyridinium salts, **13-Cn** (Figure 2), and the results are displayed in Figure 5. When compounds **13-Cn** were investigated via FRET for their ability to induce a conformational change at the M_1_ receptor, a similar trend (Figure 5A) to that of the uncharged analogs **12-Cn** (Figure 4A) was observed. Whereas hybrid **13-C3** with the shortest linker did not exhibit a FRET signal, the longer linker derivatives **13-C5**, **13-C7,** and **13-C9** induced a conformational change indicative of their agonistic properties. Signal quantification brought about a bell-shaped graph (Figure 5B), since a signal increase was observed for **13-C5** and **13-C7**, followed by a decrease for **13-C9**, characterized by the longest linker. These results clearly evidenced a linker-length-dependent receptor response with an optimal spacer length of approximately seven methylene groups (≈10.8 Å). Hybrid **13-C7** induced the largest conformational change at the M_1_ FRET sensor compared to all the other structural analogs, both of the quaternary ammonium **13-Cn** series and of the tertiary amine **12-Cn** series. The FRET investigation at the M_2_ receptor sensor revealed that the four **13-Cn** compounds were not able to induce any conformational change (Figure 5C), displaying a similar trend to that of the corresponding uncharged set of derivatives **12-Cn** (Figure 4C), the analog **12-C3** excepted. Figure 5D displays a FRET trace of hybrid **13-Cn** at the M_4_ receptor sensor, and the bar graph in Figure 5F summarizes the corresponding FRET signals. Interestingly, at the M_4_ receptor, antiparallel signals for derivatives **13-C5**, **13-C7**, and **13-C9** were detected, with a clear FRET effect. Similar to the results obtained at the M_1_ FRET sensor, the activation pattern showed bell-shaped features. Once again, the signal intensity was dependent on the linker length, with the highest response corresponding to the **13-C7** hybrid containing a spacer with seven methylene groups. Analogously to the tertiary amines, hybrids **12-Cn**, also hybrids **13-Cn** did not affect the M_5_ subtype, and no significant FRET signal was obtained (Figure 5E).

### 2.4. FRET Measurements of Fragments

**Figure 6:**
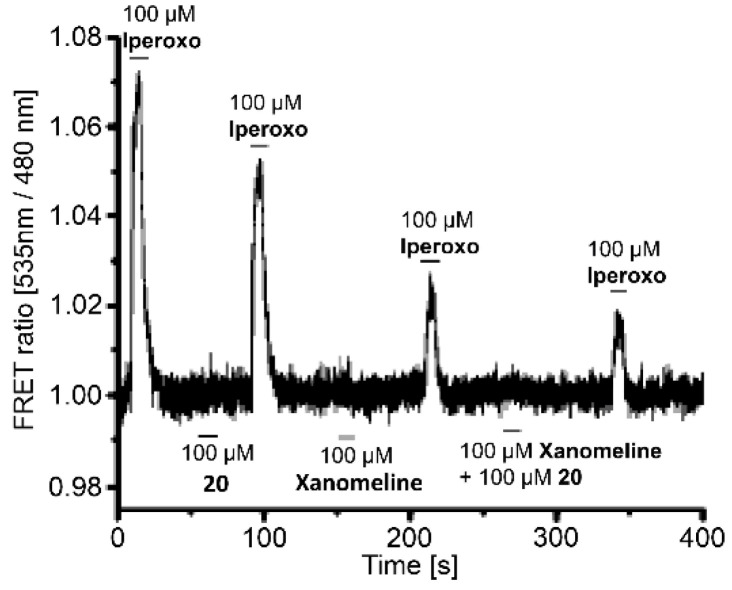
Fragment-based analysis to evaluate the contributions of the single molecular moieties, dihydroquinolinone (**20**), and Xanomeline (**10**), of the Xanomeline/77-LH-28-1 hybrids to the M_1_ FRET signal. A single-cell FRET recording of the M_1_ receptor sensor stably expressed in HEK293 cells is shown. One hundred micromolar Iperoxos were used as a reference ligand throughout the recording, as indicated by black bars above the recorded signal. One hundred micromolars of **20** and **10** were applied separately or together at the appropriate time points, as indicated by black bars below the recorded signal.

A fragment-based screening was applied to unravel the moieties of the hybrid ligands **12-Cn** and **13-Cn** contributing to the observed signals and figure out whether it is possible to reconstruct the signal derived from the hybrids by combining that of the individual orthosteric and allosteric fragments. We chose to perform this study at the M_1_ FRET sensor, which displayed significant FRET signals with both sets of hybrids, **12-Cn** and **13-Cn**. Therefore, Xanomeline **10** and dihydroquinolinone **20** were superfused alone and applied at the same time to the M_1_ receptor-expressing sensor cells (Figure 6). None of the single fragments nor the mixture of the fragments exhibited a detectable effect at the FRET sensor, indicating that the activation patterns observed for hybrids **12-Cn** and **13-Cn** are not a combination of those induced by their molecular component parts. Therefore, we may conclude that the entire structure of ligands **12-Cn** and **13-Cn** is relevant to engendering receptor activation; to this end, the nature and length of the polymethylene spacer represent an essential feature of the molecular skeleton.

### 2.5. Evaluation of Linker Elongation

FRET signals obtained by the investigations of the Xanomeline/77-LH-28-1 hybrids evidenced that an optimal linker length of seven methylene groups induced a remarkable conformational change at the M_1_ mAChR. Indeed, hybrid **13-C7** displayed the most pronounced receptor activation, likely because its linker length best matches the distance between allosteric and orthosteric binding sites, thus favoring the dual-steric (active) binding pose over the purely allosteric pose (inactive). A spacer of shorter or longer length results in a gradual decrease in activation. Shorter linkers likely forbid a productive ligand-receptor binding, and therefore, the related hybrids are not able to trigger a conformational variation in the receptor sensor or they give rise to a reduced activation pattern. Conversely, longer linkers probably cause steric hindrance with the aromatic lid structure of the receptor because of the fixed positive charge of the orthosteric moiety, thus resulting in a lower affinity and a lower receptor response. These findings confirm related data collected in previous investigations performed at the M_1_ mAChR with quinolone-based bitopic ligands **7-Cn** [12] and Iperoxo/W84 hybrids **8-Cn** [14] (Figure 1). A comparable chain of six methylene groups was reported for compounds **7-Cn** as the optimal linker length for conformational changes at the M_1_ mAChR due to the defined distance between the allosteric and the orthosteric binding domains [12,34]. Similarly, the dual-steric compound **8-C8** induced a significantly larger FRET change than the related shorter derivatives (**8-C7** and **8-C6**), which accounts for a reduced conformational interference at the receptor/G protein-coupling interface, resulting in a greater G-protein signaling capability [14]. 

**Figure 7:**
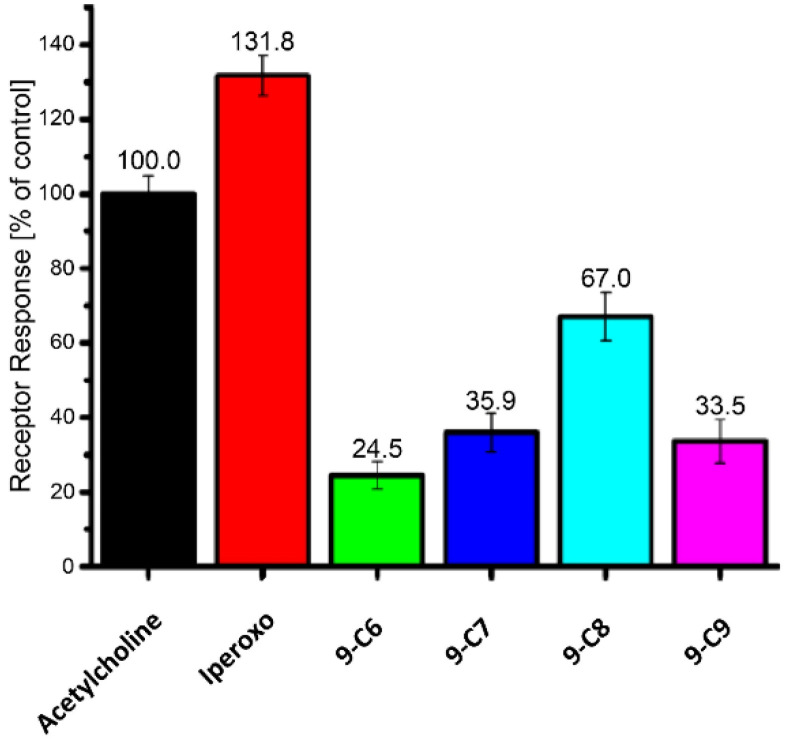
Evaluation of the Iperoxo/Naphmetonium hybrid ligands **9-Cn** at the M_2_ muscarinic FRET receptor sensor stably expressed in HEK293 cells [13].

Moreover, a linker-length-dependent graded activation was evidenced by bitopic ligands **9-Cn** (Figure 1), containing Iperoxo and a fragment of the allosteric modulator Naphmethonium **5**, which were investigated by FRET at the M_2_ mAChR [13]. The best activation profile was displayed by hybrid **9-C8**, characterized by a spacer chain of eight methylene groups. We re-examined the behavior of hybrids **9-C6**, **9-C7**, **and 9-C8** [13] by adding new data obtained on the further elongated bitopic homolog **9-C9** [35]. Even for this group of ligands, FRET measurements evidenced a bell-shaped activation pattern (Figure 7), which resembles that of the positively charged methyl tetrahydropyridinium salt **13-Cn** at the M_1_ mAChR.

These data further confirm that the simultaneous activation of the M_1_ as well as M_2_ receptors by hybrid ligands is modulated/optimized by the length of the spacer connecting the orthosteric and allosteric molecular fragments. The two subtypes may favor variable linker lengths for dual-steric ligands, due to differences in the protein shape and in the distance of their mutual recognition sites [12,13,14].

### 2.6. Receptor Activation Patterns 

The two sets of Xanomeline/77-LH-28-1 hybrid ligands, **12-Cn** and **13-Cn** (Figure 2), were investigated by FRET for their interaction at the M_1_, M_2_, M_4_, and M_5_ mAChRs and evidenced a certain degree of M_1_ subtype selectivity. Interestingly, **12-Cn** and **13-Cn** displayed a different activation pattern at the M_1_ subtype. Whereas the tertiary amine derivatives **12-Cn** showed an almost linear response (Figure 4B), the methyl tetrahydropyridinium derivatives **13-Cn** evidenced a bell-shaped activation response (Figure 5B). The different trend could be explained by taking into account the only structural difference between the two groups of ligands, namely the presence in hybrid **13-Cn** of the permanently positively charged nitrogen, which is missing in the tertiary amine derivatives **12-Cn**. 

The crystal structure of the superagonist Iperoxo **2** bound to the M_2_ muscarinic subtype receptor evidenced a fundamental interaction between the trimethyl ammonium group of **2** and the conserved Asp103 residue within the orthosteric binding site (PDB 4MQS) [36]. A comparable residue, Asp112, served as the counterion for the interaction with the positively charged inverse agonist, Tiotropium, which crystallized in the orthosteric site of the M_1_ receptor [37]. The two Asp residues play a crucial role in muscarinic ligand binding, as well as receptor activation through the recognition of the agonist’s cationic head. The M_1_ and M_2_ receptor activation by bitopic ligands **8-Cn** and **9-Cn** is primarily regulated by their Iperoxo moiety [14,38], which occupies the orthosteric binding site and adopts the same orientation as Iperoxo alone [36]. The length of the polymethylene linker connecting Iperoxo with the allosteric moiety controls the position of the latter within the allosteric vestibule and the degree of conformational receptor movements, thus affecting the extent of the ligand binding closure, which is necessary for receptor activation.

The bell-shaped responses observed for the charged methylated set of new bitopic ligands, **13-Cn** at the M_1_ and M_4_ mAChRs should be similarly modulated by the ligand-dependent allosteric coupling mechanism correlated with the linker length. The methyl tetrahydropyridinium positive charge anchors the compounds to the orthosteric binding site of the M_1_ and M_4_ receptors and ensures the degree of receptor activation depends on the ligand spacer length, which induces a graded conformational change/interference with the binding pocket closure. Interestingly, a parallel trend was observed for derivatives of **13-Cn**, with hybrid **13-C7** causing the most relevant FRET receptor activation at both M_1_ and M_4_ mAChRs. This group of derivatives essentially retains the pharmacological profile of Xanomeline at the two target receptor subtypes. 

Conversely, the tertiary amine derivatives of **12-Cn** showed a linear receptor activation pattern at M_1_ mAChRs. These hybrids lack the contribution imparted to their permanently charged analogs by a strong ionic interaction with the Asp residue in the orthosteric binding pocket. Indeed, we calculated a degree of protonation of ~15% at physiological pH for these compounds according to the p*K*_a_ values measured experimentally (p*K*_a_ ≃ 6.6–6.8). A weaker interaction at this recognition site implies for compounds **12-Cn** less stringent geometric features in orienting the allosteric fragment, with an almost negligible weight of their linker length and comparable conformational variations. However, due to the combined orthosteric/allosteric effect, also for these tertiary amines, an activating response of our model M_1_ FRET receptor was evidenced, at variance with Xanomeline alone, which was unresponsive in the same experimental conditions.

## 3. Experimental Section

### 3.1. Chemistry

#### 3.1.1. General Materials and Methods

All reagents and solvents were purchased from Sigma-Aldrich Srl (Milan, Italy) and were used without further purification. All reactions were carried out under an inert atmosphere of argon or nitrogen and monitored by thin-layer chromatography (TLC) on commercial aluminum plates precoated with silica gel 60 (F-254, Merck, Rahway, NJ, USA) or with aluminum oxide (F-254, Merck, Rahway, NJ, USA). Visualization was performed with UV light at 254 nm. Spots were further evidenced by spraying with a dilute alkaline potassium permanganate solution or a phosphomolybdic acid solution and, for tertiary amines, with the Dragendorff reagent. Glassware was oven-dried or flame-dried prior to use. The synthesized compounds were purified on glass flash chromatography columns packed with silica gel (230–400 mesh particle size, pore size 60 Å, Merck). Melting points of solid products were measured in capillary tubes with a model B 540 Büchi apparatus and were uncorrected. ^1^H NMR and ^13^C NMR spectra were recorded with a Varian Mercury 300 (^1^H, 300.063; ^13^C, 75.451 MHz) spectrometer at 20 °C. Abbreviations used for peak multiplicities are given as follows: s (singlet), bs (broad singlet), d (doublet), t (triplet), q (quartet), and m (multiplet). Coupling constants (*J*) are given in Hz, chemical shifts (*δ*) are expressed in parts per million (ppm), and they are calibrated for ^1^H using TMS and for ^13^C using residual deuterated solvent as an internal standard (CDCl_3_, 77.16 ppm; CD_3_OD, 49.00 ppm; DMSO-*d*_6_, 39.52 ppm). High-resolution mass spectra (HRMS) were recorded on a FT-ICR mass spectrometer (Bruker Apex II, 4.7 T) using electrospray ionization (ESI). Data are reported as mass-to-charge ratios (*m/z*) for the target compounds **12-Cn** and **13-Cn**. (See Supplementary Materials for ^1^H NMR, ^13^C NMR, and HRMS spectra). Hybrid compound **9-C9** was prepared according to a published procedure [35]. 

#### 3.1.2. Preparation of Xanomeline (**10**)

*2-hydroxy-2-(pyridin-3-yl)acetonitrile* (**15**). A water solution of 3-pyridinecarboxaldheyde **14** (3.00 g, 28.01 mmol) and acetic acid (1.68 g, 28.01 mmol) were added to a water solution of TMSCN (3.71 g, 37.21 mmol) at 0 °C. The reaction proceeded at room temperature for 23 h and then was extracted with ethyl acetate (3 × 20 mL). The pooled organic phases were dried over anhydrous Na_2_SO_4_ and the solvent was evaporated under reduced pressure, giving the desired product 15 as a yellow-orange oil (3.36 g, 78%): *R_f_* = 0.44 (ethyl acetate). ^1^H NMR (300 MHz, CDCl_3_): *δ* 8.73 (dt, *J* = 2.3, 0.7, 0.6 Hz, 1H; H2), 8.59 (dd, *J* = 5.0, 1.5 Hz, 1H; H6), 7.98 (dddd, *J* = 7.9, 3.8, 0.6, 0.4 Hz, 1H; H4), 7.46 (ddd, *J* = 7.9, 5.0, 0.8 Hz, 1H; H5), 5.64 (s, 1H; CH−CN). ^13^C NMR (75 MHz, CDCl_3_): *δ* 149.70, 147.10, 135.58, 133.08, 124.50, 118.81, 61.05. 

*2-Amino-2-(pyridin-3-yl) acetonitrile* (**16**). A water solution of **15** (2.65 g, 19.76 mmol) was added dropwise at room temperature to a solution of NH_4_Cl (7.93 g, 148.17 mmol) in water (25 mL) and 33% aqueous ammonia (4 mL). The reaction was kept at room temperature for 22 h. The aqueous phase was extracted four times with 10 mL of dichloromethane, and four times with 10 mL of a mixture of dichloromethane/*i*sopropanol (7:3). The pooled organic phases were dried over anhydrous Na_2_SO_4_ and concentrated under reduced pressure, giving the desired product **16** as an orange oil (1.160 g, 44%): *R_f_* = 0.52 (dichloromethane/methanol 9:1). ^1^H NMR (300 MHz, CDCl_3_): *δ* 8.80 (d, *J* = 2.3 Hz, 1H; H2), 8.64 (dd, *J* = 4.8, 1.4 Hz, 1H; H6), 7.89 (dt, *J* = 8.0, 1.7 Hz, 1H; H4), 7.37 (dd, *J* = 8.0, 4.8 Hz, 1H; H5), 4.98 (s, 1H; CH−CN), 2.01 (s, 2H; NH_2_). ^13^C NMR (75 MHz, CDCl_3_): *δ* 150.38, 148.34, 134.47, 132.19, 123.80, 120.12, 45.30.

*3-Chloro-4-(pyridin-3-yl)-1,2,5-thiadiazole* (**17**). A solution of **16** (1.15 g, 8.64 mmol) in 5 mL of dimethylformamide and S_2_Cl_2_ (1.38 mL, 17.27 mmol) was added dropwise to 10 mL of dimethylformamide at 0 °C. After 30 min, 20 mL of water was poured, and the suspension was filtered. The filtrate was basified with 9 M NaOH (10 mL), and the aqueous phase was extracted with DCM (3 × 20 mL). The pooled organic phases were dried over anhydrous Na_2_SO_4_, concentrated under reduced pressure, affording the desired product **17** as a brown solid (1.35 g, 79%): *R_f_* = 0.30 (cyclohexane/ethyl acetate 3:2). mp = 51–53 °C. ^1^H NMR (300 MHz, CDCl_3_): *δ* 9.15 (d, *J* = 2.1 Hz, 1H; H2), 8.66 (dd, *J* = 4.8, 1.5 Hz, 1H; H6), 8.21 (dt, *J* = 8.1, 1.7 Hz, 1H; H4), 7.39 (dd, *J* = 7.9, 4.8 Hz, 1H; H5). ^13^C NMR (75 MHz, CDCl_3_): *δ* 155.12, 150.85, 149.24, 143.46, 135.70, 126.84, 123.34.

*3-(Hexyloxy)-4-(pyridin-3-yl)-1,2,5-thiadiazole* (**18**). A suspension of 60% NaH (1,48 g, 61.47 mmol) in anhydrous tetrahydrofuran (3 mL) was added dropwise to a solution of 1-hexanol (2.09 g, 20.49 mmol) in anhydrous tetrahydrofuran (6 mL) at 0 °C. The suspension was kept under stirring at room temperature for 2 h. A solution of **17** (1.35 g, 6.83 mmol) in anhydrous tetrahydrofuran (5 mL) was added dropwise to the suspension. The reaction was kept under reflux for 3 h and then quenched with 10 mL of a saturated aqueous solution of NaHCO_3_. The aqueous phase was extracted with dichloromethane (3 × 10 mL). The pooled organic phases were dried over anhydrous Na_2_SO_4_ and then concentrated under reduced pressure. The crude was purified through a silica gel column chromatography, using as eluent cyclohexane/ethyl acetate 9:1. The desired product **18** was obtained as a white solid (1.53 g, 85%): *R_f_* = 0.41 (cyclohexane/ethyl acetate 4:1). mp = 49–51 °C. ^1^H NMR (300 MHz, CDCl_3_): *δ* 9.41 (dd, *J* = 2.2, 0.7 Hz, 1H; H2), 8.66 (dd, *J* = 4.8, 1.6 Hz, 1H; H6), 8.46 (dt, *J* = 8.1, 1.9 Hz, 1H; H4), 7.42 (ddd, *J* = 8.1, 4.9, 0.7 Hz, 1H; H5), 4.53 (t, *J* = 6.7 Hz, 2H; CH_2_−O), 1.89 (quint, *J* = 6.9 Hz, 2H; C*H*_2_−CH_2_−O), 1.54–1.32 (m, 6H; CH_3_−C*H*_2_−C*H*_2_−C*H*_2_−(CH_2_)_2_−O), 0.91 (t, *J* = 5.6 Hz, 3H; C*H*_3_−(CH_2_)_5_−O). ^13^C NMR (75 MHz, CDCl_3_): *δ* 162.87, 150.13, 148.67, 144.97, 134.68, 127.69, 123.38, 71.47, 31.46, 28.90, 25.71, 22.56, 14.02.

*1-Methyl-3-(4-(pentyloxy)-1,2,5-thiadiazol-3-yl)pyridin-1-ium iodide* (**19**). Iodomethane (1.08 g, 7.59 mmol) was added dropwise to a solution of **18** (500 mg, 1.90 mmol) in 4 mL of acetone. The reaction proceeded for 26 h at room temperature, then the solution was concentrated under reduced pressure, and washed with diethyl ether (10 mL). The desired product **19** was obtained as a yellow oil (733 mg, 95%): *R_f_* = 0.30 (dichloromethane/methanol 4:1). ^1^H NMR (300 MHz, CDCl_3_): *δ* 9.61 (d, *J* = 5.4 Hz, 1H; H2), 9.46 (s, 1H; H4), 9.11 (d, *J* = 8.2 Hz, 1H; H6), 8.28 (dd, *J* = 8.3, 5.4 Hz, 1H; H5), 4.80 (s, 3H; CH_3_−N^+^), 4.58 (t, *J* = 6.9 Hz, 2H; CH_2_−O), 1.91 (quint, *J* = 6.9 Hz, 2H; C*H*_2_−CH_2_−O), 1.59–1.13 (m, 6H; CH_3_−C*H*_2_−C*H*_2_−C*H*_2_−(CH_2_)_2_−O), 0.89 (t, *J* = 6.3 Hz, 3H; C*H*_3_−(CH_2_)_5_−O). ^13^C NMR (75 MHz, CDCl_3_): *δ* 163.25, 145.85, 142.99, 142.00, 139.57, 131.58, 128.77, 72.60, 50.78, 31.46, 28.83, 25.65, 22.63, 14.10.

*3-(Hexyloxy)-4-(1-methyl-1,2,5,6-tetrahydropyridin-3-yl)-1,2,5-thiadiazole* (**10**). A solution of NaBH_4_ (273 mg, 7.20 mmol) in 3 mL of methanol was added dropwise to a solution of **19** (730 mg, 1.80 mmol) in 5 mL of methanol. The reaction was kept under stirring at room temperature for 2.5 days. After quenching with 10 mL of water, the aqueous phase was extracted with dichloromethane (3 × 10 mL). The pooled organic phases were dried over anhydrous Na_2_SO_4_ and then concentrated under reduced pressure. The crude was purified through a silica gel column chromatography, using as eluent dichloromethane/methanol 95:5. Xanomeline **10** was obtained as a yellow-orange oil (383 mg, 75%): *R_f_* = 0.30 (DCM/MeOH 95:5). ^1^H NMR (300 MHz, CDCl_3_): *δ* 7.08 (t, *J* = 3.5 Hz, 1H; H4), 4.44 (t, *J* = 6.6 Hz, 2H; CH_2_−O), 3.51 (d, *J* = 2.0 Hz, 2H; H2), 2.63 (t, *J* = 5.7 Hz, 2H; H6), 2.50 (s, 5H; CH_3_−N, 2×H5), 1.83 (quint, *J* = 6.9 Hz, 2H; C*H*_2_−CH_2_−O), 1.58–1.12 (m, 6H; CH_3_−C*H*_2_−C*H*_2_−C*H*_2_−(CH_2_)_2_−O), 0.90 (t, *J* = 6.6 Hz, 3H; C*H*_3_−(CH_2_)_5_−O). ^13^C NMR (75 MHz, CDCl_3_): *δ* 162.60, 146.66, 128.97, 128.29, 71.04, 54.85, 51.19, 45.79, 31.54, 28.87, 26.39, 25.71, 22.59, 14.05.

#### 3.1.3. Preparation of 1-(ω-bromoalkyl)-3,4-dihydroquinolin-2(1H)-one Compounds (**22-Cn**)

*1-(3-bromopropyl)-3,4-dihydroquinolin-2(1H)-one* (**22-C3**). To a suspension of NaH 60% dispersion in mineral oil (136 mg, 3.4 mmol) in anhydrous dimethylformamide (1 M) was added dropwise to a solution of 3,4-dihydro-2(1H)-quinolinone **20** (200 mg, 1.36 mmol) in anhydrous dimethylformamide (1.5 M) under an argon atmosphere at 0 °C. The reaction was stirred at room temperature for 10 min, then a solution of the dibromo derivative **21-C3** (2.74 g, 13.60 mmol) in anhydrous dimethylformamide (15 M) was added dropwise at 0 °C. After stirring for 3 h at 50 °C (TLC in cyclohexane/ethyl acetate 7:3), the mixture was quenched by addition of a saturated solution of NaHCO_3_ (5 mL). The aqueous layer was extracted with ethyl acetate (3 × 3 mL) and the collected organic phases were dried over anhydrous Na_2_SO_4_, filtered and concentrated under reduced pressure. The residue was purified by a silica gel column chromatography (cyclohexane/ethyl acetate 85:15) to provide the pure compound **22-C3** as dark yellow oil (163 mg, 45%). *R_f_* = 0.37 (cyclohexane/ethyl acetate 7:3). ^1^H NMR (300 MHz, CDCl_3_) *δ* 7.26 (td, *J* = 7.8, 1.6 Hz, 1H; H8′), 7.17 (dd, *J* = 7.3, 1.0 Hz, 1H; H6′), 7.07 (d, *J* = 8.2 Hz, 1H; H5′), 7.01 (td, *J* = 7.4, 1.1 Hz, 1H; H7′), 4.08 (t, *J* = 7.4 Hz, 2H; CH_2_−N), 3.47 (t, *J* = 6.5 Hz, 2H; Br−CH_2_), 2.89 (dd, *J* = 8.7, 6.1 Hz, 2H; H4′), 2.65 (dd, *J* = 8.7, 6.2 Hz, 2H; H3′), 2.32–2.09 (m, 2H; Br−CH_2_−C*H*_2_−CH_2_−N).^13^C NMR (75 MHz, CDCl_3_): *δ* 170.52, 139.55, 128.25, 127.75, 126.58, 123.11, 114.74, 41.37, 31.95, 30.92, 30.42, 25.67.

*1-(5-bromopentyl)-3,4-dihydroquinolin-2(1H)-one* (**22-C5**). A suspension of NaH 60% dispersion in mineral oil (489 mg, 20.38 mmol) in anhydrous dimethylformamide (1 M) was added dropwise to a solution of 3,4-dihydro-2(1H)-quinolinone **20** (1.20 g, 8.15 mmol) in anhydrous dimethylformamide (1.5 M) under an argon atmosphere at 0 °C. The reaction was stirred at room temperature for 10 min, then a solution of the dibromo derivative **21-C5** (18.75 g, 81.54 mmol) in anhydrous dimethylformamide (15 M) was added dropwise at 0 °C. After stirring for 3 h at 50 °C (TLC in cyclohexane/ethyl acetate 7:3), the mixture was quenched by addition of a saturated solution of NaHCO_3_ (20 mL). The aqueous layer was extracted with ethyl acetate (3 × 10 mL) and the collected organic phases were dried over anhydrous Na_2_SO_4_, filtered and concentrated under reduced pressure. The residue was purified by a silica gel column chromatography (cyclohexane/ethyl acetate 85:15) to provide the pure compound **22-C5** as yellow oil (2.13 g, 88%). *R_f_* = 0.38 (cyclohexane/ethyl acetate 7:3). ^1^H NMR (300 MHz, CDCl_3_) *δ* 7.24 (td, *J* = 8.1, 1.5 Hz, 1H; H8′), 7.16 (dd, *J* = 7.3, 0.8 Hz, 1H; H6′), 7.04–6.95 (m, 2H; H5′, H7′), 3.94 (t, *J* = 7.6 Hz, 2H; CH_2_−N), 3.40 (t, *J* = 6.7 Hz, 2H; Br−CH_2_), 2.88 (dd, *J* = 8.6, 6.1 Hz, 2H; H4′), 2.63 (dd, *J* = 8.6, 6.1 Hz, 2H; H3′), 1.99–1.84 (m, 2H; Br−CH_2_−C*H*_2_−(CH_2_)_3_−N), 1.76–1.61 (m, 2H; Br−(CH_2_)_3_−C*H*_2_−CH_2_−N), 1.52 (ddd, *J* = 12.6, 5.5, 3.2 Hz, 2H; Br−(CH_2_)_2_−C*H*_2_−(CH_2_)_2_−N). ^13^C NMR (75 MHz, CDCl_3_): *δ* 170.25, 139.58, 128.17, 127.55, 126.75, 122.85, 114.83, 41.85, 33.69, 32.43, 32.04, 26.45, 25.70, 25.55.

*1-(7-bromoheptyl)-3,4-dihydroquinolin-2(1H)-one* (**22-C7**). A suspension of NaH 60% dispersion in mineral oil (489 mg, 20.38 mmol) in anhydrous dimethylformamide (1 M) was added dropwise to a solution of 3,4-dihydro-2(1H)-quinolinone **20** (1.20 g, 8.15 mmol) in anhydrous dimethylformamide (1.5 M) under an argon atmosphere at 0 °C. The reaction was stirred at room temperature for 10 min, then a solution of the dibromo derivative **21-C7** (13.59 g, 52.67 mmol) in anhydrous dimethylformamide (15 M) was added dropwise at 0 °C. After stirring for 3 h at 50 °C (TLC in cyclohexane/ethyl acetate 7:3), the mixture was quenched by the addition of a saturated solution of NaHCO_3_ (20 mL). The aqueous layer was extracted with ethyl acetate (3 × 10 mL) and the collected organic phases were dried over anhydrous Na_2_SO_4_, filtered and concentrated under reduced pressure. The residue was purified by a silica gel column chromatography (cyclohexane/ethyl acetate 85:15) to provide the pure compound **22-C7** as yellow oil (2.05 g, 78%). *R_f_* = 0.38 (cyclohexane/ethyl acetate 7:3). ^1^H NMR (300 MHz, CDCl_3_): *δ* 7.24 (td, J = 7.6, 1.1 Hz, 1H; H8′), 7.16 (dd, *J* = 7.7, 0.6 Hz, 1H; H6′), 7.00 (t, *J* = 7.7 Hz, 2H; H5′, H7′), 3.98–3.86 (m, 2H; CH_2_−N), 3.40 (t, *J* = 6.8 Hz, 2H; Br−CH_2_), 2.93–2.84 (m, 2H; H4′), 2.64 (dd, *J* = 8.7, 6.0 Hz, 2H; H3′), 1.91–1.79 (m, 2H; Br−CH_2_−C*H*_2_−(CH_2_)_5_−N), 1.71–1.59 (m, 2H; Br−(CH_2_)_5_−C*H*_2_−CH_2_−N), 1.49–1.32 (m, 6H; Br−(CH_2_)_2_−C*H*_2_−C*H*_2_−C*H*_2_−(CH_2_)_2_−N). ^13^C NMR (75 MHz, CDCl_3_): *δ* 170.24, 139.70, 128.14, 127.53, 126.74, 122.78, 114.91, 42.16, 34.05, 32.81, 32.07, 28.57, 28.19, 27.21, 26.83, 25.73.

*1-(9-bromononyl)-3,4-dihydroquinolin-2(1H)-one* (**22-C9**). A suspension of NaH 60% dispersion in mineral oil (285 mg, 11.89 mmol) in anhydrous dimethylformamide (1 M) was added dropwise to a solution of 3,4-dihydro-2(1H)-quinolinone **20** (700 mg, 4.76 mmol) in anhydrous dimethylformamide (1.5 M) under an argon atmosphere at 0 °C. The reaction was stirred at room temperature for 10 min, then a solution of the dibromo derivative **21-C9** (13.61 g, 47.56 mmol) in anhydrous dimethylformamide (15 M) was added dropwise at 0 °C. After stirring for 3 h at 50 °C (TLC in cyclohexane/ethyl acetate 7:3), the mixture was quenched by the addition of a saturated solution of NaHCO_3_ (10 mL). The aqueous layer was extracted with ethyl acetate (3 × 5 mL) and the collected organic phases were dried over anhydrous Na_2_SO_4_, filtered and concentrated under reduced pressure. The residue was purified by a silica gel column chromatography (cyclohexane/ethyl acetate 85:15) to provide the pure compound **22-C9** as yellow oil (1.20 g, 71%). *R_f_* = 0.37 (cyclohexane/ethyl acetate 8:2). ^1^H NMR (300 MHz, CDCl_3_): *δ* 7.24 (td, *J* = 8.2, 0.9 Hz, 1H; H8′), 7.16 (dd, *J* = 6.8, 0.8 Hz, 1H; H6′), 6.99 (t, *J* = 7.1 Hz, 2H; H5′, H7′), 3.92 (t, *J* = 7.7 Hz, 2H; CH_2_−N), 3.40 (t, *J* = 6.9 Hz, 2H; Br−CH_2_), 2.88 (dd, *J* = 8.5, 6.1 Hz, 2H; H4′), 2.63 (dd, *J* = 8.7, 6.0 Hz, 2H; H3′), 1.84 (quin, *J* = 7.0 Hz, 2H; Br−CH_2_−C*H*_2_−(CH_2_)_7_−N), 1.70–1.56 (m, 2H; Br−(CH_2_)_7_−C*H*_2_−CH_2_−N), 1.45–1.26 (m, 10H; Br−(CH_2_)_2_−C*H*_2_−C*H*_2_−C*H*_2_−C*H*_2_−C*H*_2_−(CH_2_)_2_−N). ^13^C NMR (75 MHz, CDCl_3_): *δ* 170.22, 139.78, 128.12, 127.52, 126.77, 122.75, 114.95, 42.26, 34.13, 32.94, 32.10, 29.46, 29.34, 28.80, 28.27, 27.32, 26.99, 25.76.

#### 3.1.4. Preparation of Pyridinium Bromide Salts (**23-Cn**) 

*3-(4-(hexyloxy)-1,2,5-thiadiazol-3-yl)-1-(3-(2-oxo-3,4-dihydroquinolin-1(2H)-yl)propyl)pyridin-1-ium bromide* (**23-C3**). A solution of **18** (144 mg, 0.547 mmol) in acetonitrile (0.2 M) was added dropwise to a solution of the bromide intermediate **22-C3** (293 mg, 1.09 mmol) in acetonitrile (0.2 M). The resulting reaction mixture was stirred under reflux for 2 days (TLC in cyclohexane/ethyl acetate 8:2) and then was concentrated under reduced pressure. After purification of the residue by silica gel column chromatography (dichloromethane/methanol 95:5 to 8:2), **23-C3** (225 mg, 77%) was obtained as yellow oil. *R_f_* = 0.48 (dichloromethane/methanol 8:2). ^1^H NMR (300 MHz, CD_3_OD): *δ* 9.70 (s, 1H; H2), 9.29–9.17 (m, 2H; H4, H6), 8.28 (dd, *J* = 8.2, 6.1 Hz, 1H; H5), 7.33–7.24 (m, 2H; H8′, H6′), 7.20 (d, *J* = 7.4 Hz, 1H; H5′), 7.02 (ddd, *J* = 7.5, 5.4, 3.1 Hz, 1H; H7′), 4.93 (t, *J* = 7.2 Hz, 2H; CH_2_−N^+^), 4.62 (t, *J* = 6.8 Hz, 2H; CH_2_−O), 4.14 (t, *J* = 6.8 Hz, 2H; CH_2_−N), 2.91 (dd, *J* = 8.5, 6.2 Hz, 2H; H4′), 2.61 (dd, *J* = 8.5, 6.2 Hz, 2H; H3′), 2.49 (p, *J* = 7.0 Hz, 2H; N−CH_2_−C*H*_2_−CH_2_−N^+^), 1.94 (quin, *J* = 7.1 Hz, 2H; C*H*_2_−CH_2_−O), 1.56–1.31 (m, 6H; CH_3_−C*H*_2_−C*H*_2_−C*H*_2_−(CH_2_)_2_−O), 0.90 (t, *J* = 7.0 Hz, 3H; C*H*_3_−(CH_2_)_5_−O). ^13^C NMR (75 MHz, CD_3_OD): *δ* 172.78, 164.42, 145.78, 144.48, 143.88, 141.84, 139.91, 133.03, 129.63, 129.23, 128.81, 128.00, 124.51, 116.22, 73.28, 61.20, 39.70, 32.62, 32.57, 30.36, 29.77, 26.63, 26.11, 23.58, 14.39.

*3-(4-(hexyloxy)-1,2,5-thiadiazol-3-yl)-1-(5-(2-oxo-3,4-dihydroquinolin-1(2H)-yl)pentyl)pyridin-1-ium bromide* (**23-C5**). A solution of **18** (270 mg, 1.03 mmol) in acetonitrile (0.2 M) was added dropwise to a solution of bromide intermediate **22-C5** (1.92 g, 7.18 mmol) in acetonitrile (0.2 M). The resulting reaction mixture was stirred under reflux for 7 days (TLC in cyclohexane/ethyl acetate 8:2) and then was concentrated under reduced pressure. After purification of the residue by a silica gel column chromatography (dichloromethane/methanol 95:5 to 8:2), **23-C5** was obtained as beige oil (570 mg, 99%). *R_f_* = 0.36 (dichloromethane/methanol 85:15). ^1^H NMR (300 MHz, CD_3_OD): *δ* 9.62 (s, 1H; H2), 9.23 (dt, *J* = 8.3, 1.4, 1.4 Hz, 1H; H4), 9.11 (dt, *J* = 6.1, 1.2, 1.2 Hz, 1H; H6), 8.27 (dd, *J* = 8.2, 6.1 Hz, 1H; H5), 7.24 (td, *J* = 8.2, 1.6 Hz, 1H; H8′), 7.16 (dd, *J* = 11.0, 8.0, 2H; H6′, H5′), 7.00 (td, *J* = 7.3, 1.1 Hz, 1H; H7′), 4.80 (t, *J* = 7.3 Hz, 2H; CH_2_−N^+^), 4.62 (t, *J* = 6.7 Hz, 2H; CH_2_−O), 4.00 (t, *J* = 7.2 Hz, 2H; CH_2_−N), 2.84 (dd, *J* = 8.0, 6.6 Hz, 2H; H4′), 2.55 (dd, *J* = 8.6, 6.2 Hz, 2H; H3′), 2.16 (quin, *J* = 6.9 Hz, 2H; N−(CH_2_)_3_−C*H*_2_−CH_2_−N^+^), 1.95 (quin, *J* = 7.0 Hz, 2H; C*H*_2_−CH_2_−O), 1.74 (quin, *J* = 6.9 Hz, 2H; N−CH_2_−C*H*_2_−(CH_2_)_3_−N^+^), 1.55–1.28 (m, 8H; 6×CH_3_−C*H*_2_−C*H*_2_−C*H*_2_−(CH_2_)_2_−O, 2×N−(CH_2_)_2_−C*H*_2_−(CH_2_)_2_−N^+^), 0.91 (t, *J* = 7.0 Hz, 3H; C*H*_3_−(CH_2_)_5_−O). ^13^C NMR (75 MHz, CD_3_OD): *δ* 172.09, 164.30, 145.64, 144.11, 143.68, 141.83, 140.04, 132.85, 129.67, 128.99, 128.58, 127.84, 124.12, 116.28, 73.21, 63.28, 42.14, 32.68, 32.52, 31.89, 29.72, 27.54, 26.61, 26.14, 24.08, 23.54, 14.45.

*3-(4-(hexyloxy)-1,2,5-thiadiazol-3-yl)-1-(7-(2-oxo-3,4-dihydroquinolin-1(2H)-yl)heptyl)pyridin-1-ium bromide* (**23-C7**). A solution of **18** (260 mg, 0.987 mmol) in acetonitrile (0.2 M) was added dropwise to a solution of bromide intermediate **22-C7** (1.97 g, 6.08 mmol) in acetonitrile (0.2 M). The resulting reaction mixture was stirred under reflux for 7 days (TLC in cyclohexane/ethyl acetate 8:2) and then was concentrated under reduced pressure. After purification of the residue by a silica gel column chromatography (dichloromethane/methanol 95:5 to 85:15), **23-C7** was obtained as beige oil (527 mg, 91%). *R_f_* = 0.38 (dichloromethane/methanol 85:15). ^1^H NMR (300 MHz, CD_3_OD): *δ* 9.61 (s, 1H; H2), 9.24 (dt, *J* = 8.2, 1.1 Hz, 1H; H4), 9.07 (d, *J* = 6.0 Hz, 1H; H6), 8.25 (dd, *J* = 8.2, 6.1 Hz, 1H; H5), 7.23 (dd, *J* = 16.4, 8.1 Hz, 2H; H8′, H6′), 7.12 (d, *J* = 8.1 Hz, 1H; H5′), 7.02 (td, *J* = 7.5, 0.9 Hz, 1H; H7′), 4.75 (t, *J* = 7.5 Hz, 2H; CH_2_−N^+^), 4.63 (t, *J* = 6.7 Hz, 2H; CH_2_−O), 3.96 (t, *J* = 7.4 Hz, 2H; CH_2_−N), 2.88 (dd, *J* = 8.0, 6.7 Hz, 2H; H4′), 2.58 (dd, *J* = 8.5, 6.1 Hz, 2H; H3′), 2.09 (quin, *J* = 6.6 Hz, 2H; N−(CH_2_)_5_−C*H*_2_−CH_2_−N^+^), 1.95 (quin, *J* = 7.2 Hz, 2H; C*H*_2_−CH_2_−O), 1.64 (quin, *J* = 7.0 Hz, 2H; N−CH_2_−C*H*_2_−(CH_2_)_5_−N^+^), 1.57–1.22 (m, 12H; 6×CH_3_−C*H*_2_−C*H*_2_−C*H*_2_−(CH_2_)_2_−O, 6×N−(CH_2_)_2_−C*H*_2_−C*H*_2_−C*H*_2_−(CH_2_)_2_−N^+^), 0.92 (t, *J* = 6.9 Hz, 3H; C*H*_3_−(CH_2_)_5_−O).^13^C NMR (75 MHz, CD_3_OD): *δ* 172.42, 164.44, 145.56, 144.20, 143.79, 141.87, 140.23, 133.15, 129.66, 129.06, 128.58, 128.08, 124.27, 116.34, 73.30, 63.53, 42.73, 32.79, 32.64, 32.30, 29.84, 29.59, 27.98, 27.39, 26.95, 26.73, 26.20, 23.62, 14.41.

*3-(4-(hexyloxy)-1,2,5-thiadiazol-3-yl)-1-(9-(2-oxo-3,4-dihydroquinolin-1(2H)-yl)nonyl)pyridin-1-ium bromide* (**23-C9**). A solution of **18** (265 mg, 1.01 mmol) in acetonitrile (0.2 M) was added dropwise to a solution of bromide intermediate **22-C9** (2.04 g, 6.04 mmol) in acetonitrile (0.2 M). The resulting reaction mixture was stirred under reflux for 6 days (TLC in cyclohexane/ethyl acetate 8:2) and then was concentrated under reduced pressure. After purification of the residue by a silica gel column chromatography (dichloromethane/methanol 95:5 to 85:15), **23-C9** was obtained as beige oil (595 mg, 96%). *R_f_* = 0.59 (dichloromethane/methanol 85:15). ^1^H NMR (300 MHz, CD_3_OD): *δ* 9.62 (s, 1H; H2), 9.22 (dt, *J* = 8.3, 1.2 Hz, 1H; H4), 9.10 (dd, *J* = 6.1, 0.5 Hz, 1H; H6), 8.26 (dd, *J* = 8.2, 6.2 Hz, 1H; H5), 7.31–7.15 (m, 2H; H8′, H6′), 7.09 (d, *J* = 8.0 Hz, 1H; H5′), 7.01 (td, *J* = 7.4, 0.7 Hz, 1H; H7′), 4.77 (t, *J* = 7.5 Hz, 2H; CH_2_−N^+^), 4.62 (t, *J* = 6.7 Hz, 2H; CH_2_−O), 3.93 (t, *J* = 7.5 Hz, 2H; CH_2_−N), 2.86 (dd, *J* = 8.4, 6.3 Hz, 2H; H4′), 2.57 (dd, *J* = 8.9, 5.9 Hz, 2H; H3′), 2.09 (p, 2H; N−(CH_2_)_7_−C*H*_2_−CH_2_−N^+^), 1.95 (p, *J* = 7.0 Hz, 2H; C*H*_2_−CH_2_−O), 1.67–1.22 (m, 18H; 2×N−CH_2_−C*H*_2_−(CH_2_)_7_−N^+^, 6×CH_3_−C*H*_2_−C*H*_2_−C*H*_2_−(CH_2_)_2_−O, 10×N−(CH_2_)_2_−C*H*_2_−C*H*_2_−C*H*_2_−C*H*_2_−C*H*_2_−(CH_2_)_2_−N^+^), 0.92 (t, *J* = 7.0 Hz, 3H; C*H*_3_−(CH_2_)_5_−O). ^13^C NMR (75 MHz, CD_3_OD): *δ* 171.51, 164.16, 145.77, 143.94, 143.64, 141.81, 140.10, 132.57, 129.69, 128.97, 128.51, 127.67, 123.95, 116.07, 73.15, 63.36, 42.70, 32.71, 32.52, 30.20, 30.07, 29.91, 29.70, 28.00, 27.61, 26.96, 26.62, 26.15, 23.55, 14.66.

#### 3.1.5. Preparation of Xanomeline/77-LH-28-1 Hybrids (**12-Cn**) 

*1-(3-(5-(4-(hexyloxy)-1,2,5-thiadiazol-3-yl)-3,6-dihydropyridin-1(2H)-yl)propyl)-3,4-dihydroquinolin-2(1H)-one* (**12-C3**). A solution of the pyridinium salt intermediate **23-C3** (212 mg, 0.399 mmol) in ethanol (0.1 M) and a solution of NaBH_4_ (30 mg, 0.798 mmol) in ethanol (0.5 M) was added dropwise at 0 °C. The reaction mixture was allowed to stir at room temperature for 2 h (TLC in dichloromethane/methanol 9:1). Then, a saturated solution of NaHCO_3_ (5 mL) was added and the aqueous layer was extracted with dichloromethane (3 × 5 mL). The collected organic phases were dried over anhydrous Na_2_SO_4_, filtered and concentrated under reduced pressure. After purification of the residue by a silica gel column chromatography (dichloromethane/methanol 95:5), **12-C3** was obtained as yellow oil (94 mg, 52%). *R_f_* = 0.52 (dichloromethane/methanol 96:4). ^1^H NMR (300 MHz, CDCl_3_): *δ* 7.18 (td, *J* = 7.8, 1.3 Hz, 1H; H8′), 7.14–7.01 (m, 3H; H6′, H5′, H7′), 6.95 (td, *J* = 7.3, 0.9 Hz, 1H; H4), 4.41 (t, *J* = 6.6 Hz, 2H; CH_2_−O), 4.01 (t, *J* = 7.4 Hz, 2H; CH_2_−N_quin_), 3.48 (d, *J* = 1.8 Hz, 2H; H2), 2.86 (dd, *J* = 8.5, 6.1 Hz, 2H; H4′), 2.67–2.47 (m, 4H; H6, H3′), 2.47–2.34 (m, *J* = 16.6 Hz, 2H; CH_2_−N_xano_), 1.94 (quin, *J* = 7.3 Hz, 2H; H5), 1.81 (quin, *J* = 6.9 Hz, 2H; C*H*_2_−CH_2_−O), 1.51–1.21 (m, 8H; 6×CH_3_−C*H*_2_−C*H*_2_−C*H*_2_−(CH_2_)_2_−O; 2×N_quin_−CH_2_−C*H*_2_−CH_2_−N_xano_), 0.89 (t, *J* = 7.0 Hz, 3H; C*H*_3_−(CH_2_)_5_−O). ^13^C NMR (75 MHz, CDCl_3_): *δ* 170.21, 162.60, 146.92, 139.69, 129.37, 128.79, 128.01, 127.49, 126.55, 122.73, 114.98, 71.02, 55.54, 53.44, 49.39, 40.54, 32.00, 31.46, 28.89, 26.58, 25.71, 24.99, 22.59, 14.05. 

*5-(4-(hexyloxy)-1,2,5-thiadiazol-3-yl)-1-(3-(2-oxo-3,4-dihydroquinolin-1(2H)-yl)propyl)-1,2,3,6-tetrahydropyridin-1-ium carboxyformate* (**12-C3 × Oxalate**). Oxalic acid (23 mg, 0.251 mmol) was added to a solution of free base **12-C3** (57 mg, 0.125 mmol) in MeOH (1.3 M). After the reaction mixture was stirred at 40 °C for 48 h (dichloromethane/methanol 9:1), the solvent was removed under reduced pressure and the crude salt was recrystallized from *i*PrOH/(*i*Pr)_2_O to provide the pure oxalate salt **12-C3 × C_2_H_2_O_4_** as white off solid (45 mg, 79%). mp = 140–142 °C. ^1^H NMR (300 MHz, (CD_3_)_2_SO): *δ* 7.32–7.14 (m, 3H; H8′, H6′, H5′), 7.10 (s, 1H; H7′), 7.00 (t, *J* = 6.7 Hz, 1H; H4), 4.44 (t, *J* = 6.5 Hz, 2H; CH_2_−O), 4.04–3.82 (m, 4H; 2×CH_2_−N_quin_, 2×H2), 3.08 (m, 4H; 2×H6, 2×CH_2_−N^+^_xano_), 2.87 (dd, *J* = 7.7, 6.7 Hz, 2H; H4′), 2.61–2.45 (m, 4H; 2×H3′, H5), 1.95 (s, 2H; C*H*_2_−CH_2_−O), 1.79 (quin, *J* = 6.9 Hz, 2H; N_quin_−CH_2_−C*H*_2_−CH_2_−N^+^_xano_), 1.51–1.19 (m, 6H; CH_3_−C*H*_2_−C*H*_2_−C*H*_2_−(CH_2_)_2_−O), 0.87 (t, *J* = 6.8 Hz, 3H; C*H*_3_−(CH_2_)_5_−O). ^13^C NMR (75 MHz, (CD_3_)_2_SO): *δ* 169.45, 163.49 (2C), 162.07, 145.17, 139.02, 128.23, 127.94, 127.32, 126.43, 125.40, 122.47, 114.73, 71.05, 53.37, 50.41, 47.57, 31.28, 30.78, 28.11, 25.02, 24.69, 23.44, 22.80, 22.63, 21.94, 13.82. Anal. calcd for C_27_H_36_N_4_O_6_S: C, 59.54; H, 6.66; N, 10.29; found: C, 58,23; H, 6,57; N, 9.88. HRMS (ESI) *m/z* calcd for C_25_H_35_N_4_O_2_S_1_ 455.24752 [M + H]^+^, found 455.24786.

*1-(5-(5-(4-(hexyloxy)-1,2,5-thiadiazol-3-yl)-3,6-dihydropyridin-1(2H)-yl)pentyl)-3,4-dihydroquinolin-2(1H)-one* (**12-C5**). A solution of the pyridinium salt intermediate **23-C5** (594 mg, 1.06 mmol) in ethanol (0.1 M) and a solution of NaBH_4_ (80 mg, 2.12 mmol) in ethanol (0.5 M) was added dropwise at 0 °C. The reaction mixture was allowed to stir at room temperature for 12 h (TLC in dichloromethane/methanol 9:1). Then, a saturated solution of NaHCO_3_ (10 mL) was added and the aqueous layer was extracted with dichloromethane (3 × 10 mL). The collected organic phases were dried over anhydrous Na_2_SO_4_, filtered and concentrated under reduced pressure. After purification of the residue by a silica gel column chromatography (dichloromethane/methanol 98:2 to 96:4), **12-C5** was obtained as orange oil (240 mg, 50%). *R_f_* = 0.46 (dichloromethane/methanol 96:4). ^1^H NMR (300 MHz, CD_3_OD): *δ* 7.29–7.07 (m, 4H; H8′, H6′, H5′, H7′), 7.00 (td, *J* = 7.4, 0.9 Hz, 1H; H4), 4.46 (t, *J* = 6.5 Hz, 2H; CH_2_−O), 4.03–3.91 (m, 2H; CH_2_−N_quin_), 3.51 (d, *J* = 1.7 Hz, 2H; H2), 2.93–2.83 (m, 2H; H4′), 2.67 (t, *J* = 5.8 Hz, 2H; C*H*_2_−CH_2_−N_quin_), 2.63–2.50 (m, 4H; H6, H3′), 2.43 (d, *J* = 3.7 Hz, 2H; CH_2_−N_xano_), 1.83 (quin, *J* = 7.1 Hz, 2H; H5), 1.74–1.58 (m, 4H; 2×C*H*_2_−CH_2_−O, 2×C*H*_2_−CH_2_−N_xano_), 1.50–1.29 (m, 8H; 6×CH_3_−C*H*_2_−C*H*_2_−C*H*_2_−(CH_2_)_2_−O; 2×N_quin_−(CH_2_)_2_−C*H*_2_−(CH_2_)_2_−N_xano_), 0.91 (t, *J* = 7.0 Hz, 3H; C*H*_3_−(CH_2_)_5_−O). ^13^C NMR (75 MHz, CD_3_OD): *δ* 172.16, 163.71, 147.72, 140.28, 129.84, 129.77, 129.04, 128.55, 128.00, 124.15, 116.25, 72.15, 59.13, 54.04, 50.19, 42.81, 32.77, 32.54, 29.87, 28.08, 27.17, 26.79 (2C), 26.25, 25.71, 23.59, 14.46. HRMS (ESI) *m*/*z* calcd for C_27_H_39_N_4_O_2_S_1_ 483.27882 [M + H]^+^, found 483.27922.

*1-(7-(5-(4-(hexyloxy)-1,2,5-thiadiazol-3-yl)-3,6-dihydropyridin-1(2H)-yl)heptyl)-3,4-dihydroquinolin-2(1H)-one* (**12-C7**). A solution of the pyridinium salt intermediate **23-C7** (510 mg, 0.868 mmol) in ethanol (0.1 M) and a solution of NaBH_4_ (82 mg, 2.17 mmol) in ethanol (0.5 M) was added dropwise at 0 °C. The reaction mixture was allowed to stir at room temperature for 22 h (TLC in dichloromethane/methanol 9:1). Then, a saturated solution of NaHCO_3_ (10 mL) was added and the aqueous layer was extracted with dichloromethane (3 × 10 mL). The collected organic phases were dried over anhydrous Na_2_SO_4_, filtered and concentrated under reduced pressure. After purification of the residue by a silica gel column chromatography (dichloromethane/methanol 97:3), **12-C7** was obtained as orange oil (133.43 mg, 30%). *R_f_* = 0.35 (dichloromethane/methanol 95:5). ^1^H NMR (300 MHz, CD_3_OD): *δ* 7.24 (td, *J* = 7.8, 1.4 Hz, 1H; H8′), 7.20 (dd, *J* = 7.3, 0.8 Hz, 1H; H6′), 7.15–7.07 (m, 2H; H5′, H7′), 7.01 (td, *J* = 7.4, 1.0 Hz, 1H; H4), 4.47 (t, *J* = 6.5 Hz, 2H; CH_2_−O), 3.96 (t, *J* = 7.5 Hz, 2H; CH_2_−N_quin_), 3.54 (d, *J* = 1.9 Hz, 2H; H2), 2.87 (dd, *J* = 8.4, 6.3 Hz, 2H; H4′), 2.70 (t, *J* = 5.8 Hz, 2H; CH_2_−CH_2_−N_quin_), 2.63–2.51 (m, 4H; H6, H3′), 2.45 (d, *J* = 3.9 Hz, 2H; CH_2_−N_xano_), 1.84 (quin, *J* = 6.6 Hz, 2H; H5), 1.69–1.55 (m, 4H; 2×C*H*_2_−CH_2_−O, 2×C*H*_2_−CH_2_−N_xano_), 1.58–1.22 (m, 12H; 6×CH_3_−C*H*_2_−C*H*_2_−C*H*_2_−(CH_2_)_2_−O; 6×N_quin_−(CH_2_)_2_−C*H*_2_−C*H*_2_−C*H*_2_−(CH_2_)_2_−N_xano_), 0.92 (t, *J* = 7.0 Hz, 3H; C*H*_3_−(CH_2_)_5_−O).^13^C NMR (75 MHz, CD_3_OD): *δ* 172.52, 163.81, 147.44, 140.31, 129.60, 129.08, 129.05, 128.58, 128.17, 124.29, 116.39, 72.26, 59.07, 53.56, 50.12, 42.88, 32.82, 32.56, 30.12, 29.89, 28.34, 28.12, 27.63, 26.99, 26.81, 26.24, 26.20, 23.60, 14.36. HRMS (ESI) *m*/*z* calcd for C_29_H_43_N_4_O_2_S_1_ 511.31012 [M + H]^+^, found 511.31064.

*1-(9-(5-(4-(hexyloxy)-1,2,5-thiadiazol-3-yl)-3,6-dihydropyridin-1(2H)-yl)nonyl)-3,4-dihydroquinolin-2(1H)-one* (**12-C9**). A solution of the pyridinium salt intermediate **23-C9** (600 mg, 0.975 mmol) in ethanol (0.1 M) and a solution of NaBH_4_ (74 mg, 1.95 mmol) in ethanol (0.5 M) was added dropwise at 0 °C. The reaction mixture was allowed to stir at room temperature for 12 h (TLC in dichloromethane/methanol 9:1). Then, a saturated solution of NaHCO_3_ (10 mL) was added and the aqueous layer was extracted with dichloromethane (3 × 10 mL). The collected organic phases were dried over anhydrous Na_2_SO_4_, filtered and concentrated under reduced pressure. After purification of the residue by a silica gel column chromatography (dichloromethane/methanol 99:1 to 96:4), **12-C9** was obtained as orange oil (140 mg, 27%). *R_f_* = 0.35 (dichloromethane/methanol 94:6). ^1^H NMR (300 MHz, CD_3_OD): *δ* 7.29–7.16 (m, 2H, H8′, H6′), 7.13–7.06 (m, 2H; H5′; H7′), 7.01 (td, *J* = 7.4, 1.4 Hz, 1H; H4), 4.45 (t, *J* = 6.5 Hz, 2H; CH_2_−O), 3.94 (t, *J* = 7.5 Hz, 2H; CH_2_−N_quin_), 3.52 (d, *J* = 1.8 Hz, 2H; H2), 2.86 (dd, *J* = 8.0, 6.7 Hz, 2H; H4′), 2.68 (t, *J* = 5.8 Hz, 2H; C*H*_2_−CH_2_−N_quin_), 2.63–2.49 (m, 4H; H6, H3′), 2.44 (d, *J* = 3.7 Hz, 2H; CH_2_−N_xano_), 1.82 (quin, *J* = 7.2 Hz, 2H; H5), 1.69–1.53 (m, 4H; 2×C*H*_2_−CH_2_−O, 2×C*H*_2_−CH_2_−N_xano_), 1.52–1.30 (m, 16H; 6×CH_3_−C*H*_2_−C*H*_2_−C*H*_2_−(CH_2_)_2_−O; 10×N_quin_−(CH_2_)_2_−C*H*_2_−C*H*_2_−C*H*_2_−C*H*_2_−C*H*_2_−(CH_2_)_2_−N_xano_), 0.91 (t, *J* = 7.0 Hz, 3H; C*H*_3_−(CH_2_)_5_−O). ^13^C NMR (75 MHz, CD_3_OD): *δ* 172.50, 163.82, 147.78, 140.33, 129.80 (2C), 129.08, 128.57, 128.17, 124.29, 116.38, 72.20, 59.46, 54.01, 50.32, 42.95, 32.83, 32.58, 30.51, 30.48, 30.27, 29.91, 28.61, 28.17, 27.73, 27.50, 26.84, 26.70, 26.25, 23.62, 14.37. HRMS (ESI) *m*/*z* calcd for C_31_H_47_N_4_O_2_S_1_ 539.34142 [M + H]^+^, found 539.34243.

#### 3.1.6. Preparation of Quaternary Xanomeline/77-LH-28-1 Bromide Salt Hybrids (**13-Cn**)

*5-(4-(hexyloxy)-1,2,5-thiadiazol-3-yl)-1-methyl-1-(3-(2-oxo-3,4-dihydroquinolin-1(2H)-yl)propyl)-1,2,3,6-tetrahydropyridin-1-ium bromide* (**13-C3**). A solution of Xanomeline **10** (111 mg, 0.394 mmol) in acetonitrile (0.1 M) was added dropwise to a solution of the bromide intermediate **22-C3** (106 mg, 0.394 mmol) in acetonitrile (0.1 M). The resulting reaction mixture was stirred under reflux for 12 h (TLC in dichloromethane/methanol 9:1) and then was concentrated under reduced pressure. After purification of the residue by a silica gel column chromatography (dichloromethane/methanol 9:1 to 8:2), **13-C3** was obtained as brown-yellow oil (80 mg, 37%). *R_f_* = 0.36 (dichloromethane/methanol 9:1). ^1^H NMR (300 MHz, MeOD): *δ* 7.32–7.17 (m, 4H; H8′, H6′, H5′, H7′), 7.03 (td, *J* = 7.3, 1.6 Hz, 1H; H4), 4.57–4.40 (m, 4H; CH_2_−N, CH_2_−O), 4.12 (dd, *J* = 11.3, 6.8 Hz, 2H; H2), 3.67 (dd, *J* = 9.4, 6.1 Hz, 2H; H6), 3.59 (dd, *J* = 10.5, 6.4 Hz, 2H; CH_2_−N^+^), 3.21 (s, 3H; CH_3_−N^+^), 2.92 (dd, *J* = 16.8, 10.1 Hz, 2H; H4′), 2.77 (s, 2H; H5), 2.62 (dd, *J* = 9.0, 5.9 Hz, 2H; H3′), 2.25 (dd, *J* = 12.5, 6.4 Hz, 2H; N−CH_2_−C*H*_2_−CH_2_−N^+^), 1.88 (p, *J* = 7.0 Hz, 2H; C*H*_2_−CH_2_−O), 1.57–1.30 (m, 6H; CH_3_−C*H*_2_−C*H*_2_−C*H*_2_−(CH_2_)_2_−O), 0.94 (t, *J* = 7.0 Hz, 3H; C*H*_3_−(CH_2_)_5_−O).^13^C NMR (75 MHz, CD_3_OD): *δ* 172.96, 163.88, 145.29, 139.70, 129.23, 128.82, 128.10, 127.52, 124.64, 124.24, 116.31, 72.63, 61.96, 60.06, 57.57, 39.75, 32.67, 32.58, 29.86, 26.76 (2C), 26.12, 23.59, 22.55, 14.38 (2C). HRMS (ESI) *m*/*z* calcd for C_26_H_37_N_4_O_2_S_1_ 469.26317 [M + H]^+^, found 469.26253.

*5-(4-(hexyloxy)-1,2,5-thiadiazol-3-yl)-1-methyl-1-(5-(2-oxo-3,4-dihydroquinolin-1(2H)-yl)pentyl)-1,2,3,6-tetrahydropyridin-1-ium bromide* (**13-C5**). A solution of Xanomeline **10** (265 mg, 0.942 mmol) in acetonitrile (0.1 M) was added dropwise to a solution of the bromide intermediate **22-C5** (335 mg, 1.131 mmol) in acetonitrile (0.1 M). The resulting reaction mixture was stirred under reflux for 22 h (TLC in dichloromethane/methanol 9:1) and then was concentrated under reduced pressure. After purification of the residue by a silica gel column chromatography (dichloromethane/methanol 9:1), **13-C5** was obtained as brown-yellow oil (174 mg, 32%). *R_f_* = 0.52 (dichloromethane/methanol 9:1). ^1^H NMR (300 MHz, CDCl_3_): *δ* 7.14 (dt, J = 7.7, 1.6 Hz, 2H; H8′, H6′), 7.04 (dd, *J* = 7.3, 1.0 Hz, 1H; H4), 6.88 (dt, *J* = 8.1, 1.4 Hz, 2H; H5′, H7′), 4.49 (s, *J* = 17.8 Hz, 2H; CH_2_−N), 4.34 (t, *J* = 6.7 Hz, 2H; CH_2_−O), 4.05 (dd, *J* = 12.1, 6.1 Hz, 1H; 1×H2), 3.90–3.75 (m, 3H; 1×H2, 2×H6), 3.67 (dd, *J* = 14.5, 8.0 Hz, 2H; CH_2_−N^+^), 3.34 (s, 3H; CH_3_−N^+^), 2.75 (dd, *J* = 15.8, 7.6 Hz, 4H; H4′, H5), 2.48 (dd, *J* = 8.9, 5.8 Hz, 2H; H3′), 1.92–1.56 (m, 6H; 2×N−(CH_2_)_3_−C*H*_2_−CH_2_−N^+^, 2×C*H*_2_−CH_2_−O, 2×N−CH_2_−C*H*_2_−(CH_2_)_3_−N^+^), 1.45–1.19 (m, 8H; 6×CH_3_−C*H*_2_−C*H*_2_−C*H*_2_−(CH_2_)_2_−O, 2×N−(CH_2_)_2_−C*H*_2_−(CH_2_)_2_−N^+^), 0.79 (t, *J* = 7.0 Hz, 3H; C*H*_3_−(CH_2_)_5_−O). ^13^C NMR (75 MHz, CDCl_3_): *δ* 170.14, 162.40, 143.53, 138.91, 127.88, 127.43, 126.21, 125.78, 122.85, 122.73, 114.69, 71.33, 63.07, 58.60, 56.26, 49.92, 48.13, 41.02, 31.65, 31.14, 28.53, 26.40, 25.39, 25.28, 23.21, 22.29, 21.66, 13.82. HRMS (ESI) *m*/*z* calcd for C_28_H_41_N_4_O_2_S_1_ 497.29447 [M + H]^+^, found 497.29426.

*5-(4-(hexyloxy)-1,2,5-thiadiazol-3-yl)-1-methyl-1-(7-(2-oxo-3,4-dihydroquinolin-1(2H)-yl)heptyl)-1,2,3,6-tetrahydropyridin-1-ium bromide* (**13-C7**). A solution of Xanomeline **10** (217 mg, 0.771 mmol) in acetonitrile (0.1 M) was added dropwise to a solution of bromide intermediate **22-C7** (300 mg, 0.925 mmol) in acetonitrile (0.1 M). The resulting reaction mixture was stirred under reflux for 21 h (TLC in dichloromethane/methanol 9:1) and then was concentrated under reduced pressure. After purification of the residue by a silica gel column chromatography (dichloromethane/methanol 95:5), **13-C7** was obtained as brown-yellow oil (116 mg, 24%). *R_f_* = 0.58 (dichloromethane/methanol 9:1). ^1^H NMR (300 MHz, CDCl_3_): *δ* 7.25–7.16 (m, 2H; H8′, H6′), 7.11 (d, *J* = 6.4 Hz, 1H; H4), 6.94 (t, *J* = 7.1 Hz, 2H; H5′, H7′), 4.54 (s, 2H; CH_2_−N) 4.41 (t, *J* = 6.7 Hz, 2H; CH_2_−O), 4.22 (dt, *J* = 11.8, 5.7 Hz, 1H; 1×H2), 3.96–3.80 (m, 3H; 1×H2, 2×H6), 3.78–3.57 (m, 2H; CH_2_−N^+^), 3.42 (s, 3H; CH_3_−N^+^), 2.90–2.63 (m, 4H; H4′, H5), 2.56 (dd, *J* = 8.8, 5.9 Hz, 2H; H3′), 1.86–1.75 (m, 3H; 2×C*H*_2_−CH_2_−O; 1×N−CH_2_−C*H*_2_−(CH_2_)_5_−N^+^), 1.66–1.50 (m, 3H; 1×N−CH_2_−C*H*_2_−(CH_2_)_5_−N^+^; 2×N−(CH_2_)_5_−C*H*_2_−CH_2_−N^+^), 1.44–1.24 (m, 12H; 6×CH_3_−C*H*_2_−C*H*_2_−C*H*_2_−(CH_2_)_2_−O, 6×N−(CH_2_)_2_−C*H*_2_−C*H*_2_−C*H*_2_−(CH_2_)_2_−N^+^), 0.86 (t, *J* = 7.0 Hz, 3H; C*H*_3_−(CH_2_)_5_−O). ^13^C NMR (75 MHz, CDCl_3_): *δ* 170.14, 162.61, 143.67, 139.38, 128.00, 127.52, 126.49, 125.94, 123.08, 122.74, 114.85, 77.16, 71.57, 63.37, 58.62, 56.53, 48.33, 41.79, 31.93, 31.36, 29.68, 28.75, 26.97, 26.50, 26.21, 25.60, 25.55, 22.51, 22.28, 21.90, 14.02. HRMS (ESI) *m*/*z* calcd for C_30_H_45_N_4_O_2_S_1_ 525.32577 [M + H]^+^, found 525.32626.

*5-(4-(hexyloxy)-1,2,5-thiadiazol-3-yl)-1-methyl-1-(9-(2-oxo-3,4-dihydroquinolin-1(2H)-yl)nonyl)-1,2,3,6-tetrahydropyridin-1-ium bromide* (**13-C9**). A solution of Xanomeline **10** (266 mg, 0.945 mmol) in acetonitrile (0.1 M) was added dropwise to a solution of bromide intermediate **22-C9** (499 mg, 1.42 mmol) in acetonitrile (0.1 M). The resulting reaction mixture was stirred under reflux for 27 h (TLC in dichloromethane/methanol 9:1) and then was concentrated under reduced pressure. After purification of the residue by a silica gel column chromatography (dichloromethane/methanol 95:5 to 9:1), **13-C9** was obtained as yellow solid (527 mg, 91%). *R_f_* = 0.38 (dichloromethane/methanol 85:15). mp = 56–58 °C. ^1^H NMR (300 MHz, CD_3_OD): *δ* 7.27 (ddd, *J* = 19.1, 8.4, 5.6 Hz, 3H; H8′, H6′, H4), 7.12 (d, *J* = 8.0 Hz, 1H; H5′), 7.03 (t, *J* = 7.3 Hz, 1H; H7′), 4.52 (t, *J* = 6.6 Hz, 2H; CH_2_−N), 4.47 (s, 2H; H2), 3.95 (t, *J* = 7.5 Hz, 2H; CH_2_−O), 3.69–3.55 (m, 2H; H6), 3.51–3.41 (m, 2H; CH_2_−N^+^), 3.18 (s, 3H; CH_3_−N^+^), 2.88 (t, *J* = 6.8 Hz, 2H; H4′), 2.81 (s, 2H; H5), 2.59 (dd, *J* = 8.6, 6.1 Hz, 2H; H3′), 1.98–1.79 (m, 4H; C*H*_2_−CH_2_−O, N−(CH_2_)_5_−C*H*_2_−CH_2_−N^+^), 1.62 (quin, *J* = 6.9 Hz, 2H; C*H*_2_−CH_2_−N), 1.53–1.31 (m, 16H; 6×CH_3_−C*H*_2_−C*H*_2_−C*H*_2_−(CH_2_)_2_−O, 10×N−(CH_2_)_2_−C*H*_2_−C*H*_2_−C*H*_2_−C*H*_2_−C*H*_2_−(CH_2_)_2_−N^+^), 0.93 (t, *J* = 7.0 Hz, 3H; C*H*_3_−(CH_2_)_5_−O). ^13^C NMR (75 MHz, CD_3_OD): *δ* 172.59, 163.96, 145.51, 140.30, 129.08, 128.59, 128.20, 127.63, 124.36, 124.33, 116.41, 72.65, 65.50, 60.25, 57.23, 42.88, 32.84, 32.59, 30.22, 30.09, 29.98, 29.86, 28.09, 27.63, 27.31, 26.76 (2C), 26.23, 23.60, 23.03, 22.57, 14.36. HRMS (ESI) *m*/*z* calcd for C_32_H_49_N_4_O_2_S_1_ 553.35707 [M + H]^+^, found 553.35769.

### 3.2. Biology

#### 3.2.1. Construction of the Muscarinic FRET Sensors

Muscarinic ACh receptor constructs were fused to the enhanced variants of cyan fluorescent protein (eCFP; BD Bioscience Clontech, TaKaRa Bio Europe, Saint Germain en Laye, France) by a standard PCR extension overlap technique [39]. The muscarinic receptor FRET sensors were obtained as previously reported [8,12,40]. All the resulting constructs were cloned into pcDNA3 (Invitrogen, Thermo Fisher Scientific GmbH, Dreieich, Germany) and verified by sequencing, performed by Eurofins Medigenomix GmbH.

#### 3.2.2. Stable Cell Line Generation

Cells were seeded into a culture dish with a confluency of 30% 3 h before transfecting the cells with the Effectene reagent ordered from Quiagene. Reagent concentrations and incubation times were applied in accordance with the manufacturer’s instructions. Twenty-four hours after transfecting, the normal culture medium was replaced by a culture medium supplemented with 400 μg mL^−1^ G-418. After that, the medium was refreshed every day until all the untransfected cells died. Now the cells were counted, diluted, and applied to 48-well plates, resulting in a one cell to well distribution. This homogeneous cell population was characterized by fluorescence microscopy and was investigated concerning its cDNA content.

#### 3.2.3. Cell Culture

HEK293 cells stably expressing the muscarinic receptor FRET sensors were maintained in DMEM with 4.5 g L^−1^ glucose, 10% (*v*/*v*) FCS, 100 U mL^−1^ penicillin, 100 μg mL^−1^ streptomycin sulfate and 2 mM L-glutamine, and 200 μg mL^−1^ G-418. The cells were kept at 37 °C in a humidified 7% CO_2_ atmosphere and were routinely passaged every 2 to 3 days. Untransfected HEK cells were maintained in cell culture medium without G-418.

#### 3.2.4. FlAsH Labeling

A labeling protocol was applied as described previously [2,41,42]. In brief, cells were grown to near confluency on poly-D-lysine-coated glass coverslips. Initially, cells were washed with labeling buffer (150 mM NaCl, 10 mM HEPES, 2.5 mM KCl, 4 mM CaCl_2_, 2 mM MgCl_2_, supplemented with 10 mM glucose (pH 7.3)). After that, cells were incubated with labeling buffer containing 500 nM FlAsH and 12.5 μM 1,2-ethanedithiol (EDT) for 1 h at 37 °C, followed by flushing with labeling buffer. To reduce nonspecific FlAsH binding, the cells were incubated for 10 min with labeling buffer containing 250 μM EDT. After flushing with labeling buffer, the cells were held in cell culture medium.

#### 3.2.5. Ligand Application

The reference ligands were prepared from 1 mM stock solutions that were stored at −20 °C, taking into consideration that at least acetylcholine remains unstable in solution [43]. Used stock solutions have not been older than a couple of weeks. Bitopic ligands or analogs were stored at 4 °C and were weighed out directly before the experiment. Then, the ligands were solubilized in a measuring buffer (140 mM NaCl, 10 mM HEPES, 5.4 mM KCl, 2 mM CaCl_2_, 1 mM MgCl_2_ (pH 7.3)) to a final concentration of 100 μM.

#### 3.2.6. Single Cell FRET Experiments

FRET measurements were performed using a Zeiss Axiovert 200 inverted microscope endowed with a PLAN-Neofluar oil immersion 100 objective, a dual emission photometric system, and a Polychrome IV light source (Till Photonics, Gräfelfing, Bavaria, Germany) as described previously [1,2]. Samples were excited at 436 nm (dichroic 460 nm) with a frequency of 10 Hz. Emitted light was recorded using 535/30 nm and 480/40 nm emission filters and a DCLP 505 nm beam splitter for FlAsH and CFP, respectively. FRET was observed as the ratio of FlAsH/CFP, which was corrected offline for bleed-through, direct FlAsH excitation, and photobleaching. To investigate changes in FRET on ligand addition, cells were continuously superfused with FRET buffer complemented with various ligands at saturating concentrations, as indicated. Superfusion was performed using the ALA-VM8 (ALA Scientific Instruments).

#### 3.2.7. Data Processing

Fluorescence intensities were acquired using Clampex (Axon Instruments, Molecular Devices, San Jose, CA, USA). Data are shown as means ± SD for n independent experiments. Statistical analysis and curve fitting were performed using Origin (OriginLab Corporation, Northampton, MA, USA), or Clampfit (Axon Instruments, Molecular Devices, San Jose, CA, USA).

## 4. Conclusions

In this study, two series of Xanomeline/77-LH-28-1 hybrid compounds, **12-Cn** and **13-Cn**, were designed, synthesized, and evaluated by FRET for their interaction with the four muscarinic M_1_, M_2_, M_4_, and M_5_ mAChR subtypes. This investigation provided additional information on ligand-receptor interactions between mAChRs and bitopic ligands at the molecular level. Hybrid compounds **12-C5**, **12-C7**, and **12-C9** evidenced a selective activation of the M_1_ mAChR, while hybrids **13-C5**, **13-C7**, and **13-C9** displayed a degree of selectivity for the M_1_ as well as M_4_ muscarinic receptors, thus showing a subtype activation pattern comparable to that described for Xanomeline. The optimal linker length of seven methylene groups detected for these pyridinium salts reflects a defined distance between the allosteric and the orthosteric binding regions at the M_1_ mAChR. The presence of a fixed positive charge on the orthosteric moiety of the hybrid limits the ligand conformational mobility and modulates the degree of receptor activation as a function of the spacer length. This partial agonist profile qualitatively matches those previously assessed in both M_1_ and M_2_ subtypes for various bitopic hybrid derivatives containing the super agonist Iperoxo as the preferential orthosteric moiety. In the case of dual-steric compounds, their partial agonism has been rationalized by the existence of a dynamic equilibrium between the fraction of purely allosteric (inactive) versus dual-steric (active) binding modes.

On the other hand, owing to their weaker interaction at the orthosteric binding site, the tertiary amine hybrids **12-Cn** adopted a less defined conformation within the M_1_ receptor protein. As a consequence, their receptor activation pattern is almost independent of the spacer length. Notably, the tertiary amine hybrid **12-C3**, which contains the shortest linker analyzed in this study, induced a detectable conformational change only at the G_i_-coupled M_2_ and M_4_ mAChRs, whereas the permanently charged analog **13-C3** did not show any receptor activation at all the subtypes.

In conclusion, our findings further account for the utility of bitopic molecular probes for an in-depth exploration of dynamic ligand-receptor interactions of mAChR subtypes. More generally, the structural features of rationally designed hybrid ligands, in particular the nature and length of the spacer moiety, may allow for the tuning of a GPCR activation profile, with a chance to accomplish functionally selective agonist/partial agonists of putative therapeutic value.

## Data Availability

Not applicable.

## Supplementary Materials

The following supporting information can be downloaded at: https://www.mdpi.com/article/10.3390/molecules28052407/s1, Characterization data of compounds **15**–**19**, **10**, **22-Cn**, **23-Cn**, **12-Cn** and **13-Cn** including ^1^H NMR, ^13^C NMR spectra, and HRMS spectra.

## Abbreviations

ACh: acetylcholine; BQCA, benzyl quinolone carboxylic acid; CFP, cyan fluorescent protein; FlAsH, fluoresceine arsenical hairpin; FRET, fluorescence resonance energy transfer; GPCRs, G protein-coupled receptors; IL, intracellular loop; mAChRs, muscarinic acetylcholine receptors; TM, transmembrane region.
