# Supplementary material for: Novel Xanomeline-Containing Bitopic Ligands of Muscarinic Acetylcholine Receptors: Design, Synthesis and FRET Investigation
Source: Molecules. 2023 Mar 6;28(5):2407. doi: 10.3390/molecules28052407 (PMC10005175; doi:10.3390/molecules28052407)
Supplement: Supplementary file 1 [file molecules-28-02407-s001.zip › molecules-2219178-supplementary.pdf]

Supporting Information

Novel Xanomeline-Containing Bitopic Ligands of Muscarinic Acetylcholine Receptors: Design, Synthesis and FRET Investigation

Carlo Matera [1], Michael Kauk [2], Davide Cirillo [1], Marco Maspero [1], Claudio Papotto [1], Daniela Volpato [3], Ulrike Holzgrabe [3], Marco De Amici [1], Carsten Hoffmann [2] and Clelia Dallanoce [1,*]

1 Department of Pharmaceutical Sciences, Medicinal Chemistry Section "Pietro Pratesi", University of Milan, Via L. Mangiagalli 25, 20133 Milan, Italy
2 Institute for Molecular Cell Biology, Center for Molecular Biomedicine, University Hospital Jena, Friedrich Schiller University Jena, Hans Knoell Str. 2, 07745 Jena, Germany
3 Pharmaceutical and Medicinal Chemistry, Institute of Pharmacy and Food Chemistry, University of Würzburg, Am Hubland, 97074 Würzburg, Germany
* Correspondence: clelia.dallanoce@unimi.it; Tel.: +39-02-503-19327

Table of contents

Compound 15 ^{1}H NMR, ^{13}C NMR S3

Compound 16 ^{1}H NMR, ^{13}C NMR S4

Compound 17 ^{1}H NMR, ^{13}C NMR S5

Compound 18 ^{1}H NMR, ^{13}C NMR S6

Compound 19 ^{1}H NMR, ^{13}C NMR S7

Compound 10 ^{1}H NMR, ^{13}C NMR S8

Compound 22-C3 ^{1}H NMR, ^{13}C NMR S9

Compound 22-C5 ^{1}H NMR, ^{13}C NMR S10

Compound 22-C7 ^{1}H NMR, ^{13}C NMR S11

Compound 22-C9 ^{1}H NMR, ^{13}C NMR S12

Compound 23-C3 ^{1}H NMR, ^{13}C NMR S13

Compound 23-C5 ^{1}H NMR, ^{13}C NMR S14

Compound 23-C7 ^{1}H NMR, ^{13}C NMR S15

Compound 23-C9 ^{1}H NMR, ^{13}C NMR S16

Compound 13-C3 ^{1}H NMR, ^{13}C NMR, HRMS S17-S18

Compound 13-C5 ^{1}H NMR, ^{13}C NMR, HRMS S19-S20

Compound 13-C7 ^{1}H NMR, ^{13}C NMR, HRMS S21-S22

Compound 13-C9 ^{1}H NMR, ^{13}C NMR, HRMS S23-S24

Compound 12-C3 ^{1}H NMR, ^{13}C NMR S25

Compound 12-C3 Oxalate ^{1}H NMR, ^{13}C NMR, Elemental analysis, HRMS S26-S28

Compound 12-C5 ^{1}H NMR, ^{13}C NMR, HRMS S29-S30

Compound 12-C7 ^{1}H NMR, ^{13}C NMR, HRMS S31-S32

Compound 12-C9 ^{1}H NMR, ^{13}C NMR, HRMS S33-S34

Compound 15

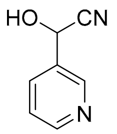

^{1}H NMR spectrum

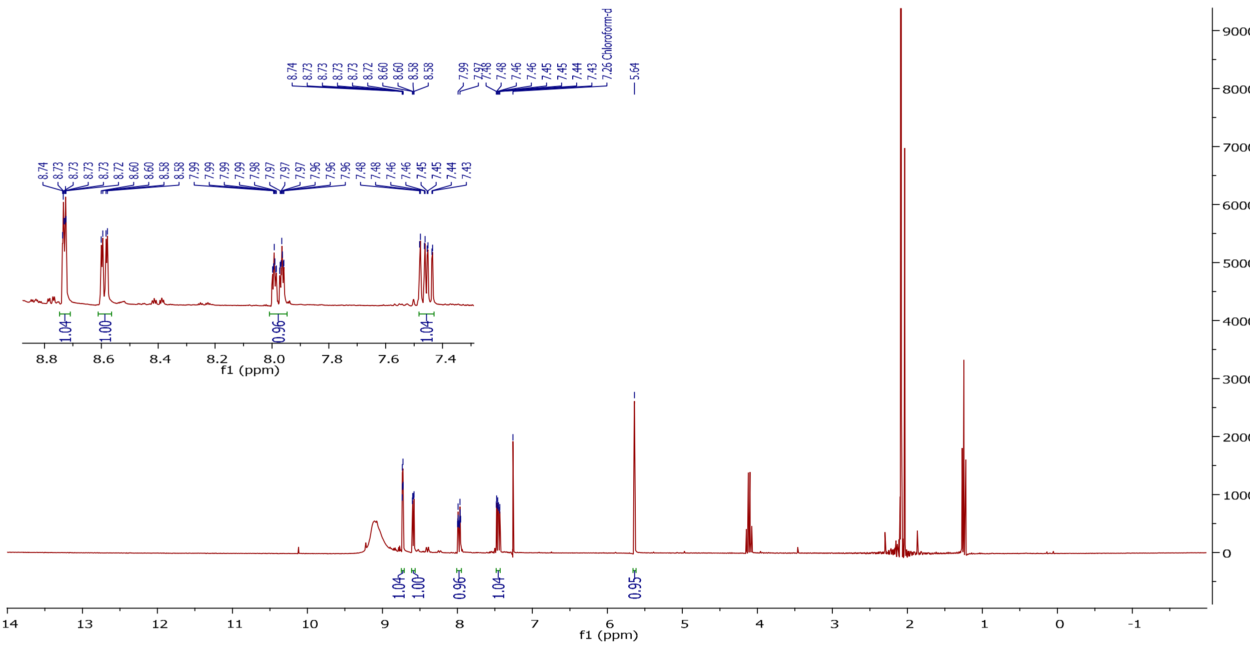

^{13}C NMR spectrum

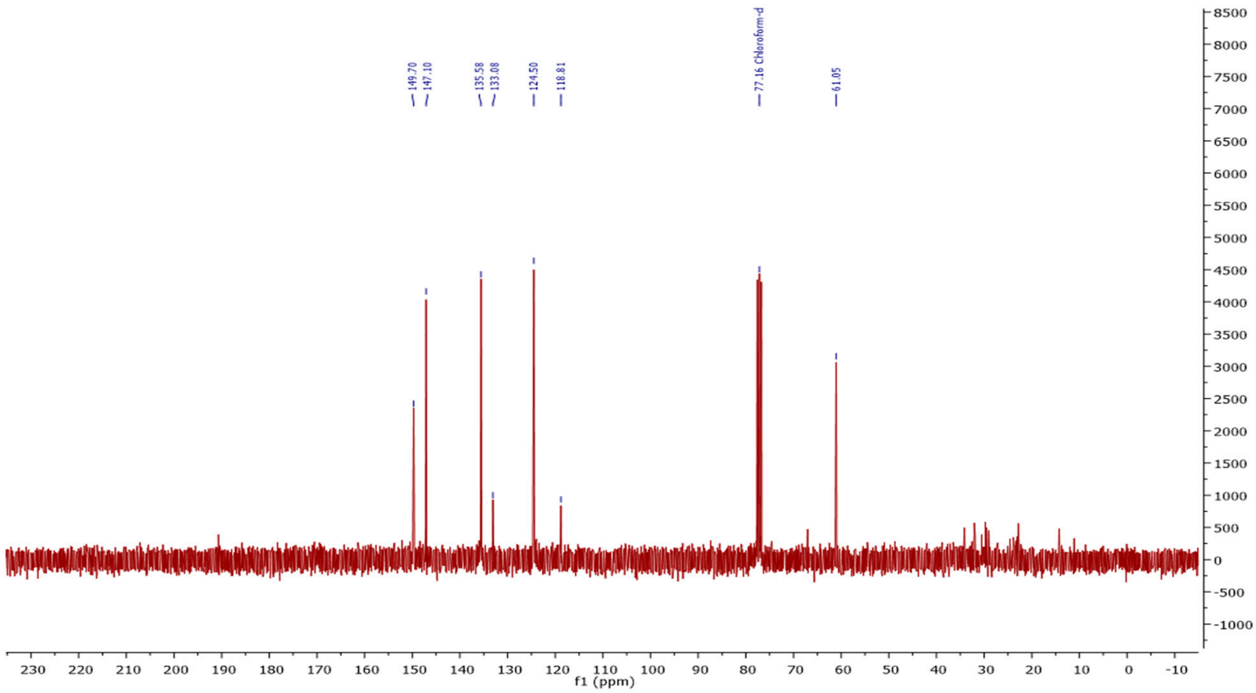

Compound 16

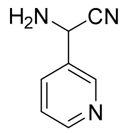

^{1}H NMR spectrum

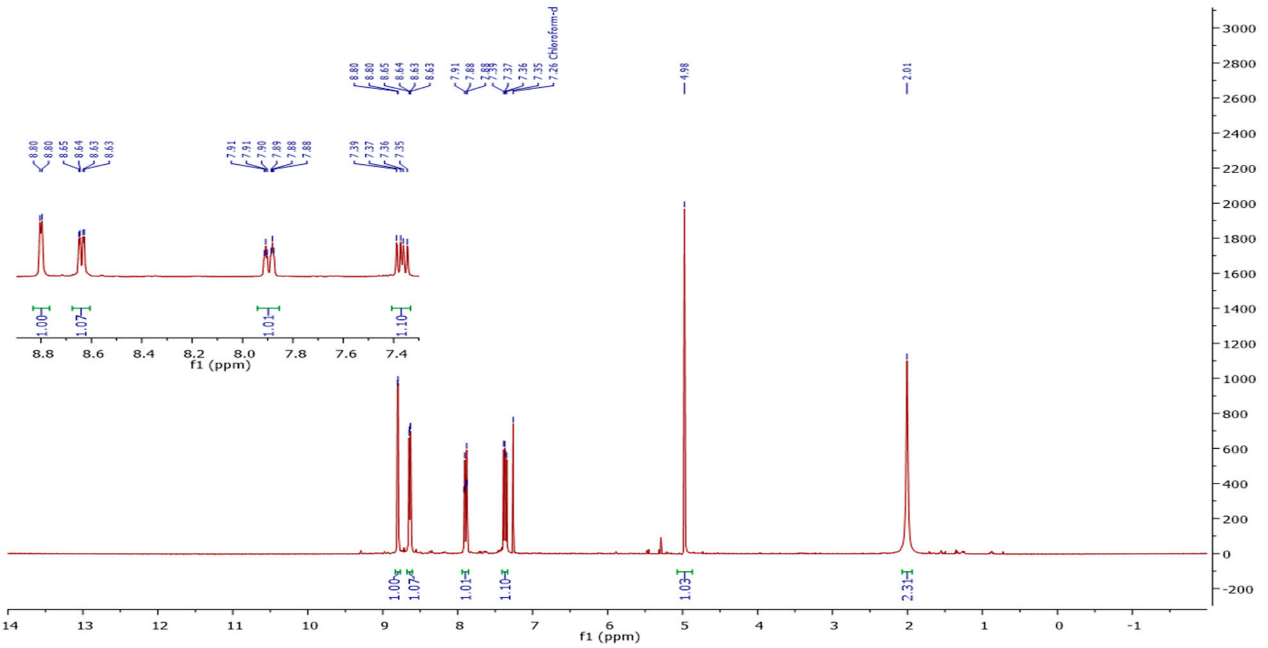

^{13}C NMR spectrum

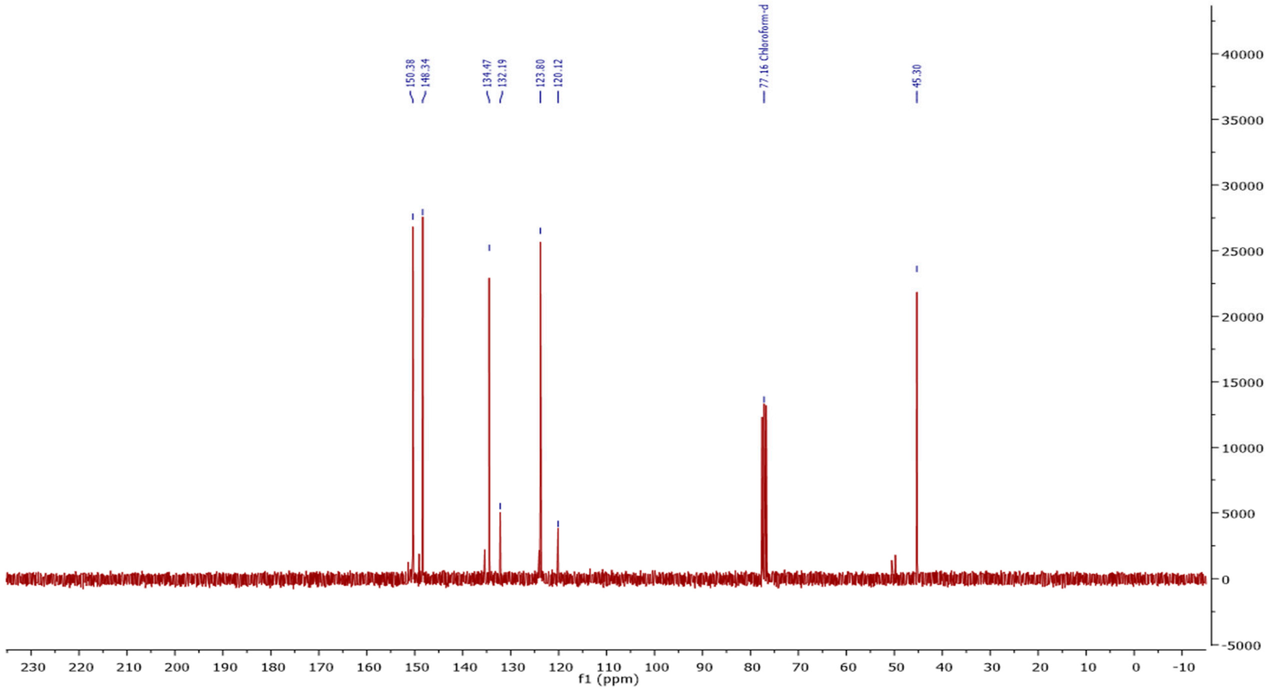

Compound 17

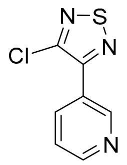

¹H NMR spectrum

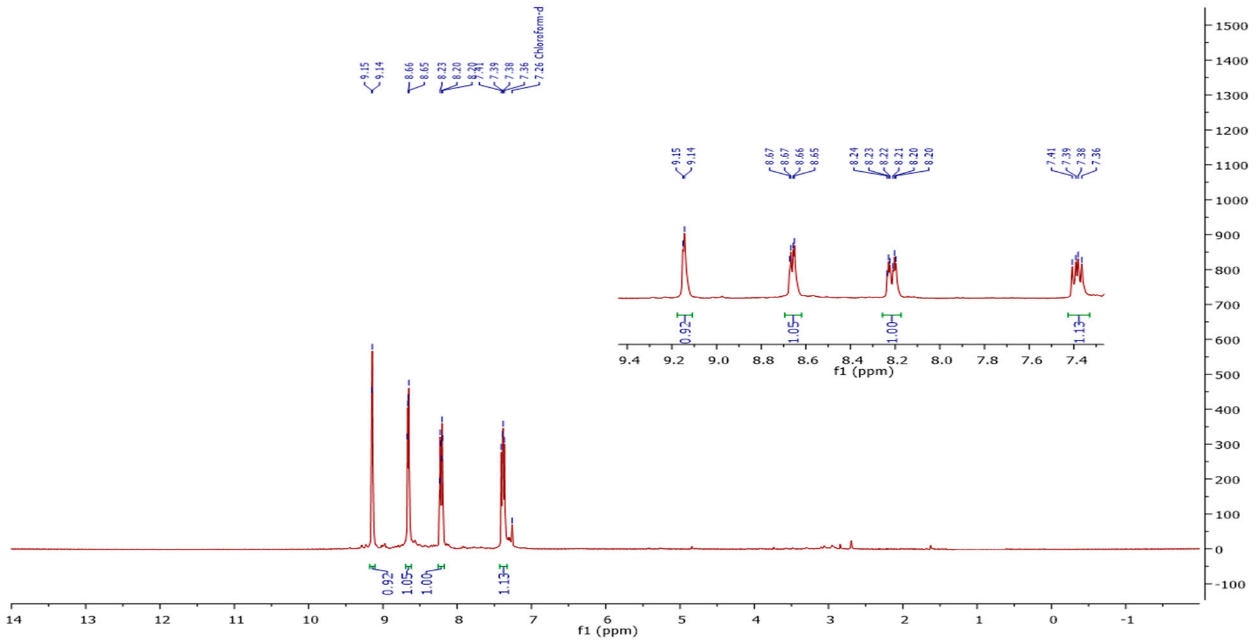

¹³C NMR spectrum

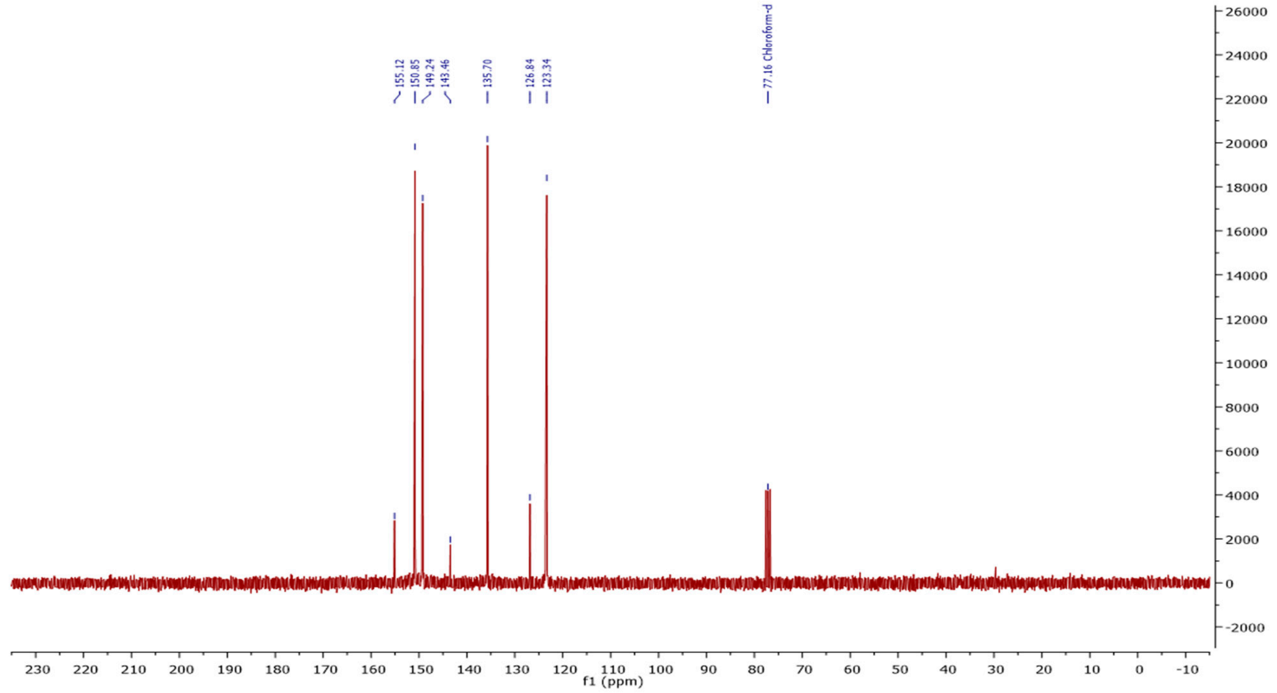

Compound 18

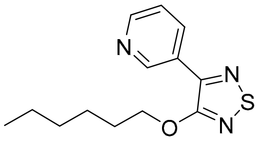

^{1}H NMR spectrum

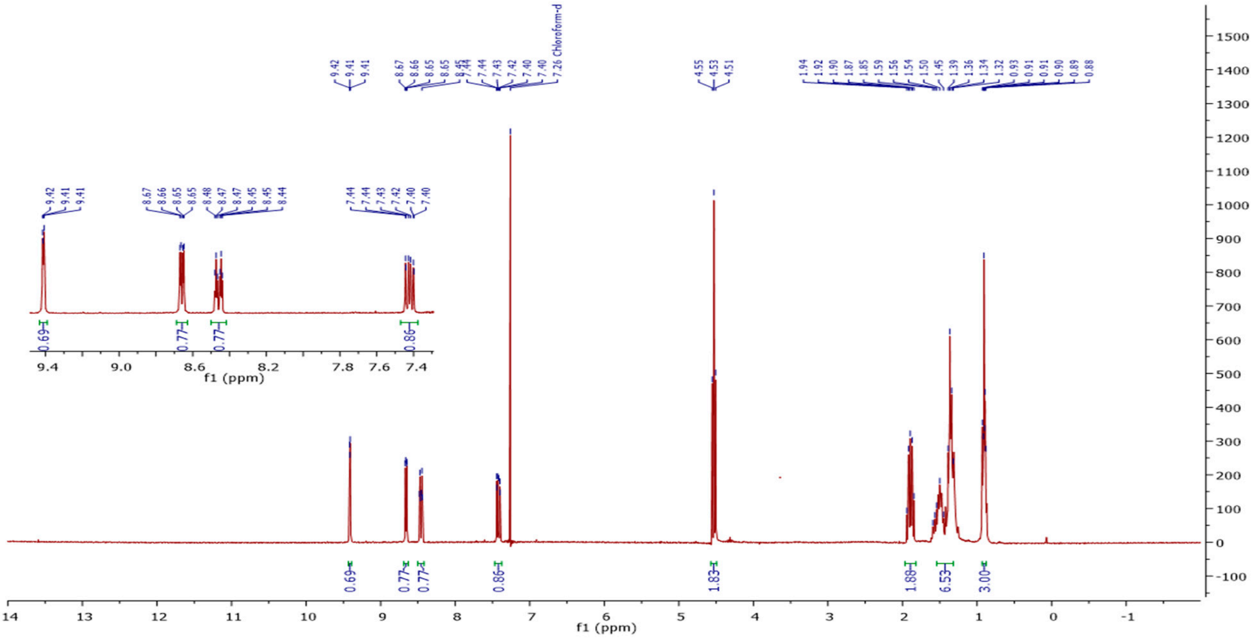

^{13}C NMR spectrum

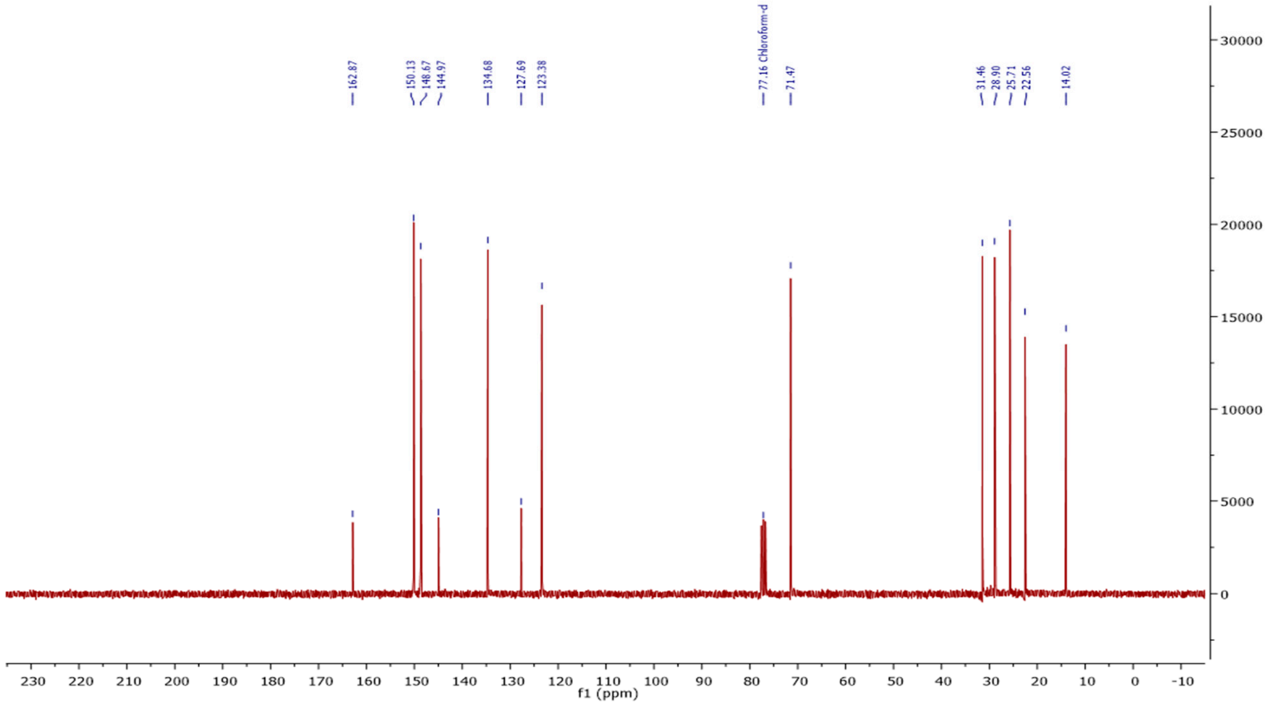

Compound 19

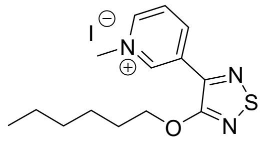

¹H NMR spectrum

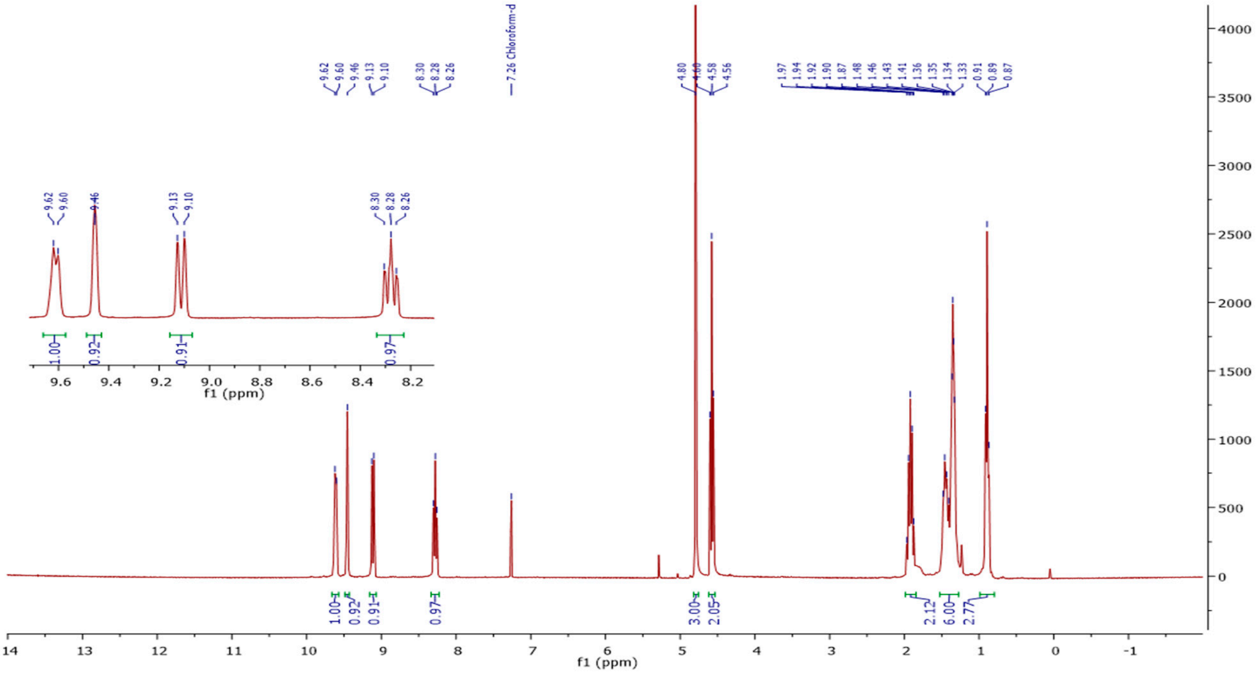

¹³C NMR spectrum

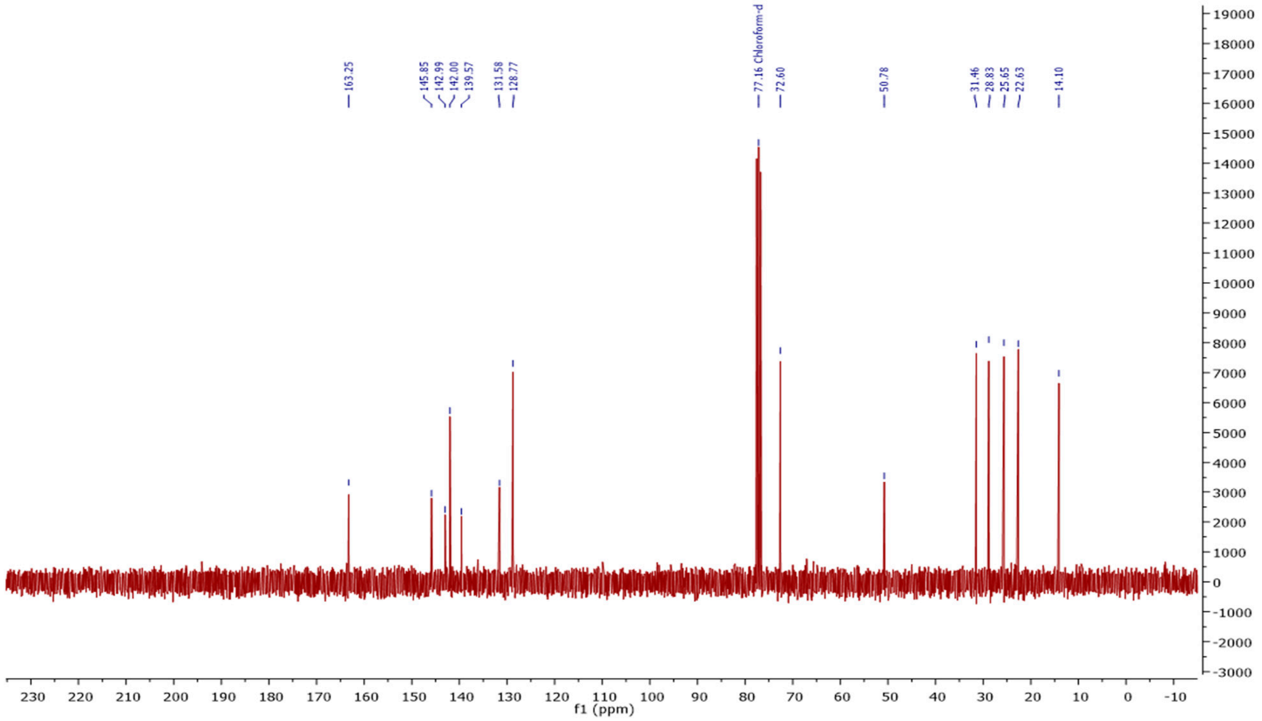

Compound 10

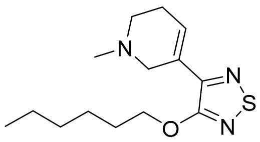

¹H NMR spectrum

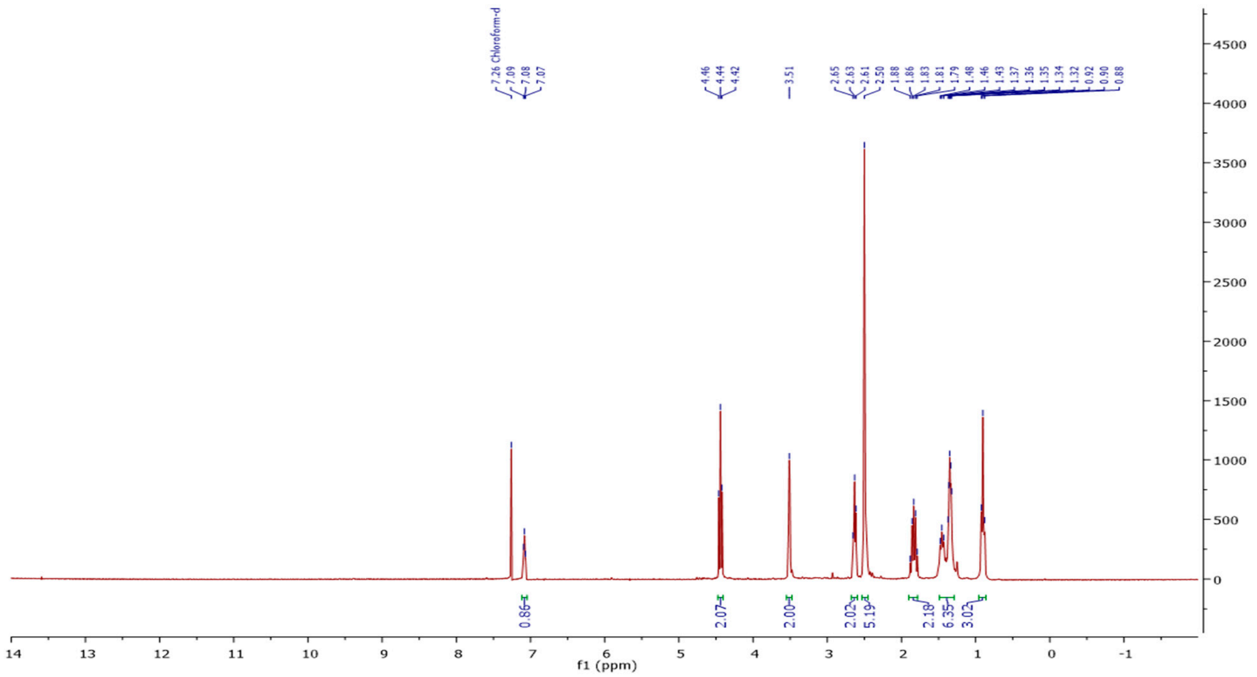

¹³C NMR spectrum

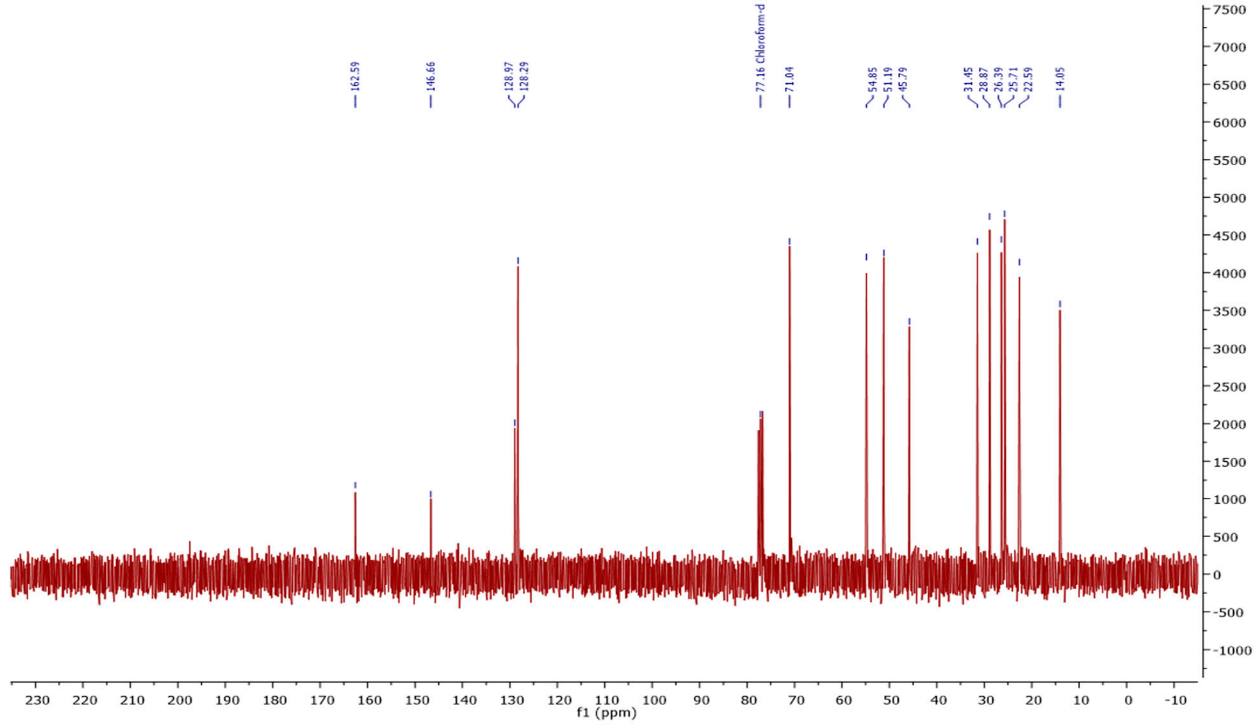

22-C3

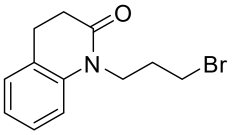

^{1}H NMR spectrum

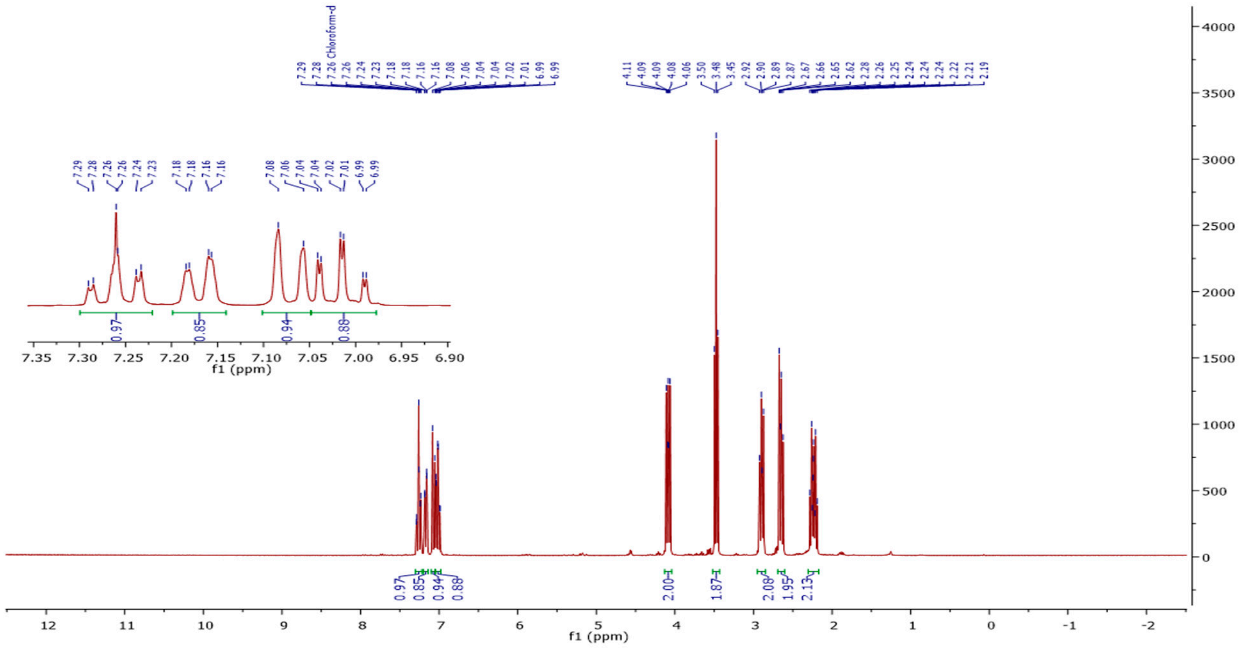

^{13}C NMR spectrum

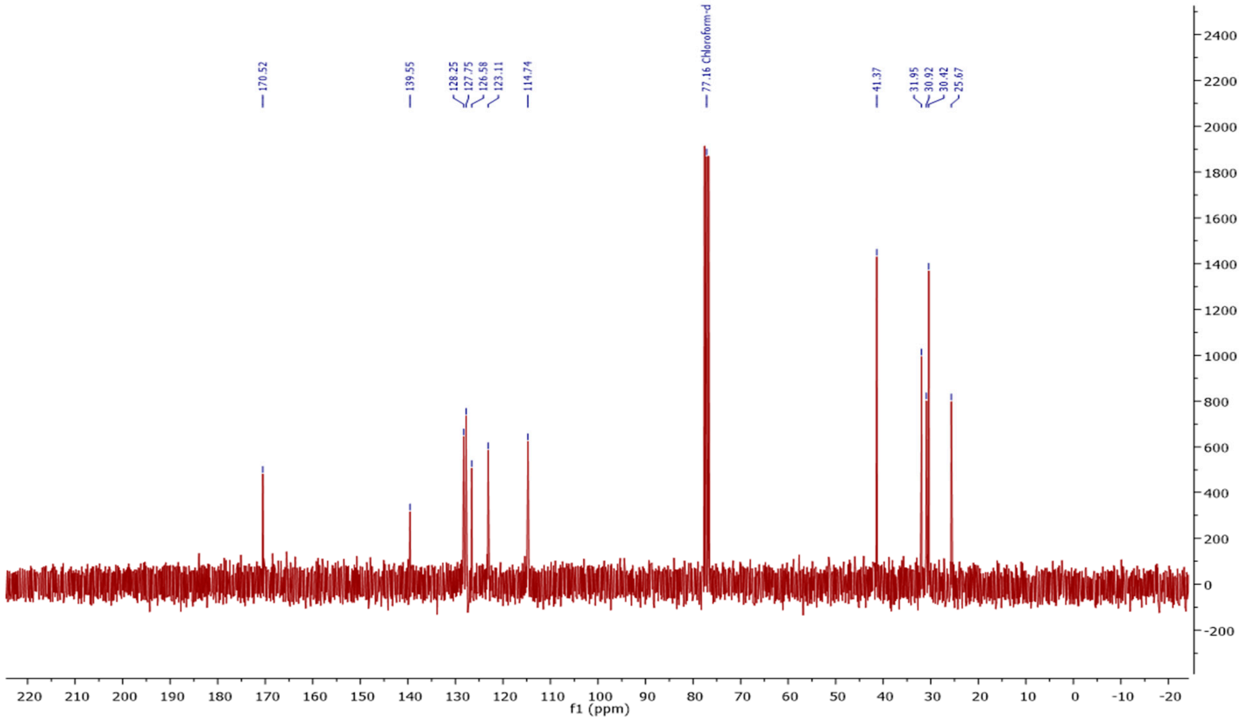

22-C5

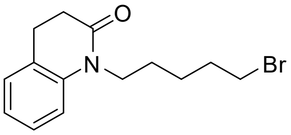

^{1}H NMR spectrum

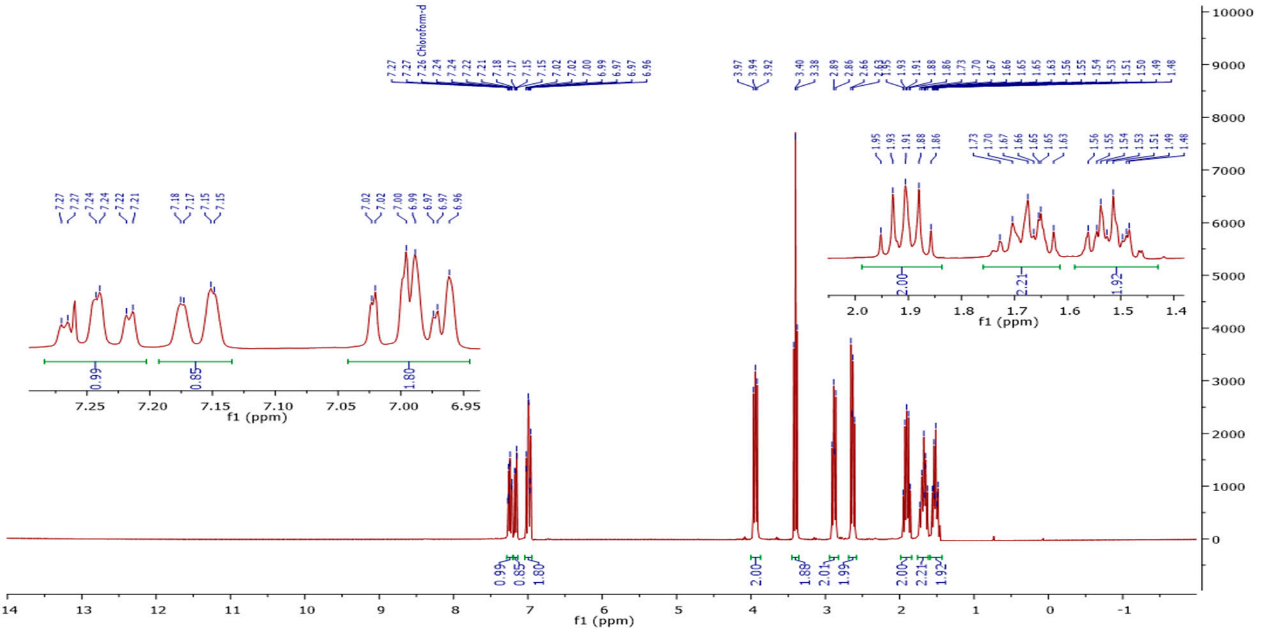

^{13}C NMR spectrum

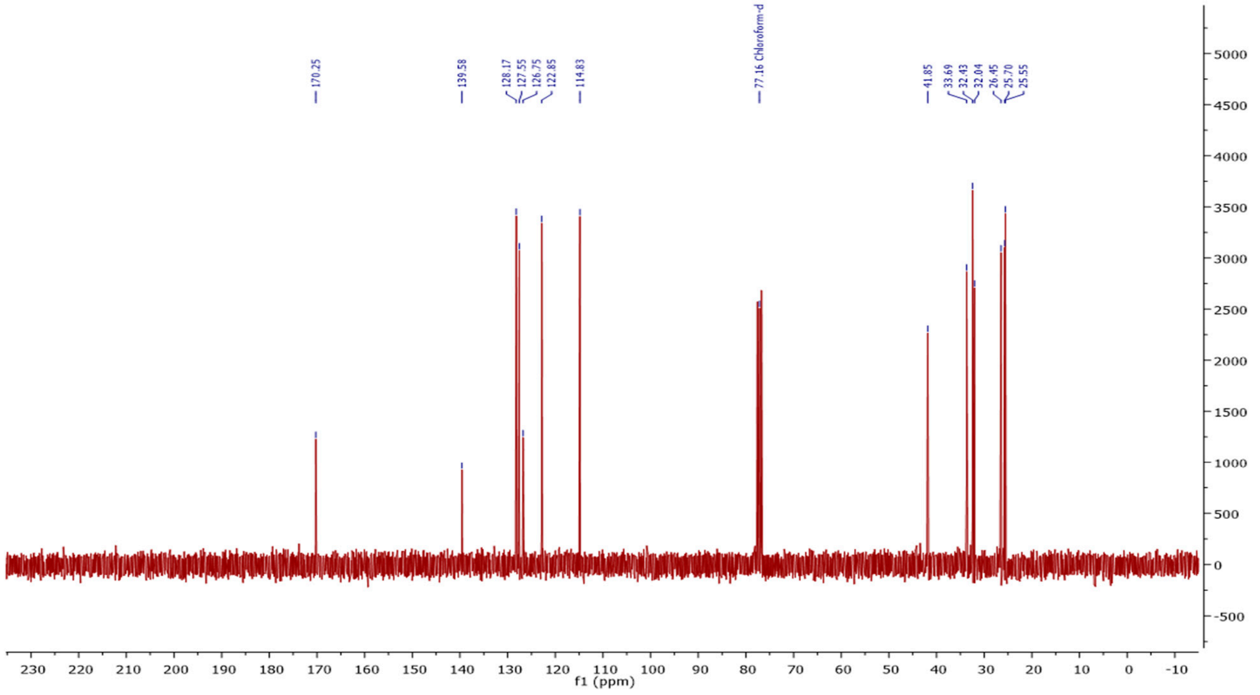

22-C7

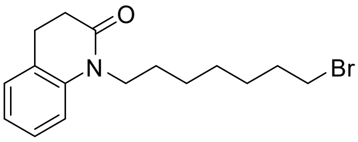

^{1}H NMR spectrum

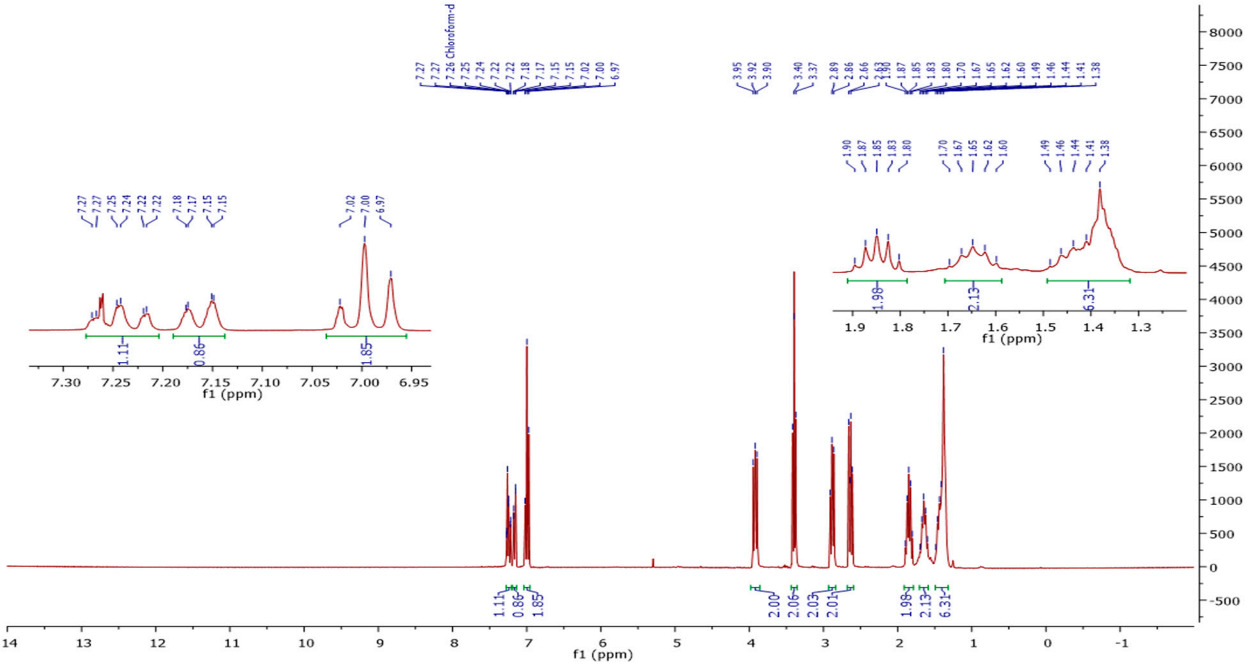

^{13}C NMR spectrum

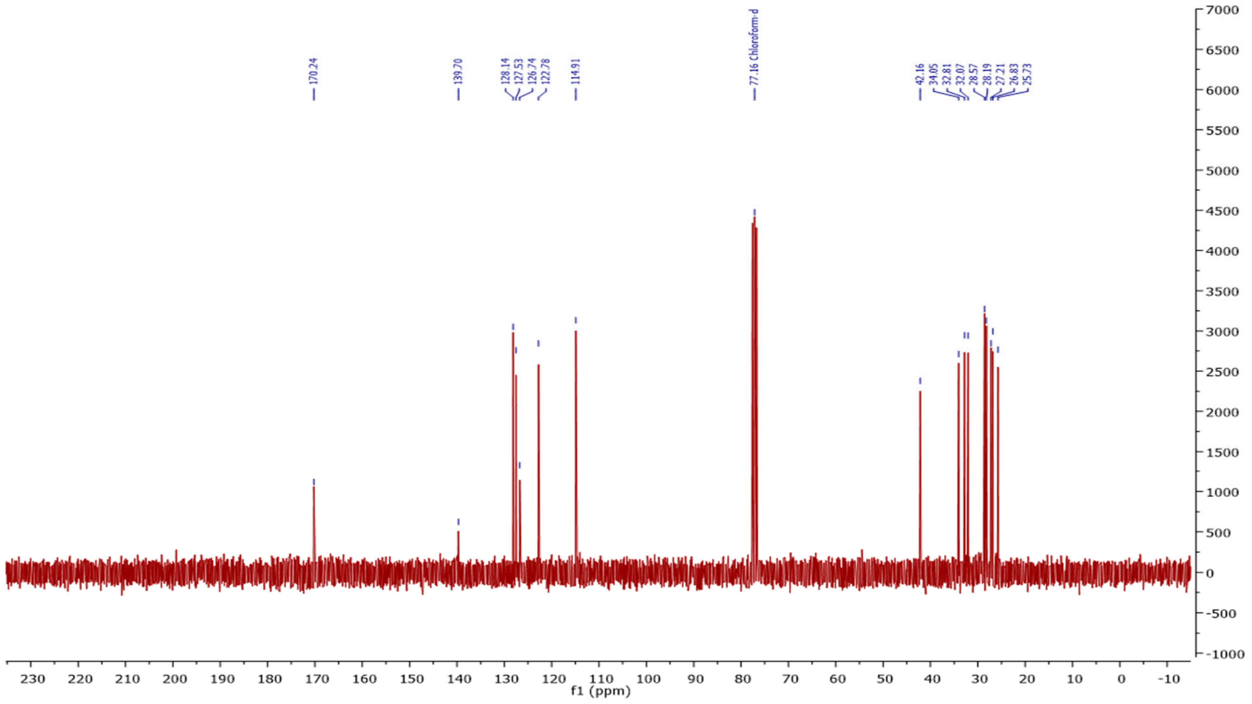

22-C9

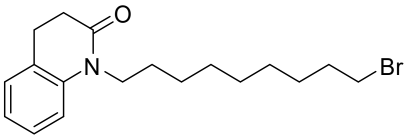

^{1}H NMR spectrum

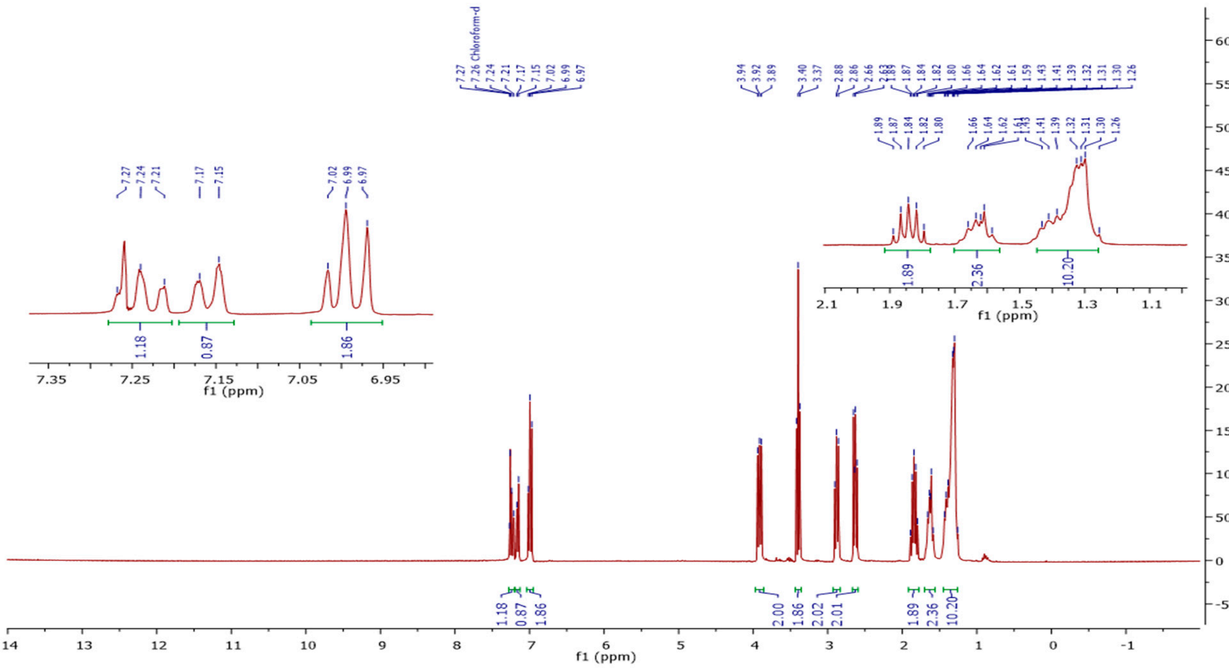

^{13}C NMR spectrum

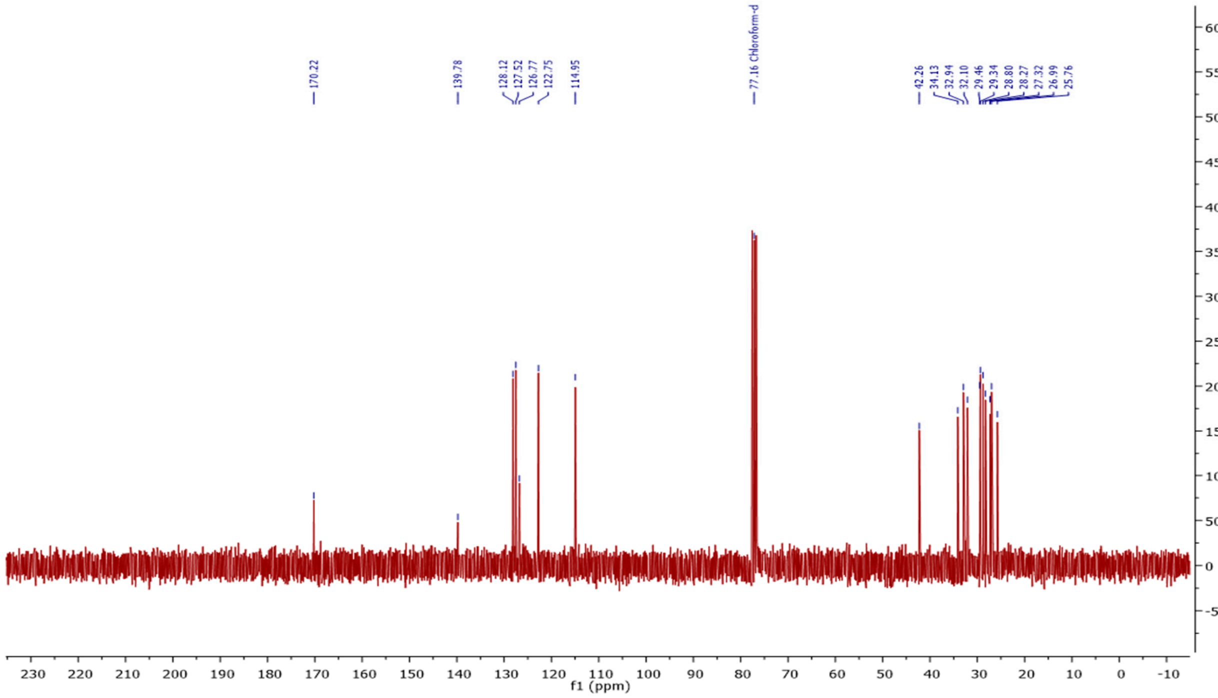

23-C3

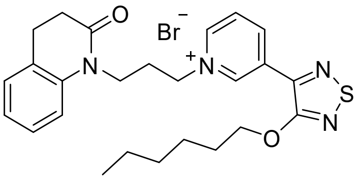

^{1}**H NMR** spectrum

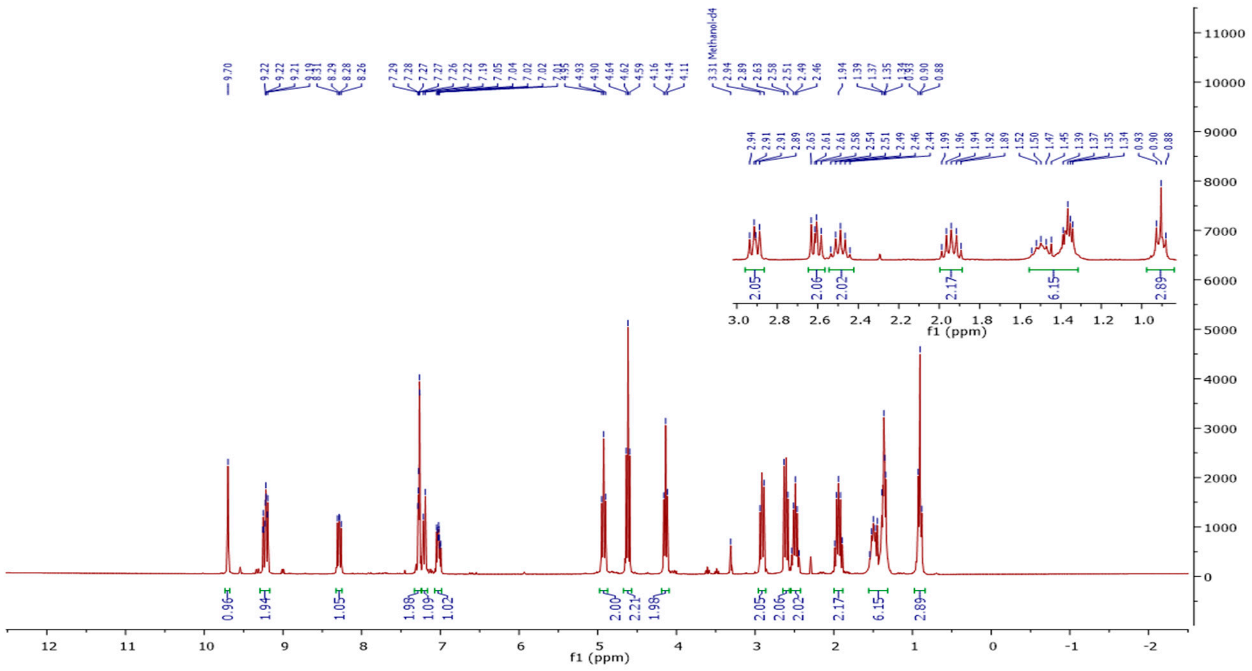

^{13}**C NMR** spectrum

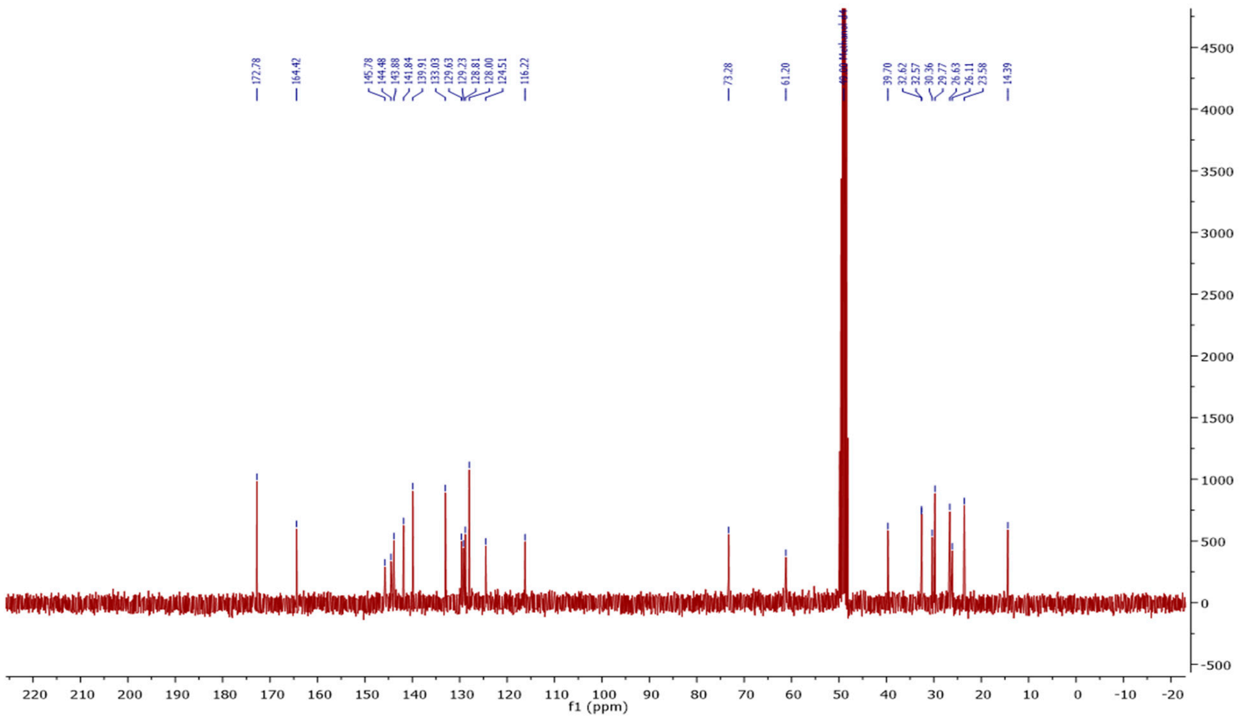

23-C5

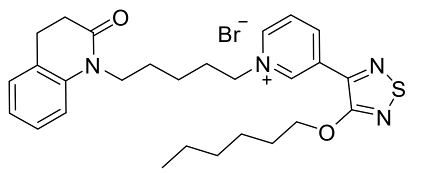

^{1}H NMR spectrum

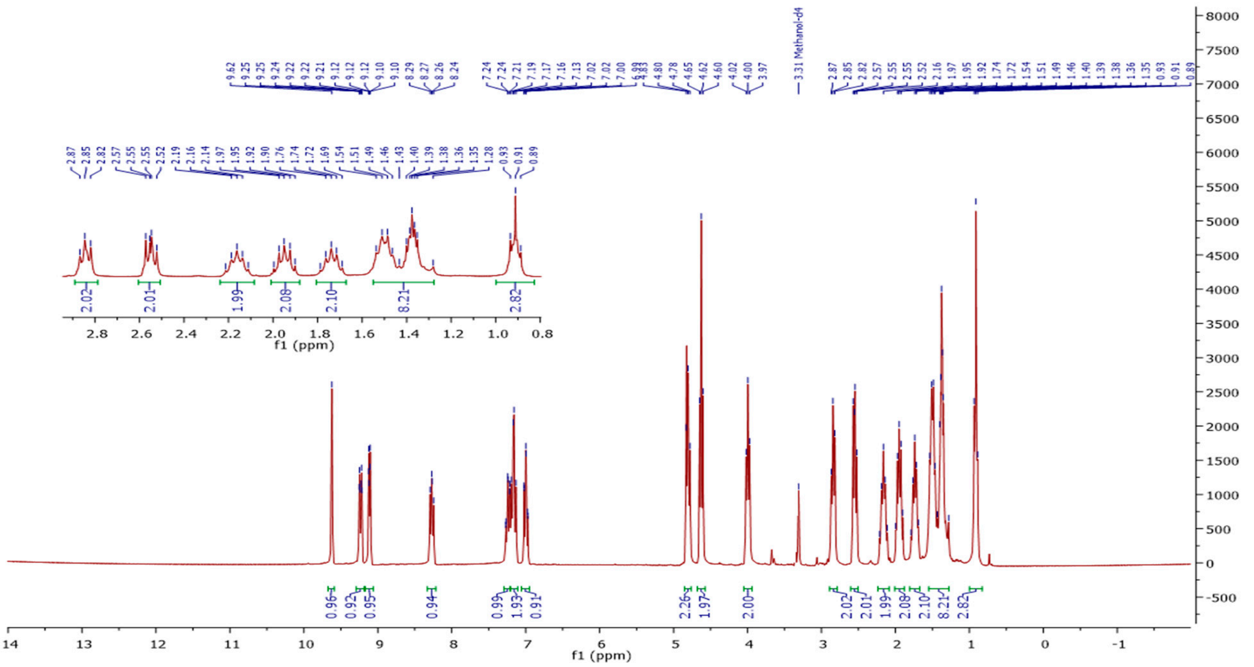

^{13}C NMR spectrum

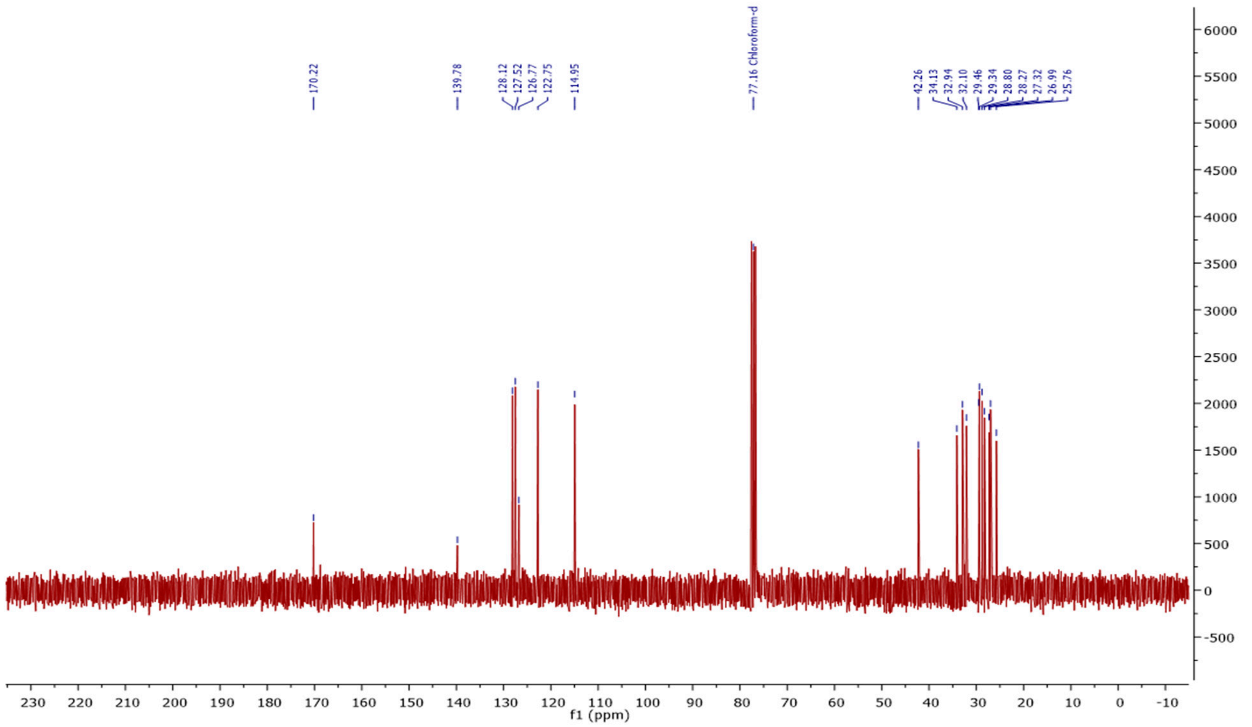

23-C7

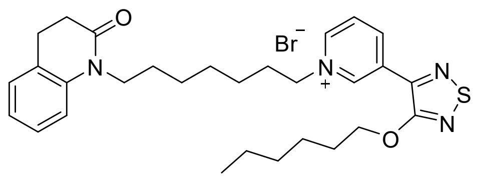

^{1}H NMR spectrum

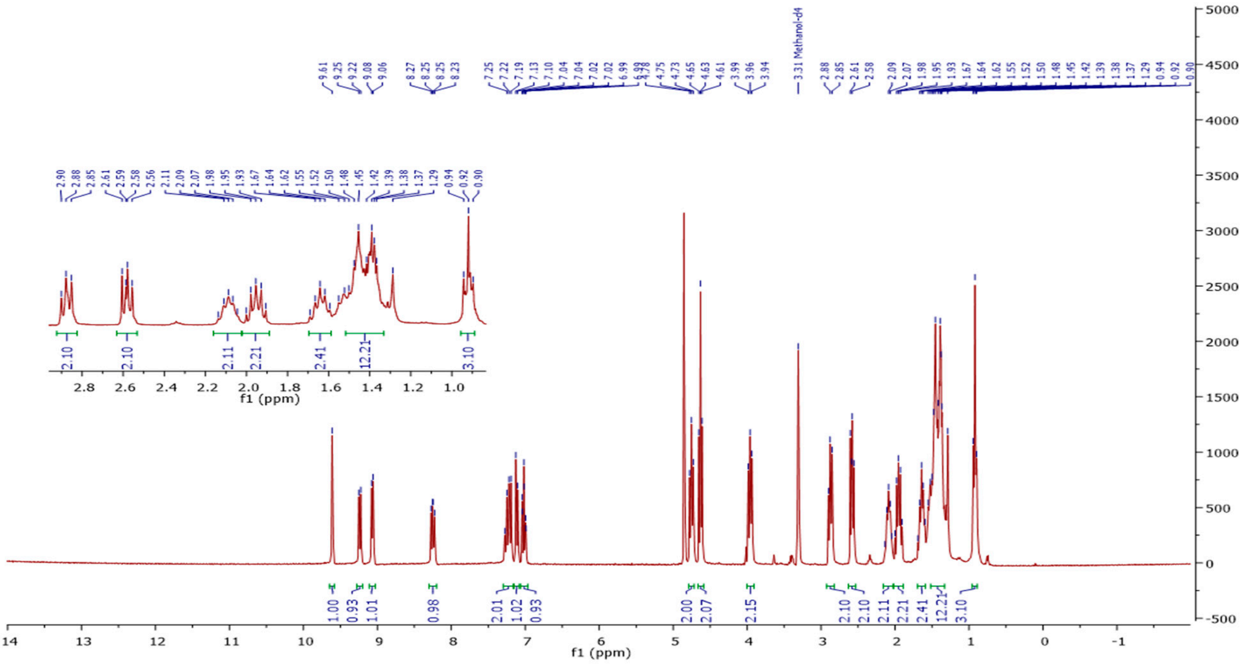

^{13}C NMR spectrum

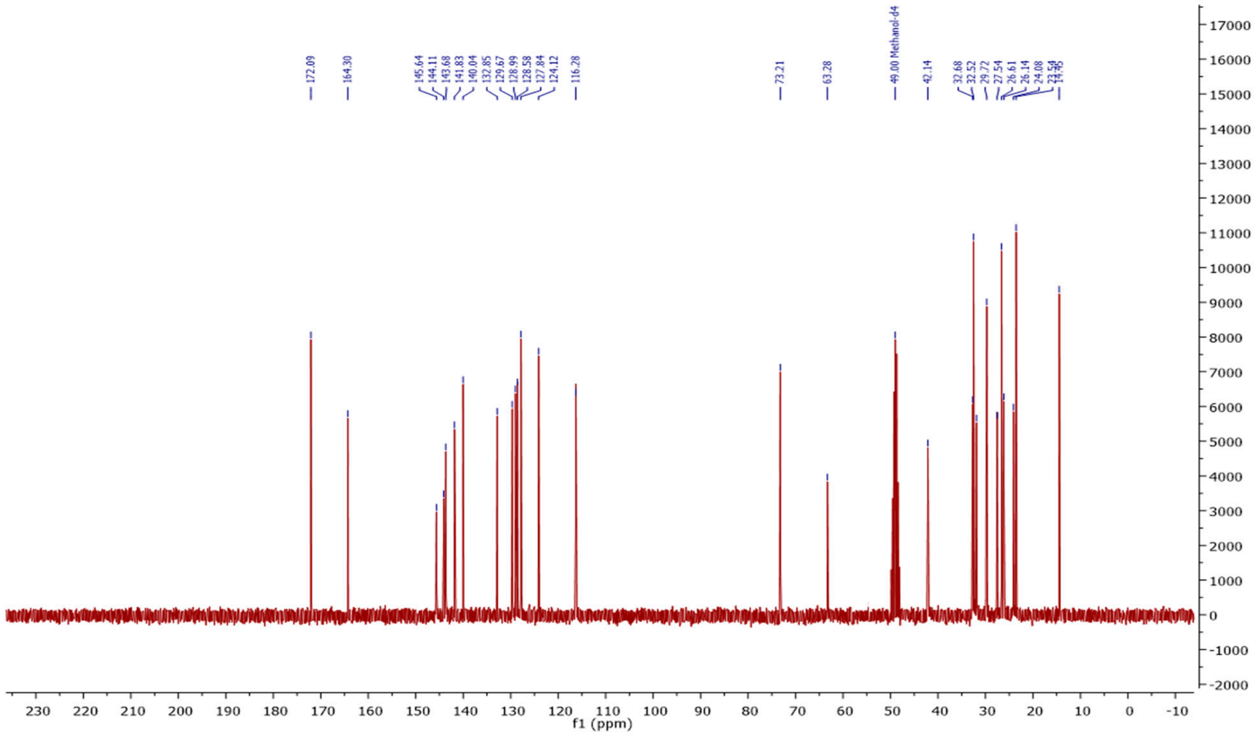

23-C9

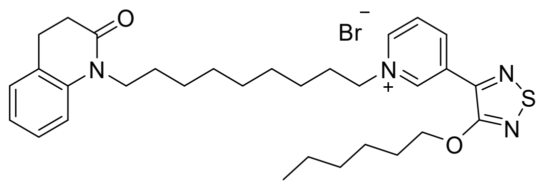

^{1}H NMR spectrum

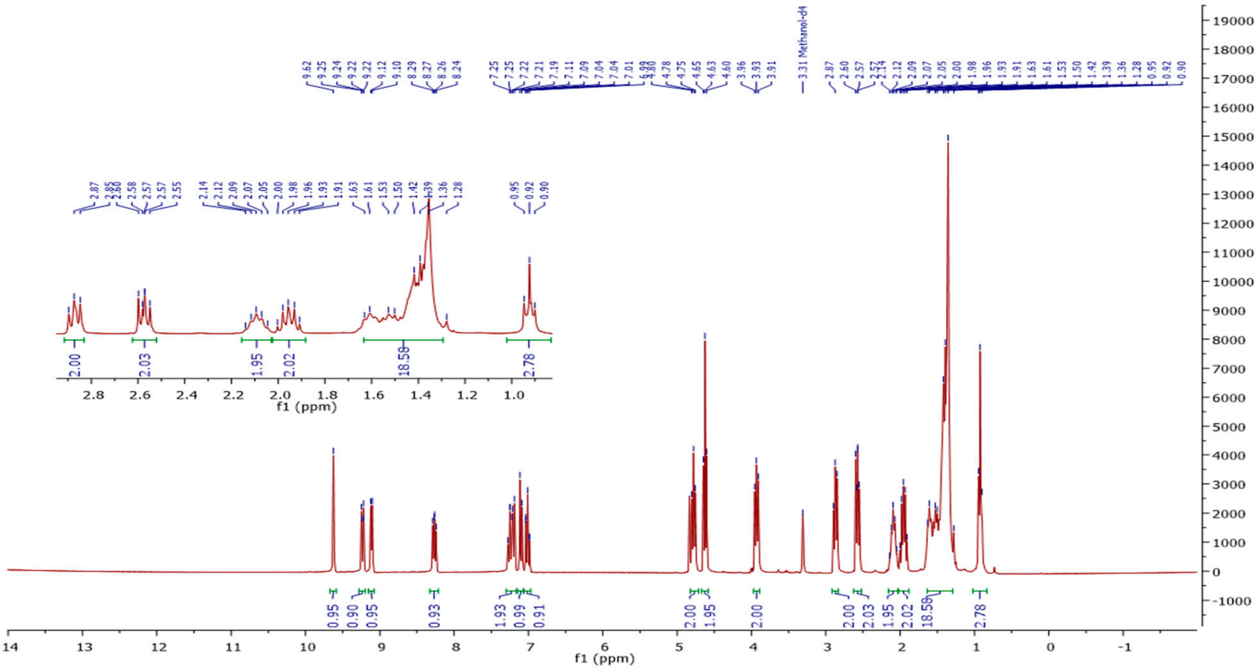

^{13}C NMR spectrum

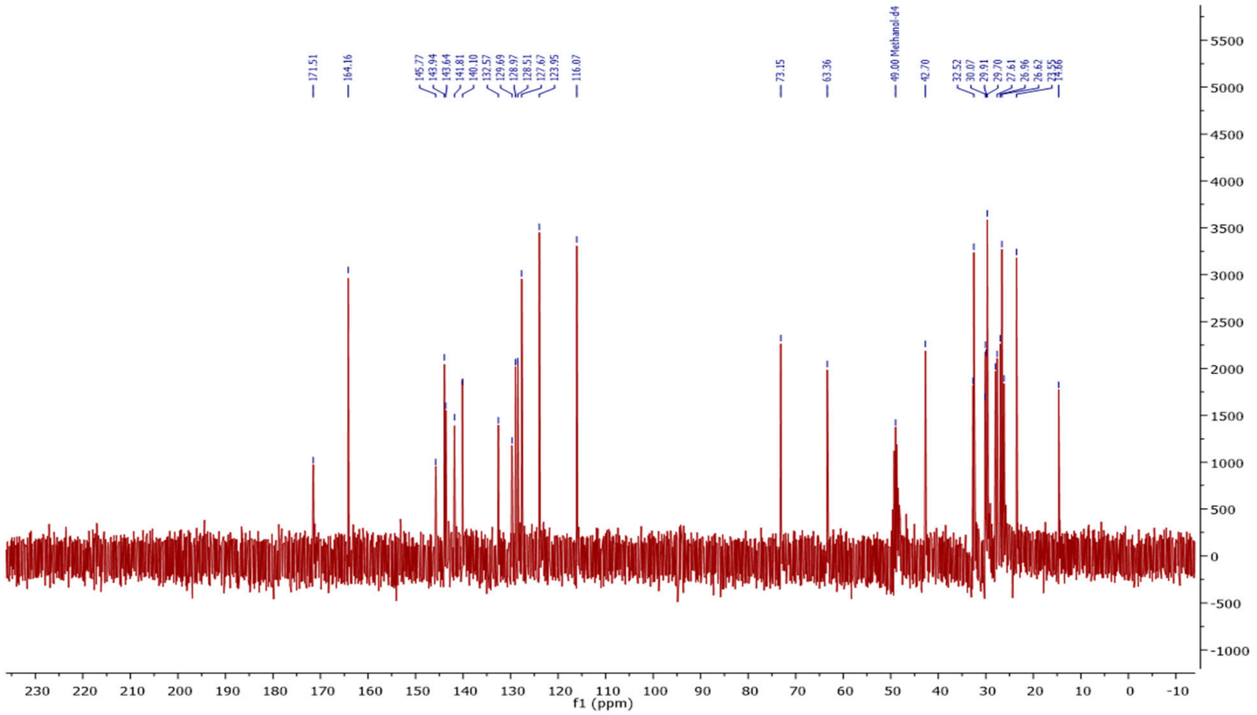

13-C3

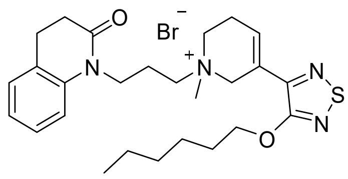

^{1}H NMR spectrum

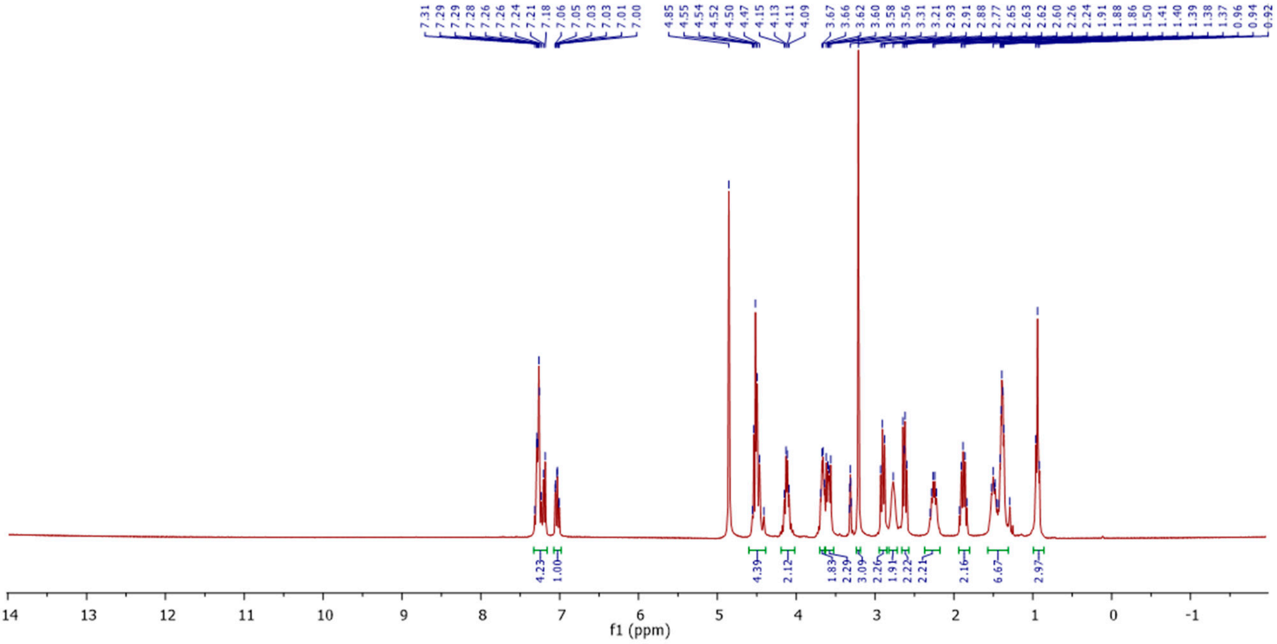

^{13}C NMR spectrum

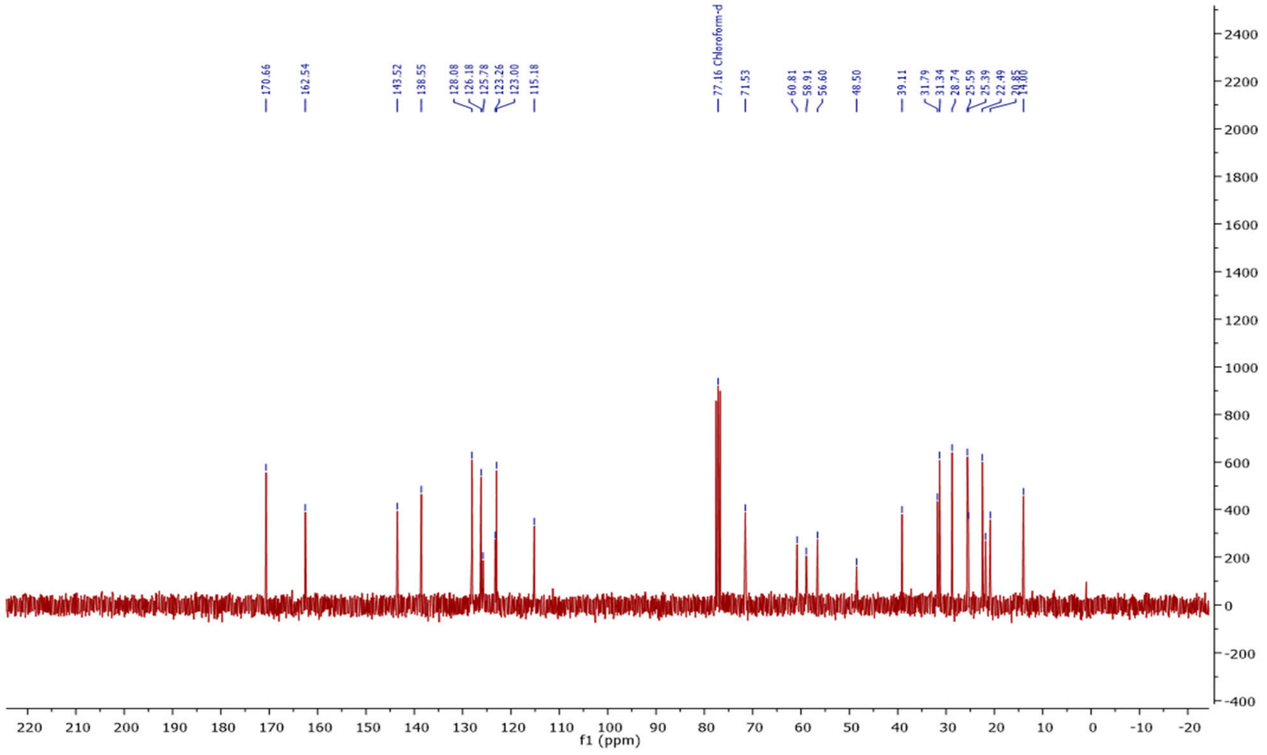

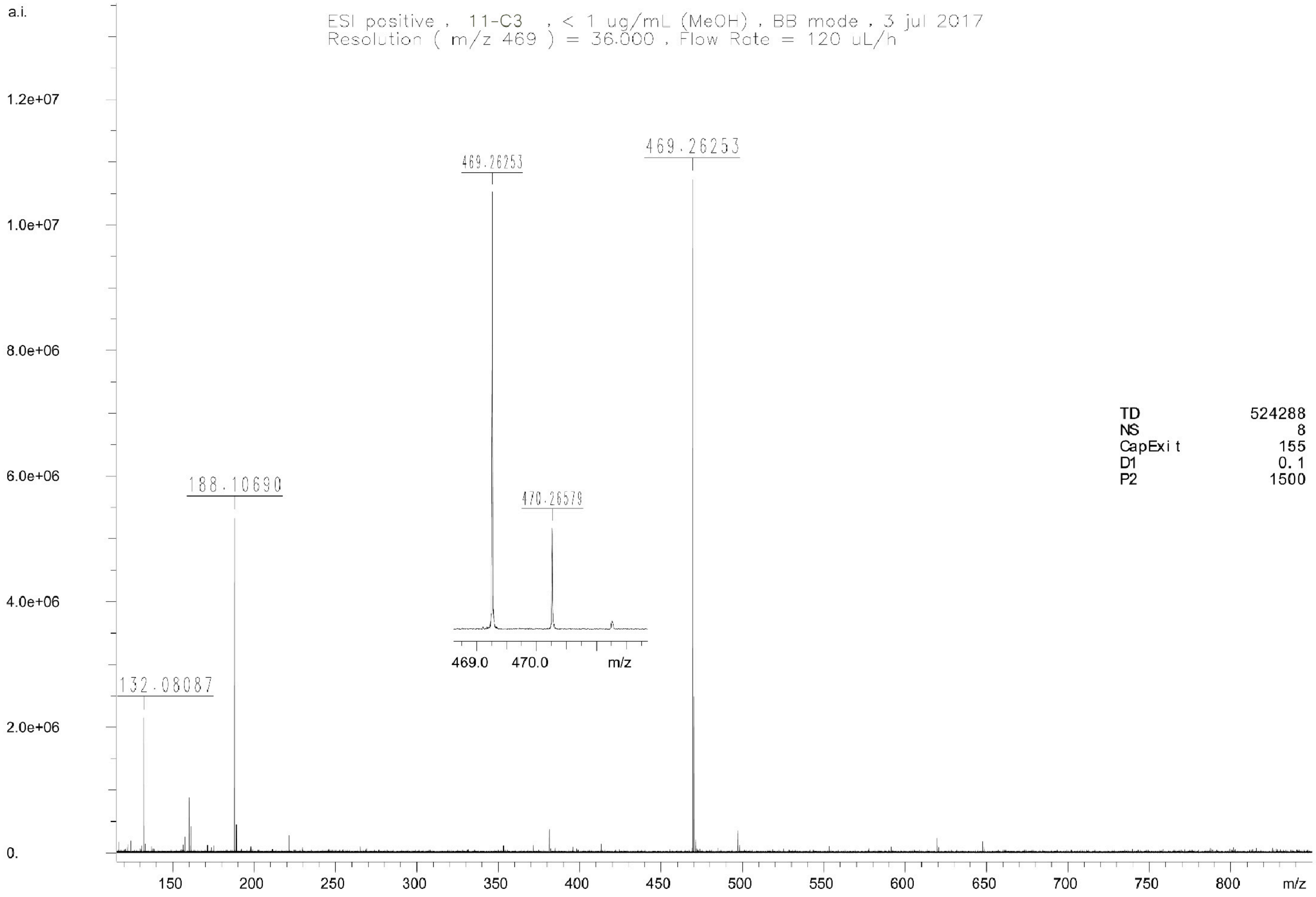

ESI positive , 11-C3 , < 1 ug/mL (MeOH) , BB mode , 3 jul 2017
Resolution (m/z 469) = 36.000 , Flow Rate = 120 uL/h
a.i.
1.2e+07
1.0e+07
8.0e+06
6.0e+06
4.0e+06
2.0e+06
0.
132.08087
188.10690
469.26253
470.26579
469.0
470.0
m/z
150
200
250
300
350
400
450
500
550
600
650
700
750
800
TD 524288
NS 8
CapExit 155
D1 0.1
P2 1500

13-C5

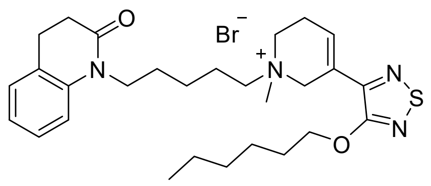

^{1}H NMR spectrum

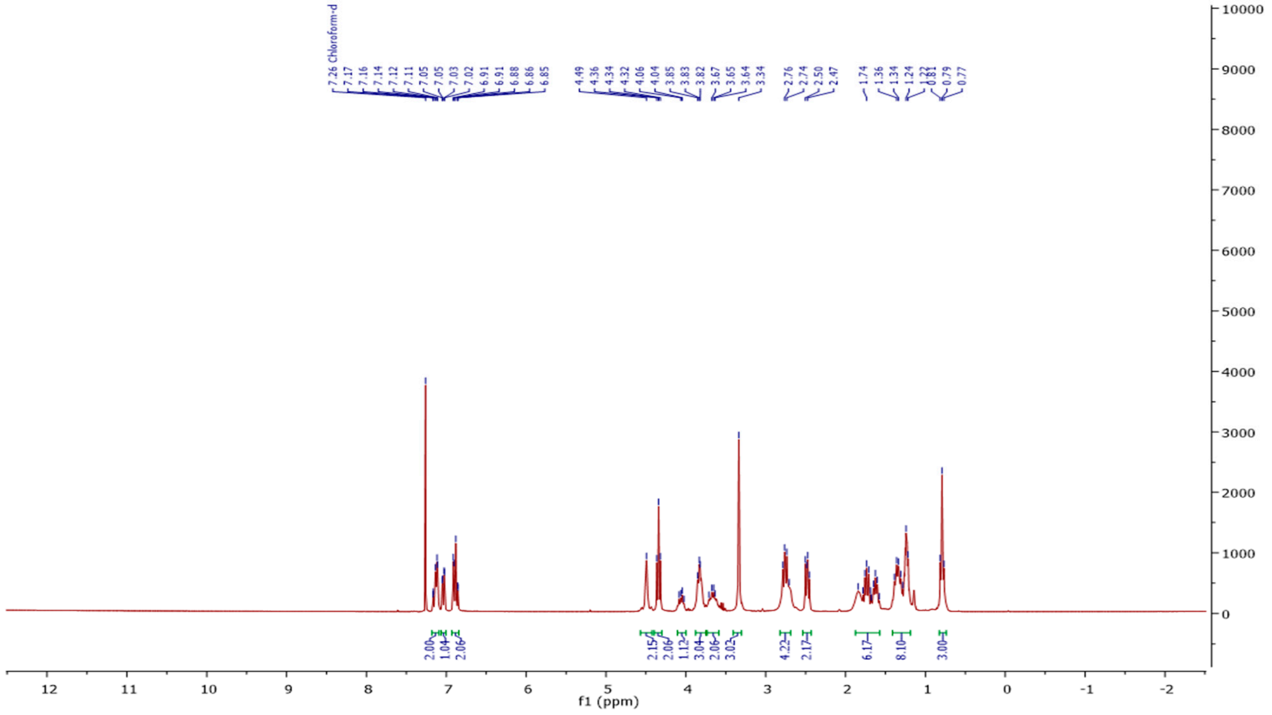

^{13}C NMR spectrum

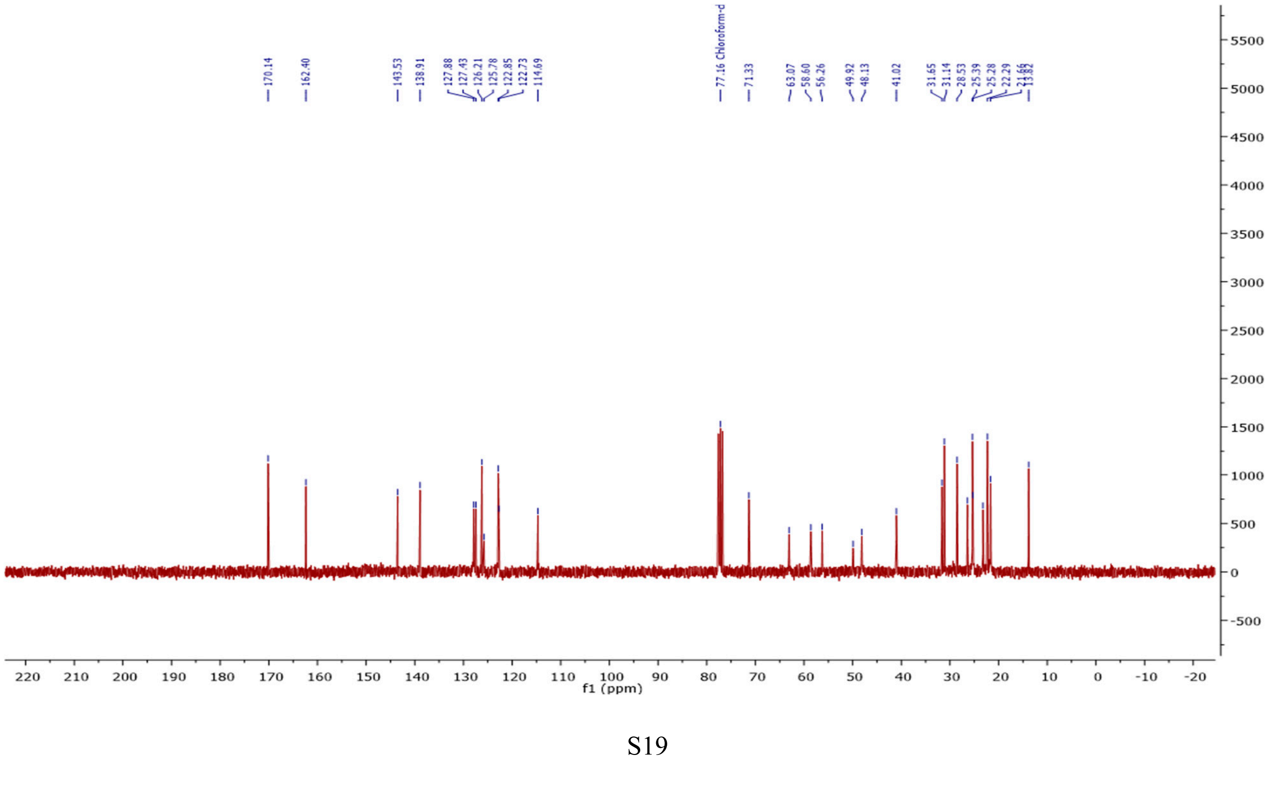

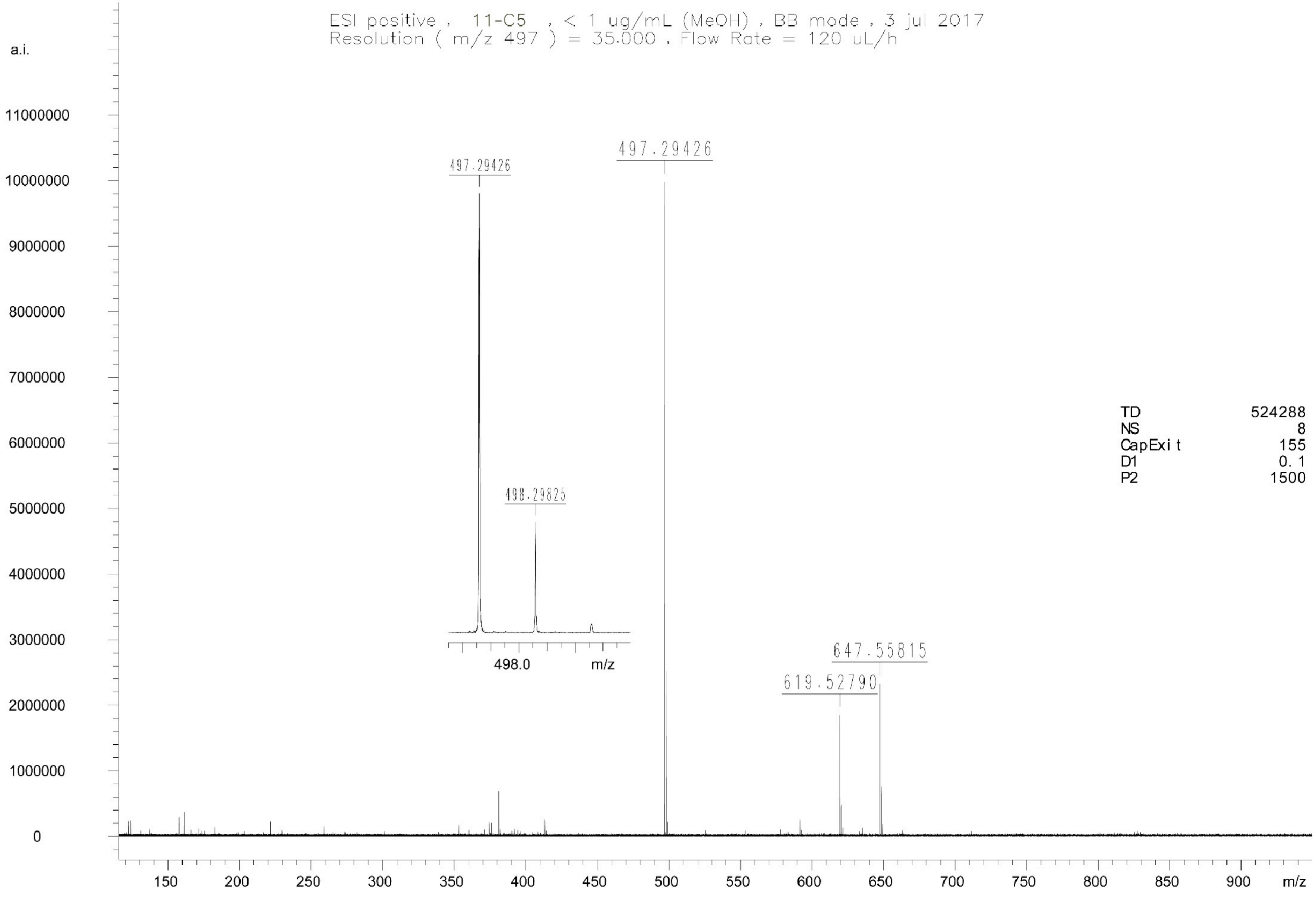
ESI positive , 11-C5 , < 1 ug/mL (MeOH) , B3 mode , 3 jul 2017
Resolution (m/z 497) = 35.000 , Flow Rate = 120 uL/h
a.i.
497.29426
498.29825
498.0
m/z
619.52790
647.55815
TD 524288
NS 8
CapExit 155
D1 0.1
P2 1500

13-C7

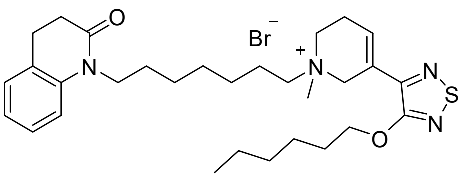

^{1}H NMR spectrum

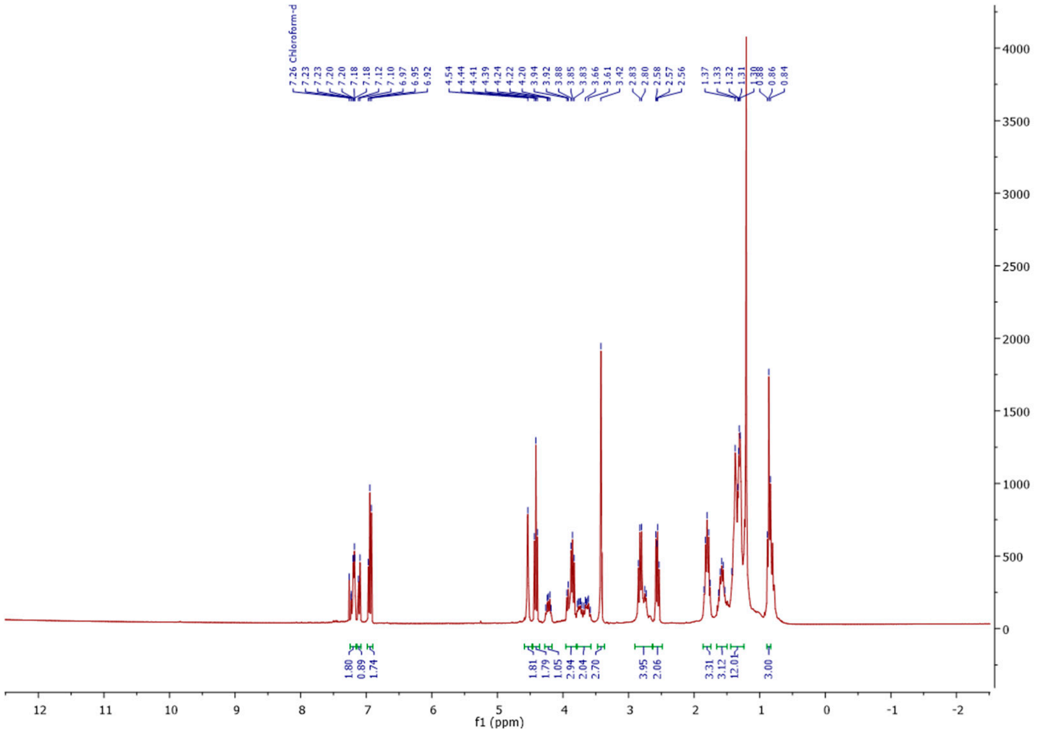

^{13}C NMR spectrum

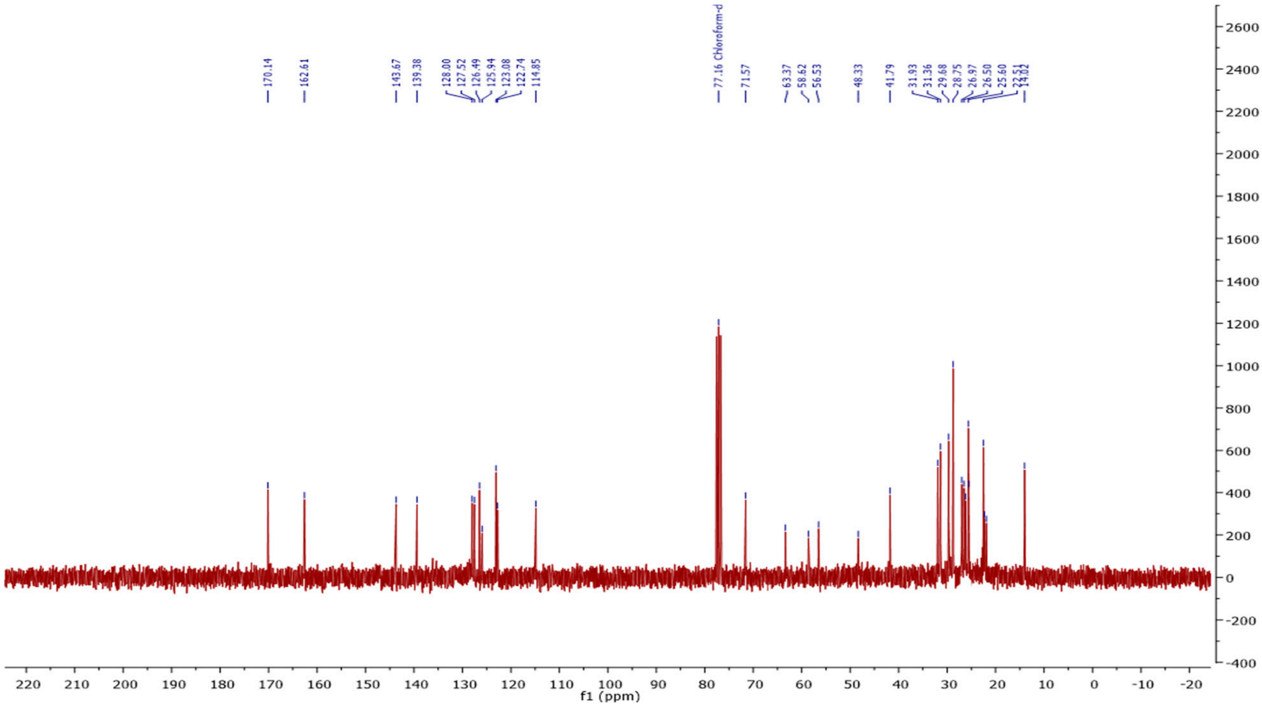

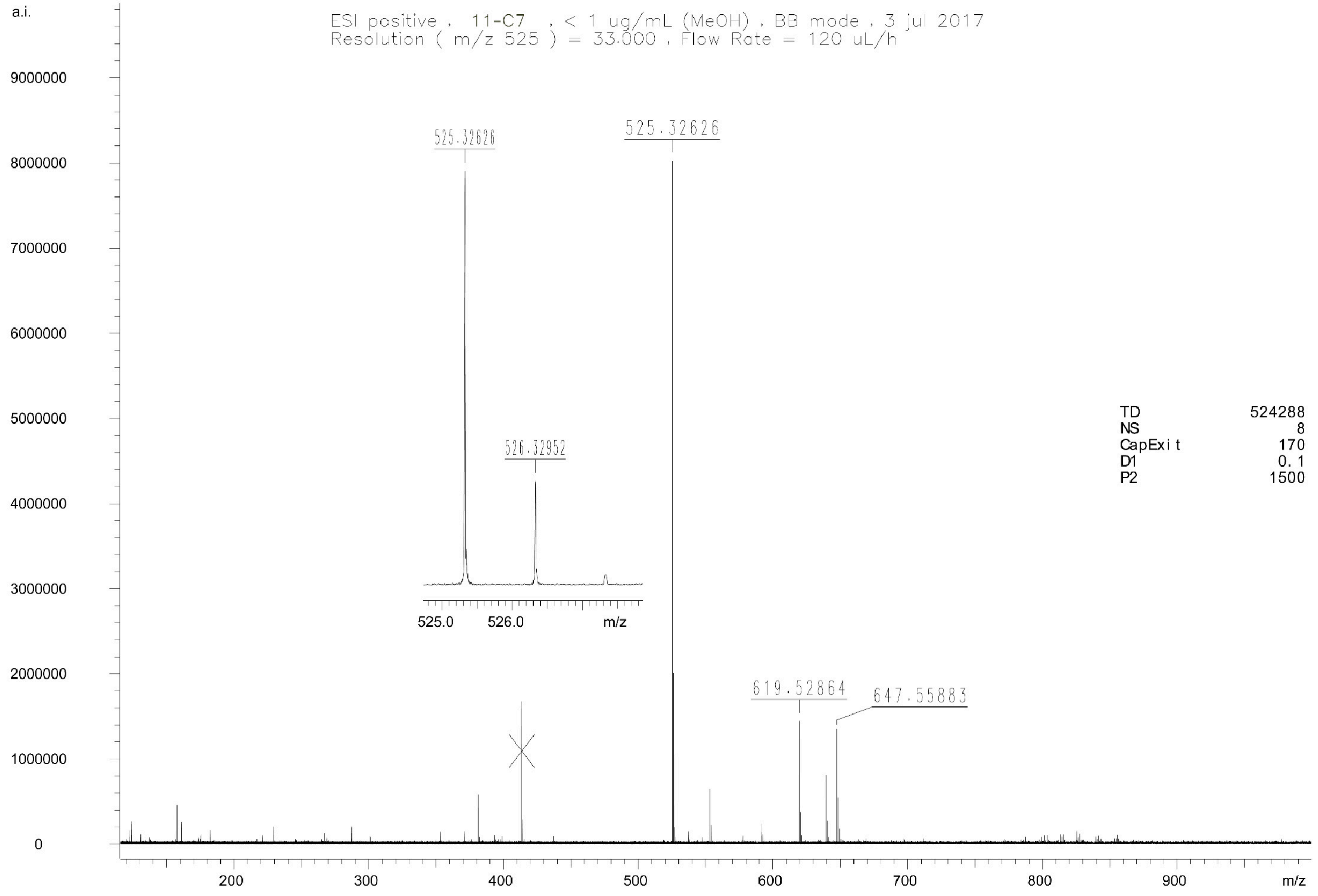
ESI positive , 11-C7 , < 1 ug/mL (MeOH) , BB mode , 3 jul 2017
Resolution (m/z 525) = 33.000 , Flow Rate = 120 uL/h
a.i.
9000000
8000000
7000000
6000000
5000000
4000000
3000000
2000000
1000000
0
525.32626
526.32952
525.0
526.0
m/z
525.32626
619.52864
647.55883
200
300
400
500
600
700
800
900
m/z
TD 524288
NS 8
CapExit 170
D1 0.1
P2 1500

13-C9

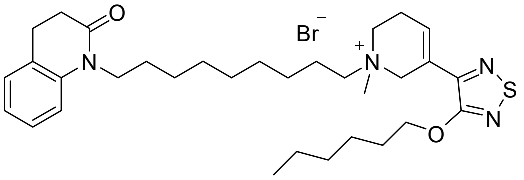

^{1}**H NMR** spectrum

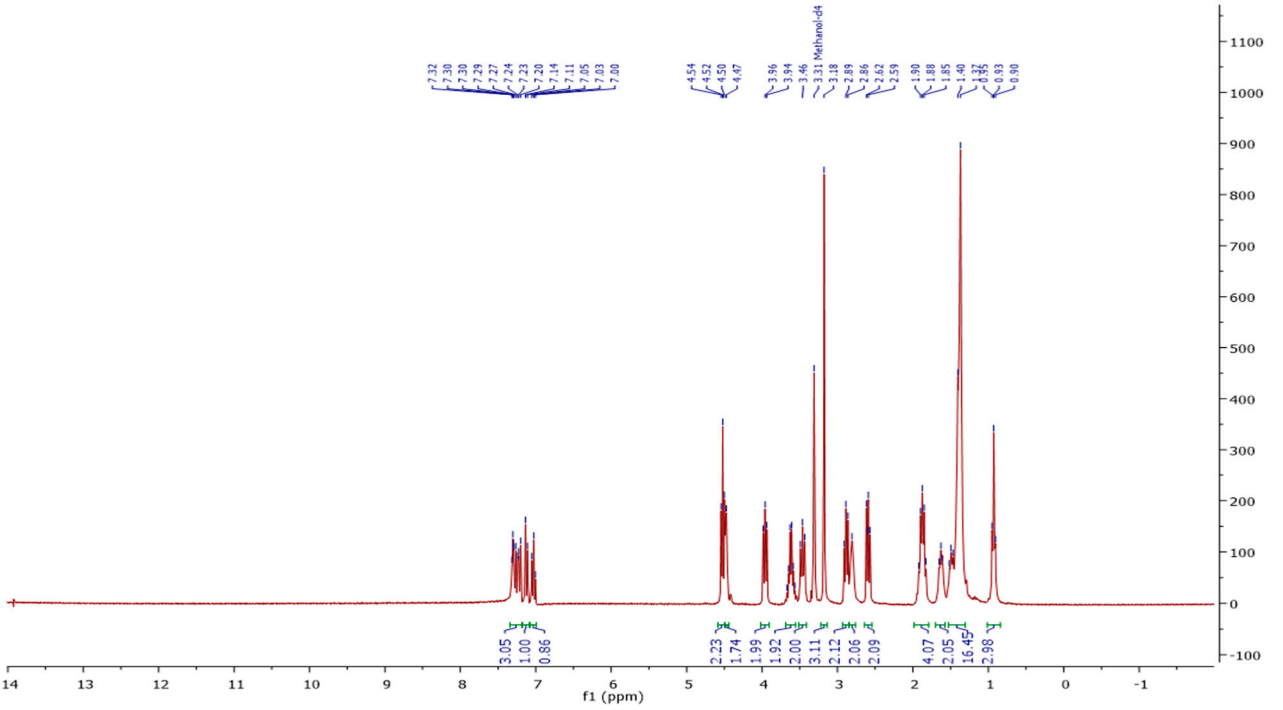

^{13}**C NMR** spectrum

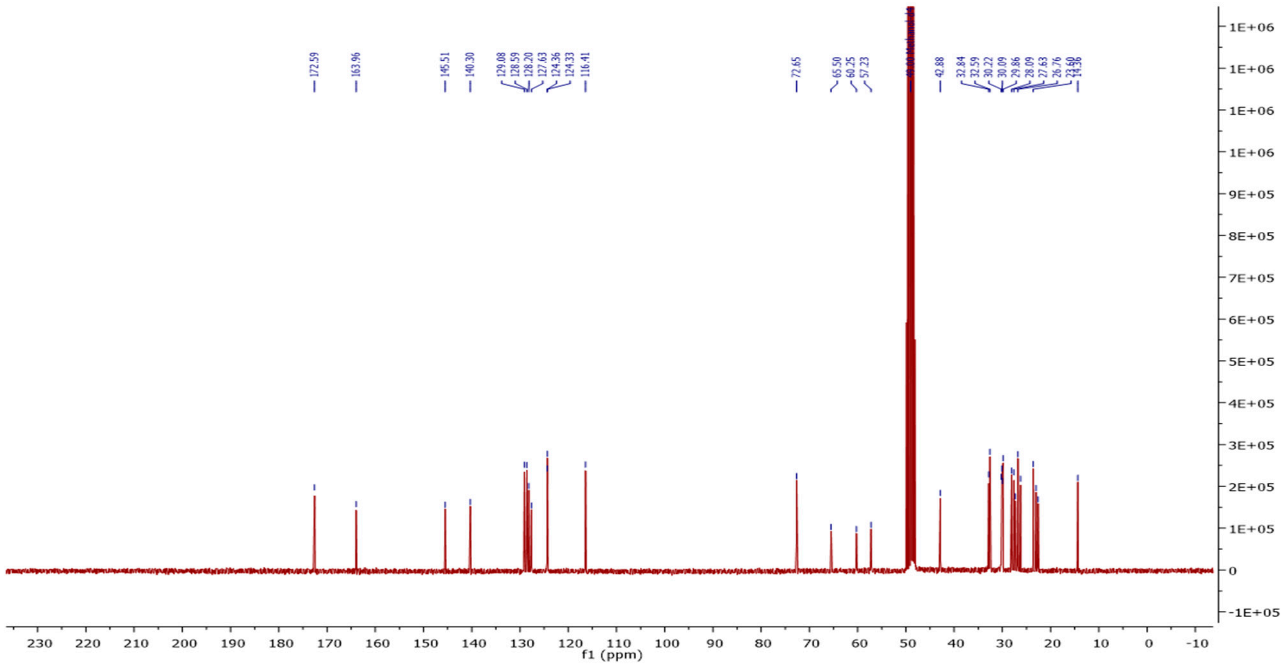

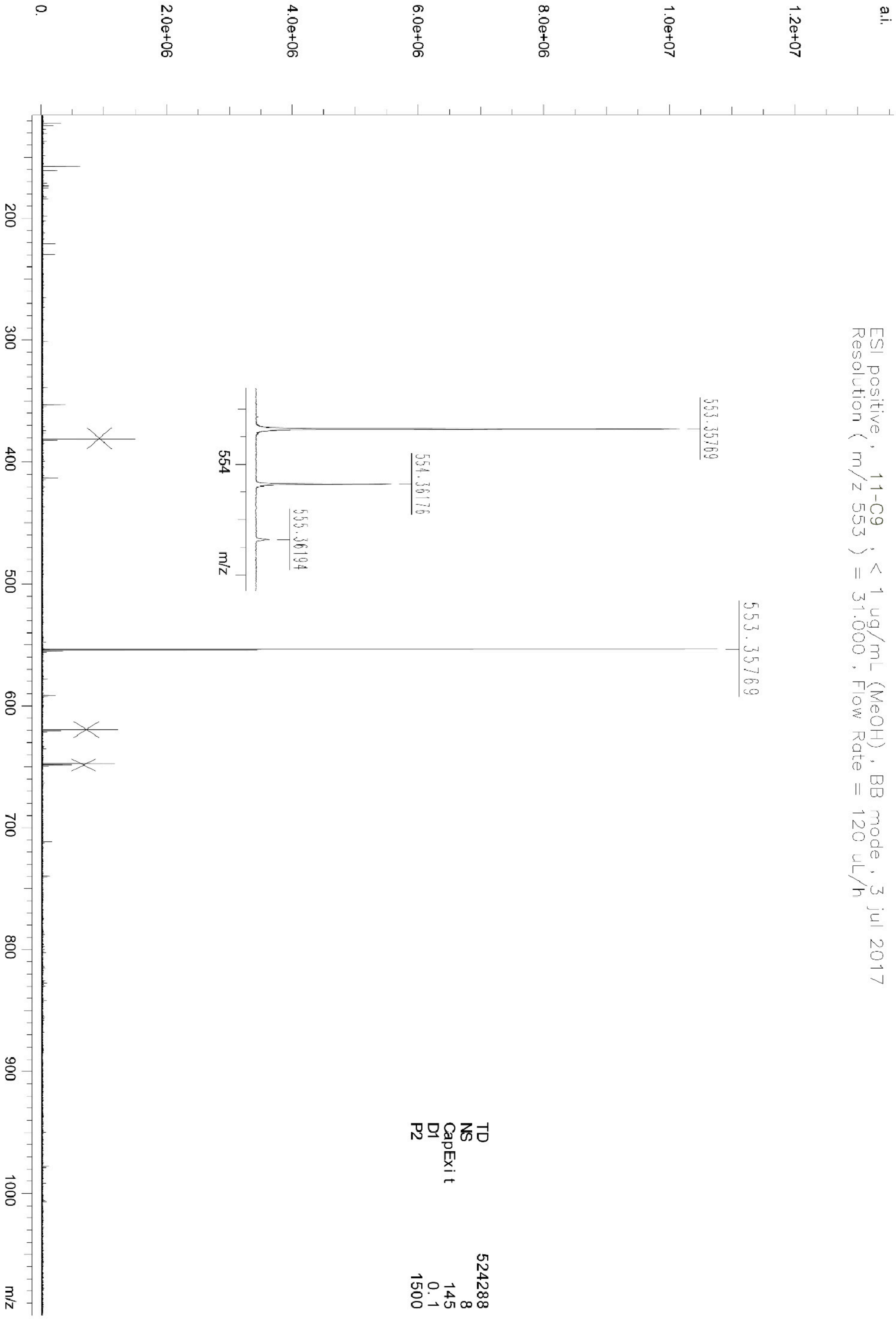
ESI positive , 11-C9 , < 1 ug/mL (MeOH) , BB mode , 3 jul 2017
Resolution (m/z 553) = 31.000 , Flow Rate = 120 uL/h
553.35769
554.36176
555.36194
554
m/z
TD 524288
NS 8
CapExit 145
D1 0.1
P2 1500
a.i.
1.2e+07
1.0e+07
8.0e+06
6.0e+06
4.0e+06
2.0e+06
0.
200
300
400
500
600
700
800
900
1000
m/z

12-C3

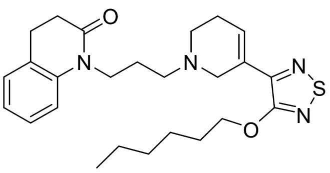

^{1}H NMR spectrum

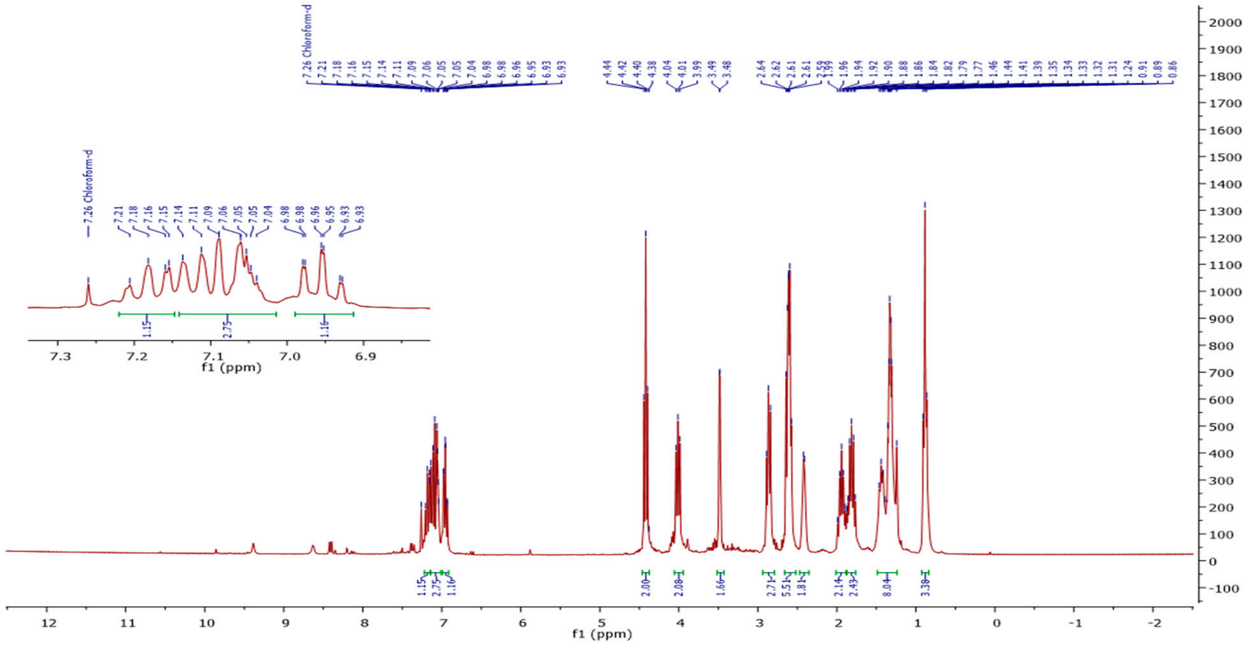

^{13}C NMR spectrum

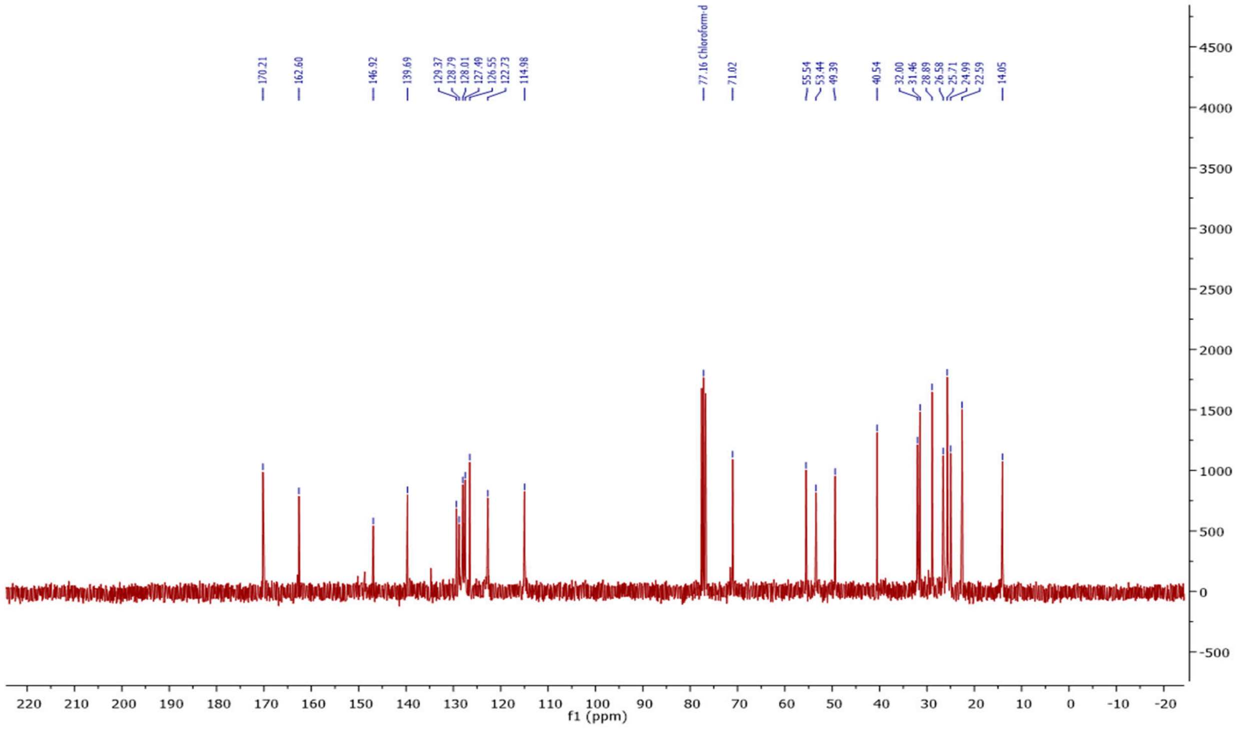

12-C3 Oxalate

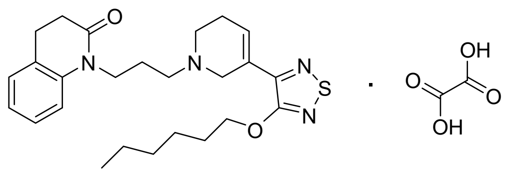

^{1}**H NMR** spectrum

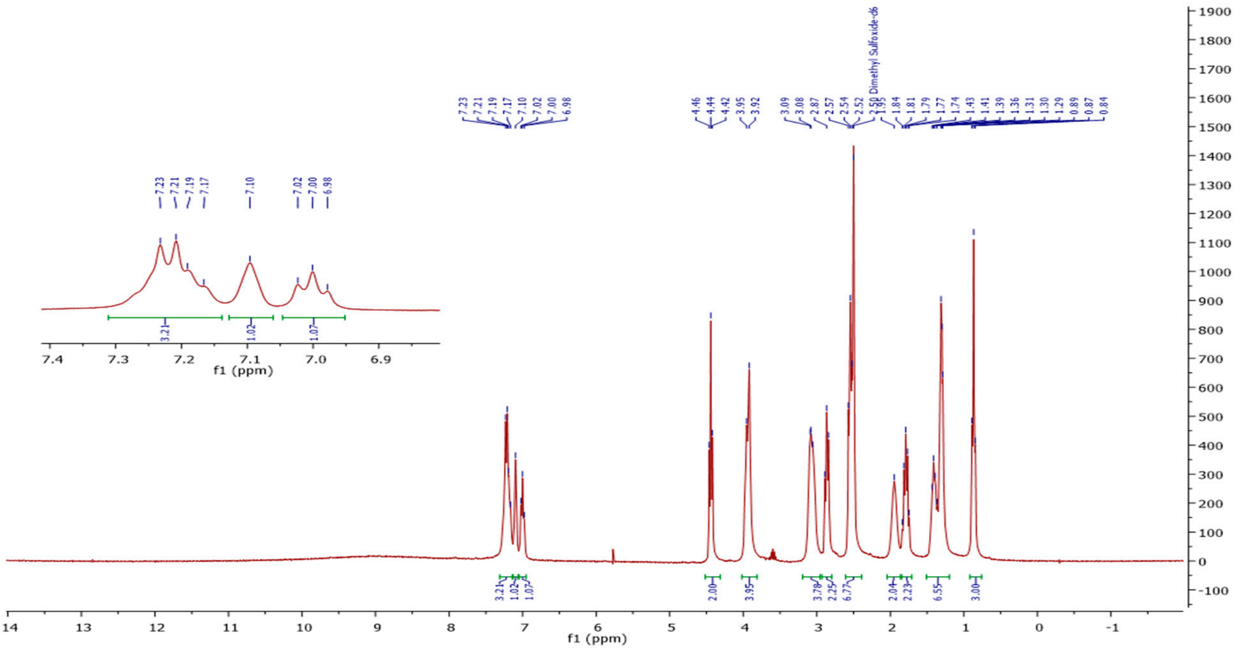

^{13}**C NMR** spectrum

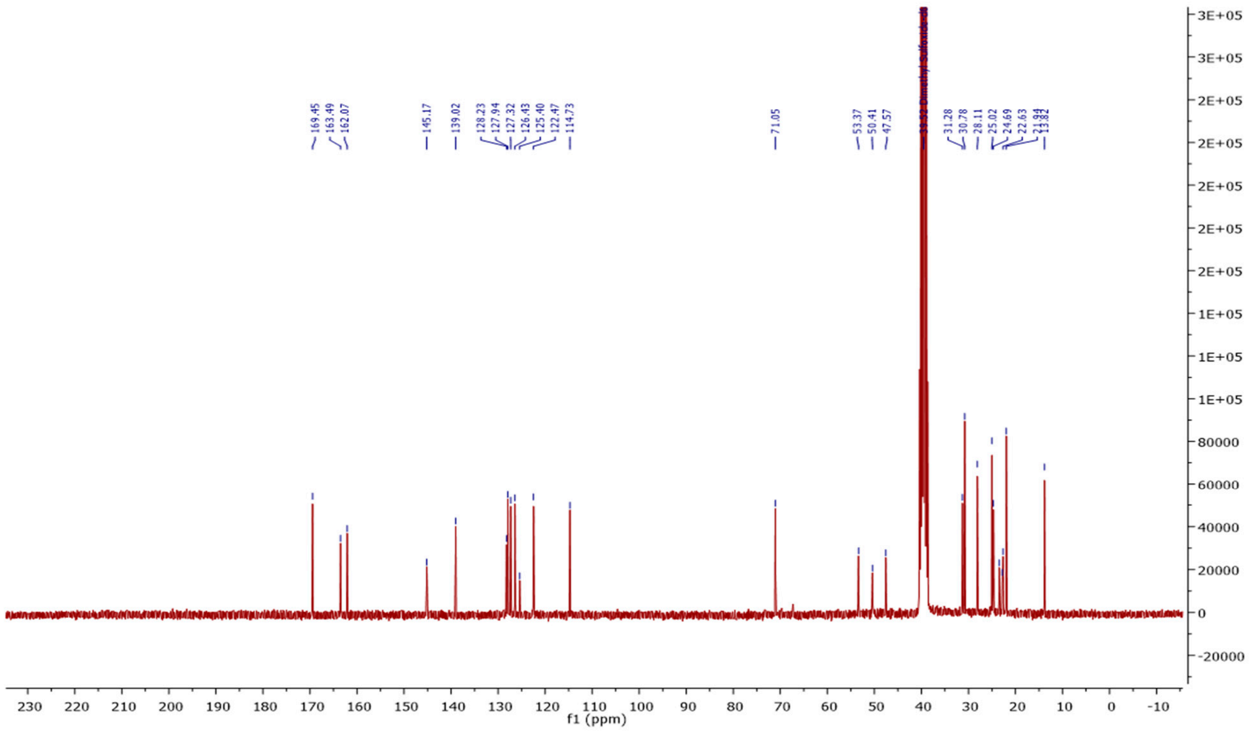

REDOX SRL

20900 MONZA (MB) - Viale Stucchi, 62/26
Tel.: 039.2847.434 - Fax: 039.2847.349
www.labredox.com
e-mail: info@labredox.com

Analytical Chemistry & Process Safety Testing

Codice Fiscale 06709300153
Partita IVA 00834700965
R.E.A. MB 1114665
Reg. Imp. Monza Brianza 06709300153

Certificato di Analisi
Certificate of Analysis

N°Campione/ Sample N°: 2016001294/LAB

Data apertura/ Registration date: 19/02/16

Richiedente/ Requested by:
Dallanoce

Committente/ Company:
UNIVERSITA' DI MILANO-Dip.Scienze Farm."Pietro Pratesi"

Campione/ Sample:
Q3X

Lotto/ Batch:

Analisi - Analysis<br>Metodo Analitico - Analytical method	Risultati<br>Results	Specifiche<br>Specifications
CHN<br>SOP - MET 019		
Carbonio	58.23%	
Idrogeno	6.57%	
Azoto	9.88%	

Note Chiusura/ Notes:

Calculated Elementary analysis for stoichiometry for 1:1
Anal. calcd for $C_{27}H_{36}N_4O_6S$: C, 59.54; H, 6.66; N, 10.29; found: C, 58,23; H, 6,57; N, 9.88.

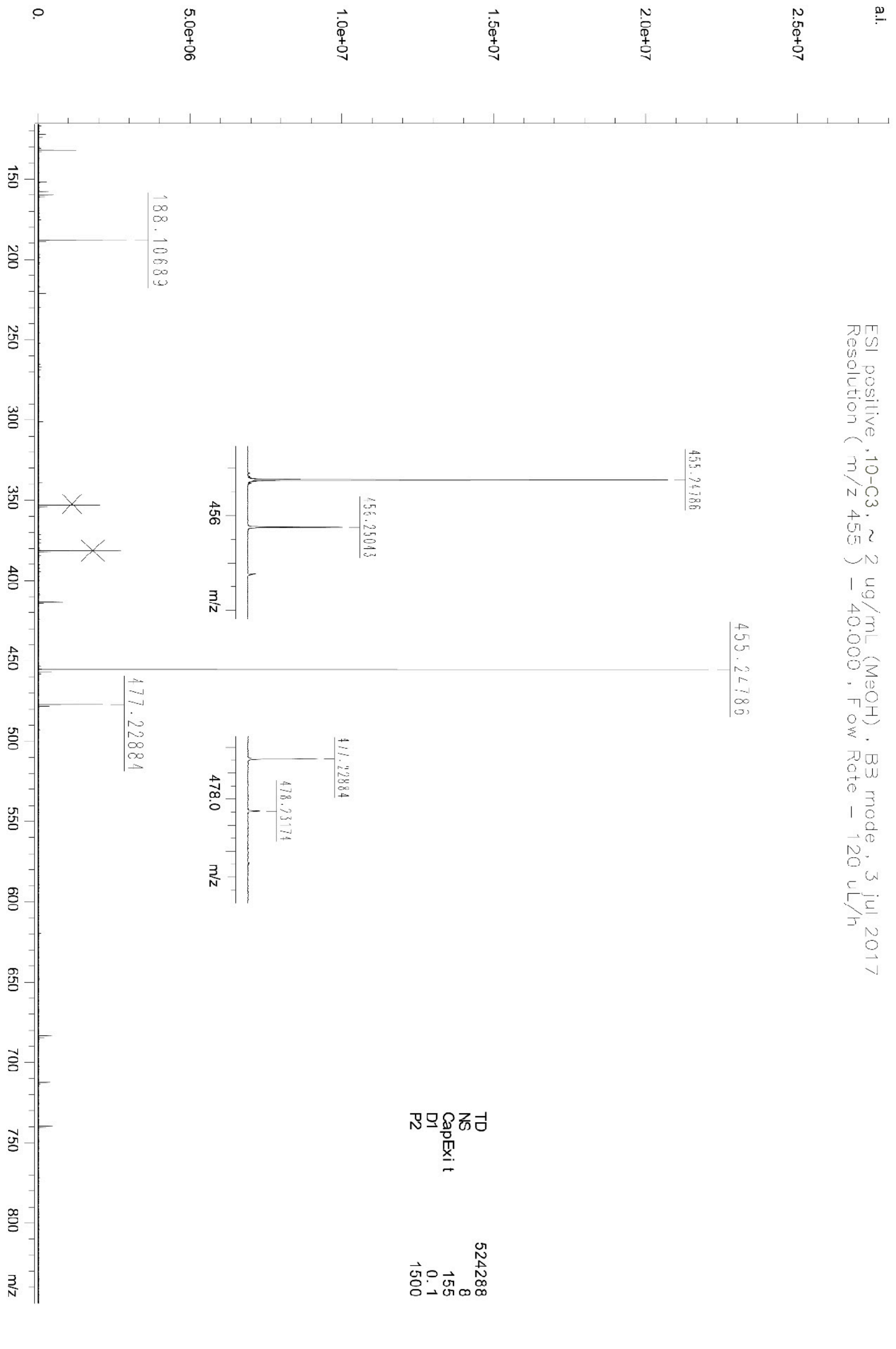
ESI positive ,10-C3, ~ 2 ug/mL (MeOH), BB mode, 3 jul 2017
Resolution (m/z 455) - 40.000, Fow Rate - 120 uL/h
a.i.
455.24786
456.25043
477.22884
478.23174
188.10683
456
478.0
m/z
TD 524288
NS 8
CapExi t 155
D1 0.1
P2 1500

12-C5

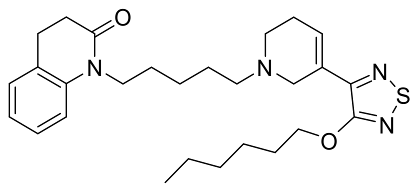

^{1}H NMR spectrum

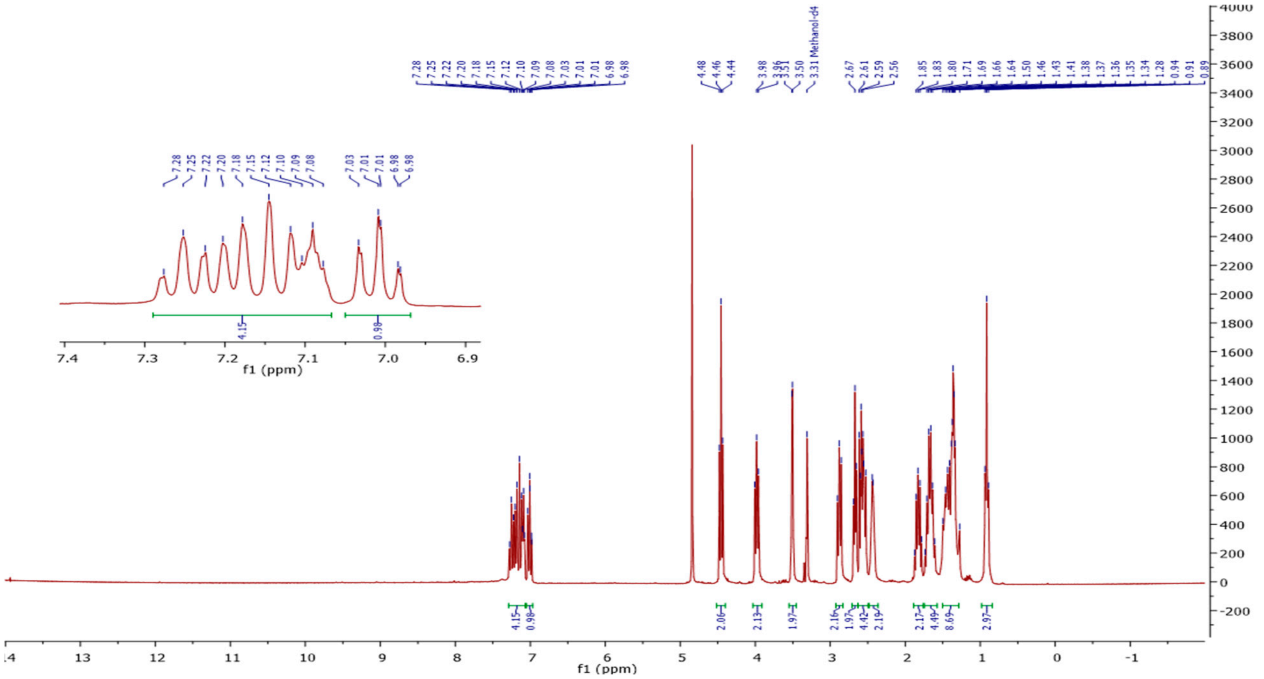

^{13}C NMR spectrum

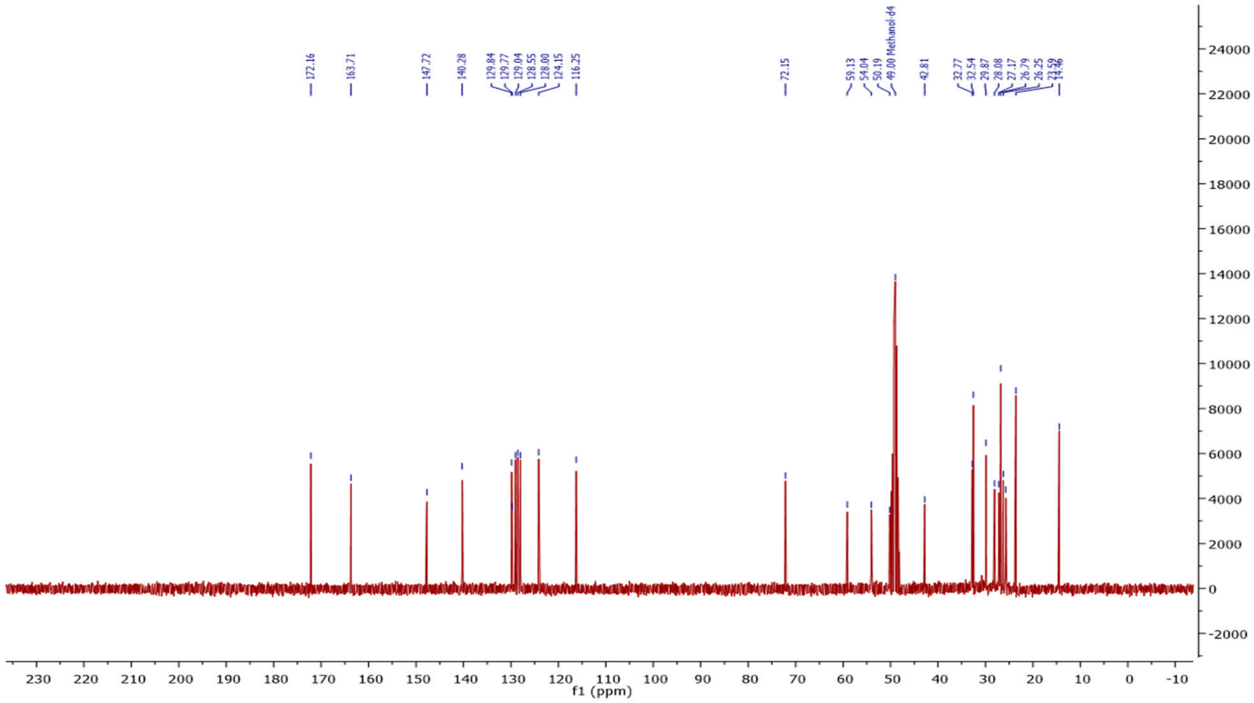

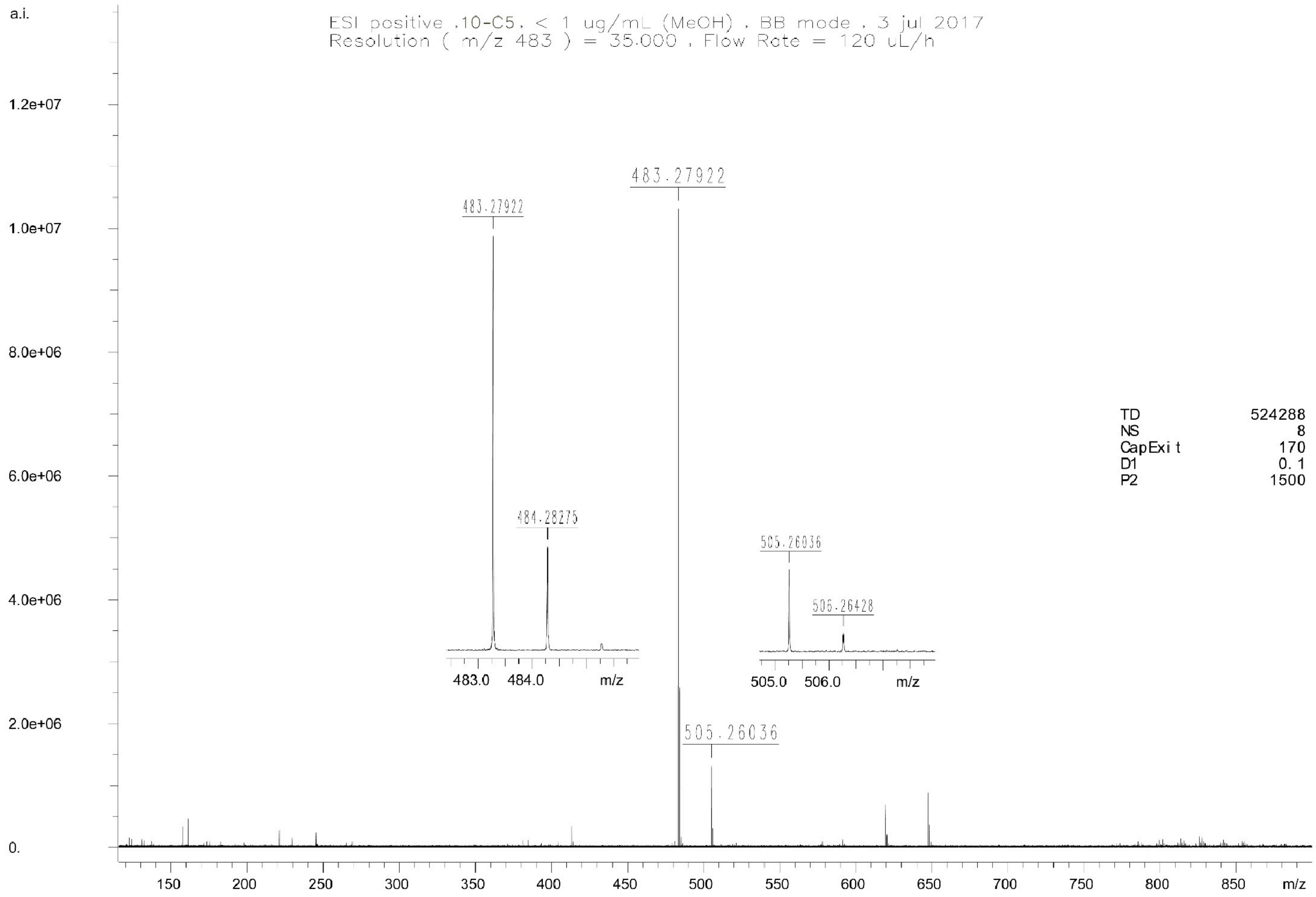
ESI positive ,10-C5, < 1 ug/mL (MeOH) , BB mode , 3 jul 2017
Resolution (m/z 483) = 35.000 , Flow Rate = 120 uL/h
a.i.
1.2e+07
1.0e+07
8.0e+06
6.0e+06
4.0e+06
2.0e+06
0.
483.27922
484.28275
505.26036
506.26428
483.0
484.0
505.0
506.0
m/z
150
200
250
300
350
400
450
500
550
600
650
700
750
800
850
TD 524288
NS 8
CapExit 170
D1 0.1
P2 1500

12-C7

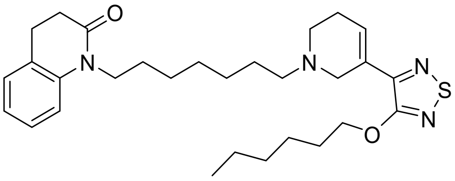

^{1}H NMR spectrum

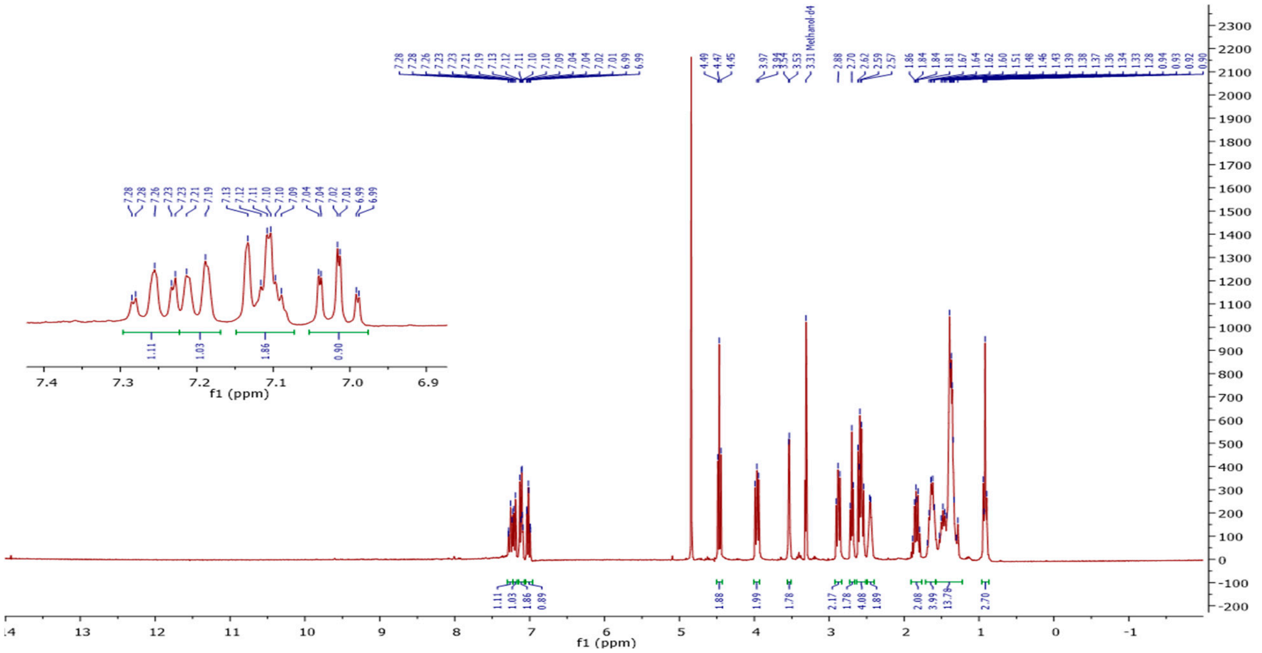

^{13}C NMR spectrum

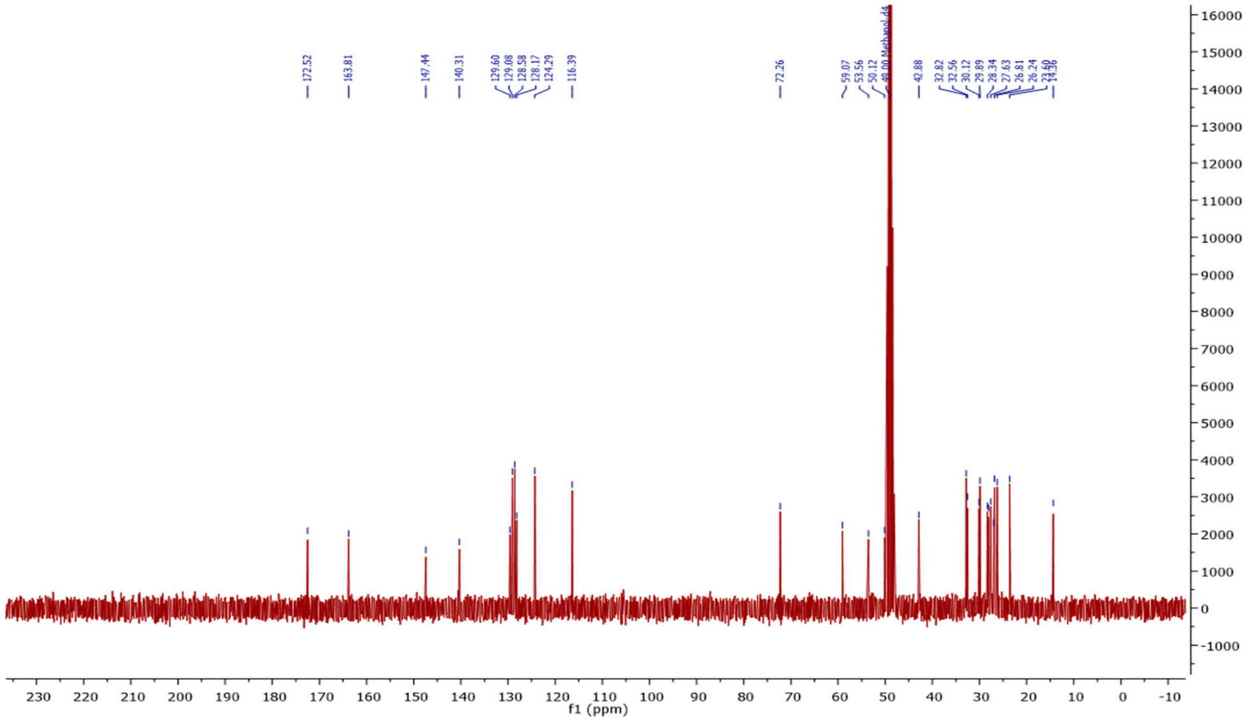

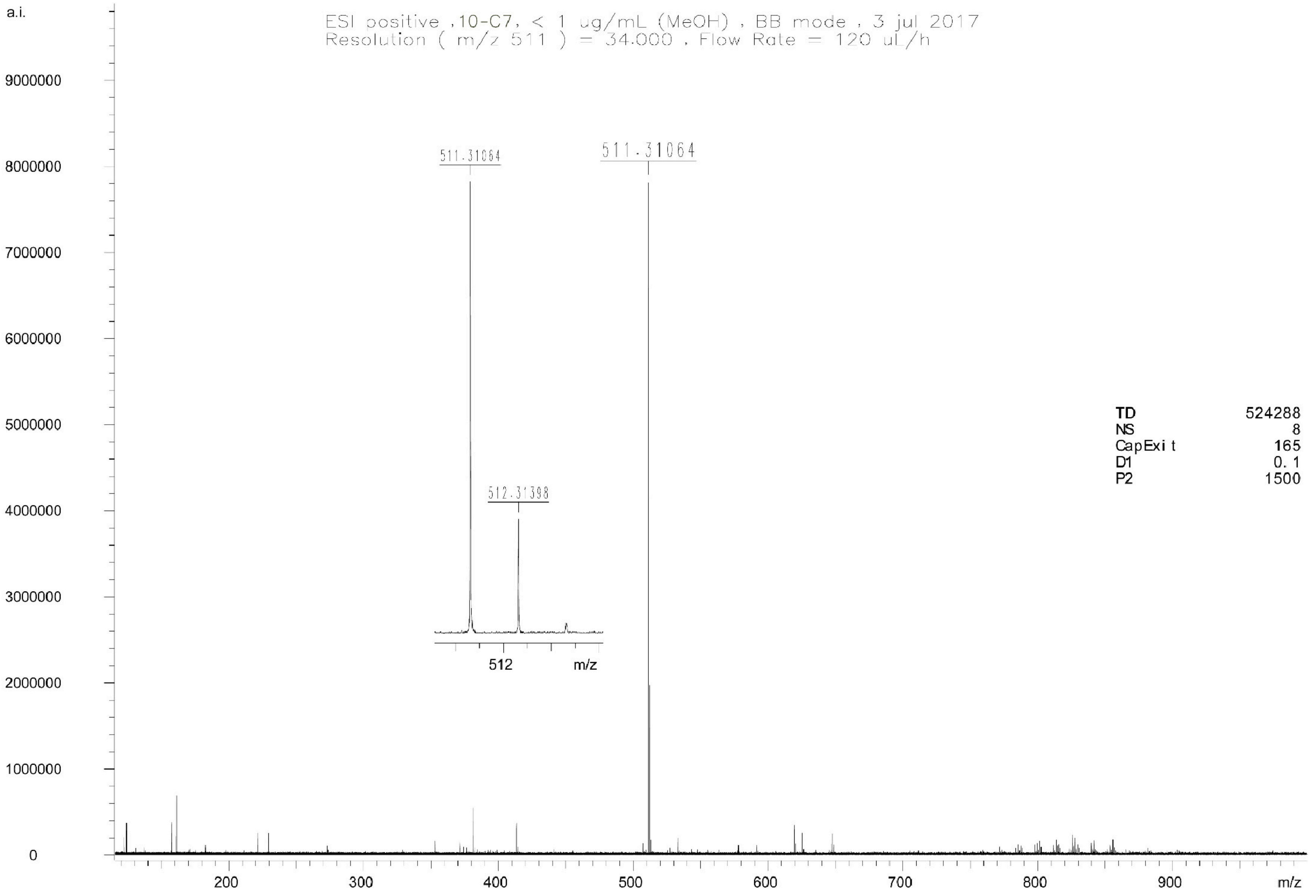
ESI positive ,10-C7, < 1 ug/mL (MeOH) , BB mode , 3 jul 2017
Resolution (m/z 511) = 34.000 , Flow Rate = 120 uL/h
a.i.
511.31064
512.31398
512
m/z
TD 524288
NS 8
CapExit 165
D1 0.1
P2 1500

12-C9

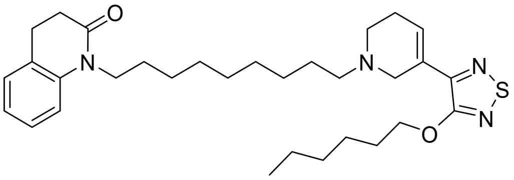

^{1}H NMR spectrum

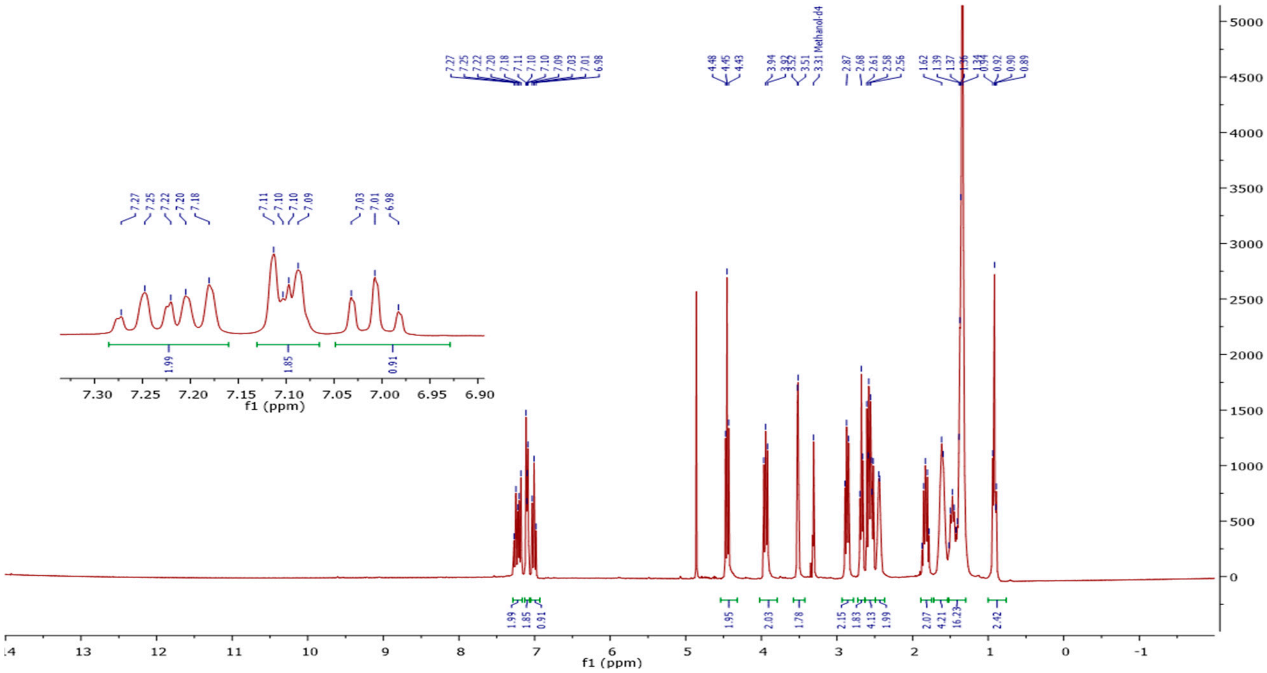

^{13}C NMR spectrum

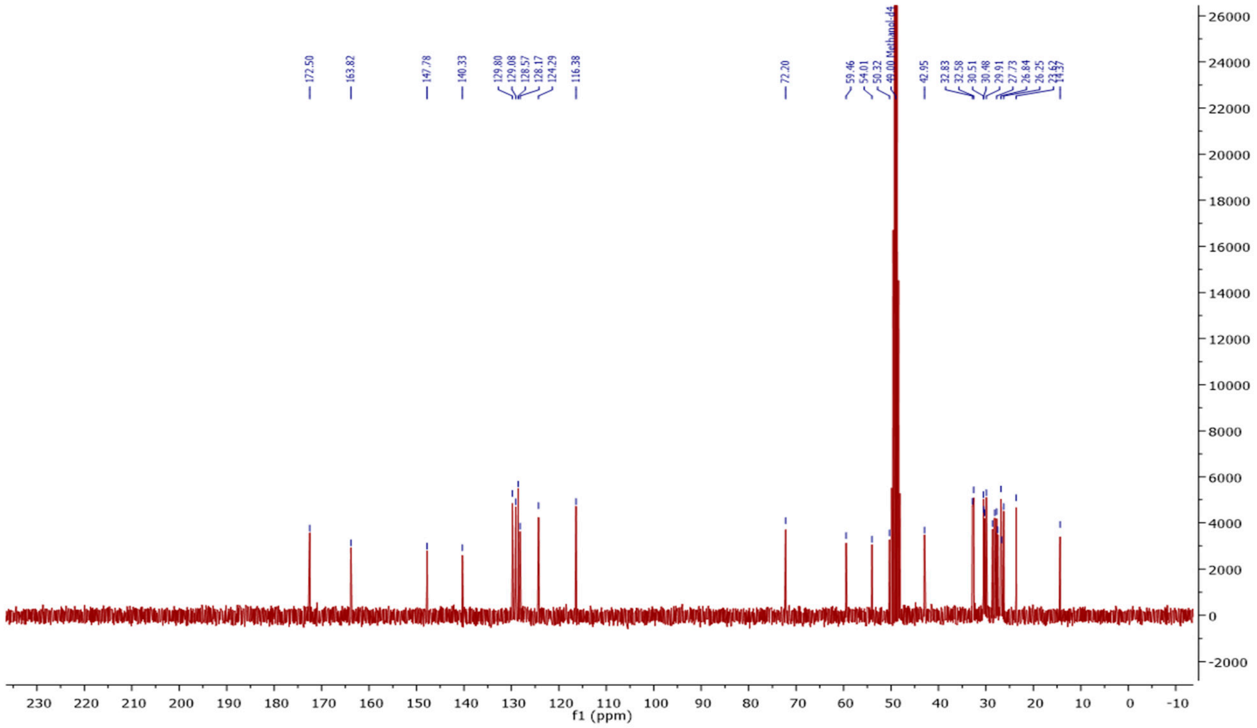

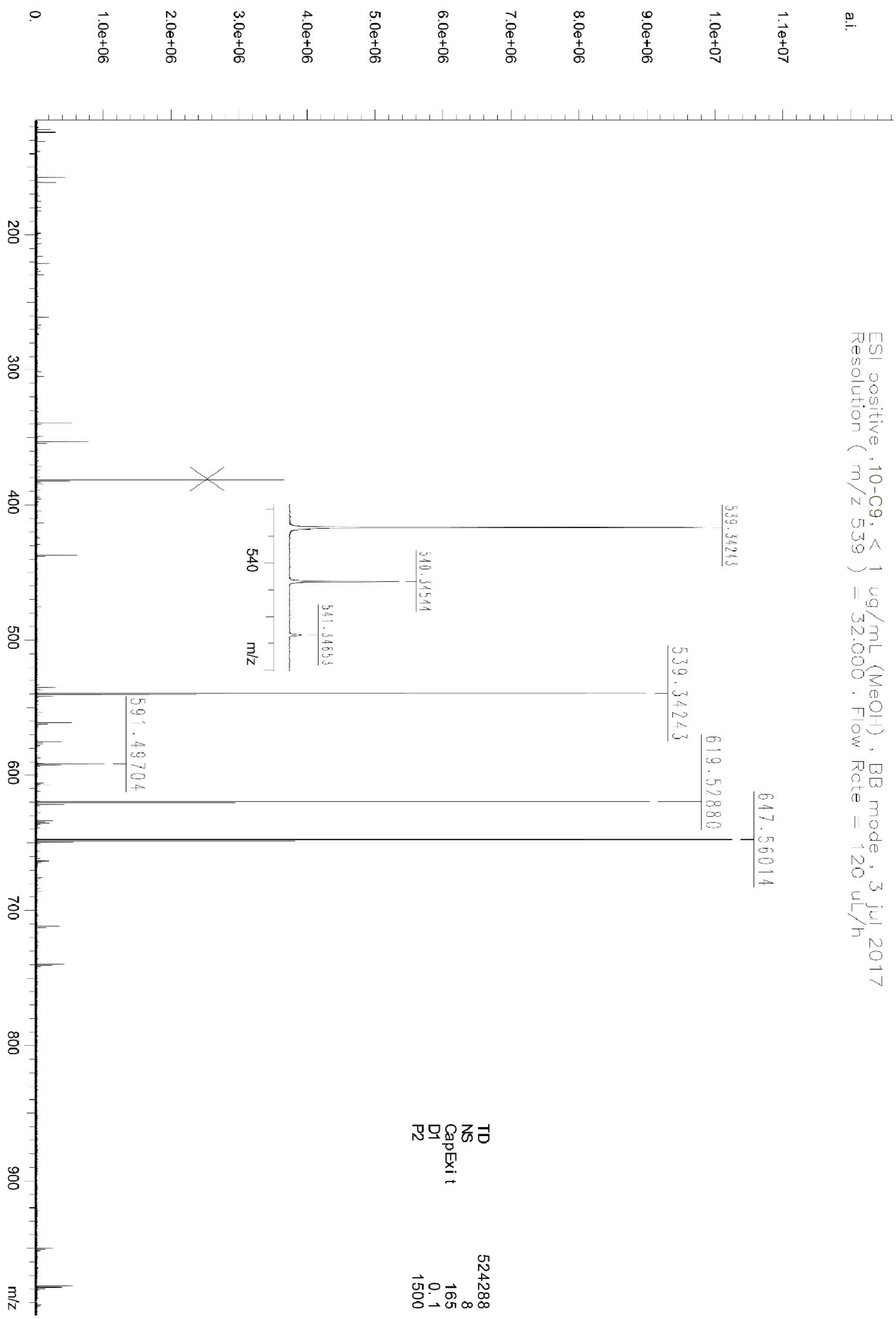
ESI positive ,10-C9, < 1 ug/mL (MeOH) , BB mode , 3 jul 2017
Resolution (m/z 539) = 32.000 , Flow Rate = 120 uL/h
539.34243
540.34544
541.34653
540
m/z
539.34243
591.49704
619.52880
647.56014
TD 524288
NS 8
CapExit 165
D1 0.1
P2 1500
a.i.
m/z
